# Cheese consumption and multiple health outcomes: an umbrella review and updated meta-analysis of prospective studies

**DOI:** 10.1016/j.advnut.2023.06.007

**Published:** 2023-06-15

**Authors:** Mingjie Zhang, Xiaocong Dong, Zihui Huang, Xue Li, Yue Zhao, Yingyao Wang, Huilian Zhu, Aiping Fang, Edward L. Giovannucci

**Affiliations:** 1 Department of Nutrition, School of Public Health, Sun Yat-sen University, Guangzhou, China; 2 Department of Big Data in Health Science School of Public Health, Center of Clinical Big Data and Analytics of The Second Affiliated Hospital, Zhejiang University School of Medicine, Hangzhou, China; 3 Chinese Nutrition Society Academy of Nutrition and Health, Beijing, China; 4 Guangdong Provincial Key Laboratory of Food, Nutrition and Health, School of Public Health, Sun Yat-sen University, Guangzhou, China; 5 Department of Nutrition, Harvard T.H. Chan School of Public Health, Boston, Massachusetts, USA; 6 Department of Epidemiology, Harvard T.H. Chan School of Public Health, Boston, Massachusetts, USA

**Keywords:** cheese consumption, umbrella review, updated meta-analyses, all-cause mortality, cardiovascular disease, cancer, fracture, metabolic disease

## Abstract

This umbrella review aims to provide a systematic and comprehensive overview of current evidence from prospective studies on the diverse health effects of cheese consumption. We searched PubMed, Embase, and Cochrane Library to identify meta-analyses/pooled analyses of prospective studies examining the association between cheese consumption and major health outcomes from inception to August 31, 2022. We reanalyzed and updated previous meta-analyses and performed de novo meta-analyses with recently published prospective studies, where appropriate. We calculated the summary effect size, 95% prediction confidence intervals, between-study heterogeneity, small-study effects, and excess significance bias for each health outcome. We identified 54 eligible articles of meta-analyses/pooled analyses. After adding newly published original articles, we performed 35 updated meta-analyses and 4 de novo meta-analyses. Together with 8 previous meta-analyses, we finally included 47 unique health outcomes. Cheese consumption was inversely associated with all-cause mortality (highest compared with lowest category: RR = 0.95; 95% CI: 0.92, 0.99), cardiovascular mortality (RR = 0.93; 95% CI: 0.88, 0.99), incident cardiovascular disease (CVD) (RR = 0.92; 95% CI: 0.89, 0.96), coronary heart disease (CHD) (RR = 0.92; 95% CI: 0.86, 0.98), stroke (RR = 0.93; 95% CI: 0.89, 0.98), estrogen receptor-negative (ER−) breast cancer (RR = 0.89; 95% CI: 0.82, 0.97), type 2 diabetes (RR = 0.93; 95% CI: 0.88, 0.98), total fracture (RR = 0.90; 95% CI: 0.86, 0.95), and dementia (RR = 0.81; 95% CI: 0.66, 0.99). Null associations were found for other outcomes. According to the NutriGrade scoring system, moderate quality of evidence was observed for inverse associations of cheese consumption with all-cause and cardiovascular mortality, incident CVD, CHD, and stroke, and for null associations with cancer mortality, incident hypertension, and prostate cancer. Our findings suggest that cheese consumption has neutral to moderate benefits for human health.

**Statement of significance**

This umbrella review provides a systematic and comprehensive overview of current evidence on the association of cheese consumption with 47 major health outcomes through 35 updated, 4 de novo, and 8 previous meta-analyses of prospective observational studies. Although high saturated fat and sodium in some cheeses tend to be emphasized as a health concern in dietary guidelines, cheese also provides some nutrients and bioactive compounds that may confer some benefits.

## Introduction

Cheese is generally a nutrient-dense and well-tolerated fermented dairy product consumed worldwide. However, the health effects of cheese consumption remain a matter of controversy. On one hand, cheese is a rich source of high-quality protein (mainly casein), lipids, minerals (e.g., calcium, phosphorus, and magnesium), and vitamins (e.g., vitamin A, K_2_, B_2_, B_12_, and folate), and probiotics and bioactive molecules (e.g., bioactive peptides, lactoferrin, short-chain fatty acids, and milk fat globule membrane), which may provide various health benefits. On the other hand, cheese contains relatively high contents of saturated fat and salt, which are perceived as unfavorable dietary components for cardiovascular health [1,2]. Currently, most dietary guidelines recommend consuming dairy products as part of a healthy diet while avoiding intake of full-fat and high-sodium versions [[3], [4], [5], [6]]. Of note, the recommendation is primarily based on extrapolated benefits and harms of single nutrient contained in dairy. However, whole dairy foods are not a simple collection of isolated nutrients but have complex physical and nutritional structures (i.e., dairy matrix), which affect digestibility and nutrient bioavailability, thereby modifying the overall effects of dairy consumption on health and disease [[7], [8], [9]]. In addition, dairy products are a heterogeneous group of foods regarding the dairy matrix due to different processing methods [8]. Because various types of dairy products appear to have distinct influences on specific health outcomes [10], merging them into 1 group (i.e., total dairy consumption) may blur the true association. Thus, a separate assessment of the health effects of cheese consumption is required.

Umbrella reviews can provide a comprehensive overview of evidence from existing meta-analyses on a given topic, with unique strengths of identifying the uncertainties, biases, and knowledge gaps of the evidence [11]. Many meta-analyses on the association between cheese consumption and a range of health end points, such as all-cause and cause-specific mortality, cardiovascular diseases (CVD), cancer, metabolic diseases, bone fracture, and other diseases, have been published [[12], [13], [14], [15], [16], [17]]. An extensive summary of the breadth and validity of these associations with diverse health outcomes will help elucidate the role of cheese consumption in human health. Therefore, we conducted an umbrella review to synthesize the available evidence from meta-analyses of prospective studies to examine the various health impacts of cheese consumption. Furthermore, we contextualized the magnitude, direction, and significance of the identified associations, evaluated risk of potential biases, and assessed the credibility of the evidence.

## Methods

The present umbrella review was conducted following the Preferred Reporting Items for Systematic Reviews and Meta-Analyses (PRISMA) guidelines [18]. The protocol of this study was registered in PROSPERO (CRD42022331328).

### Literature search

We systematically searched PubMed, Embase, and Cochrane Library databases to identify existing meta-analyses (including pooled analyses) of prospective studies investigating the association between cheese consumption and any health outcome from inception to August 31, 2022. The search terms were as follows: (cheese) AND (“meta analysis” OR meta-analysis OR “meta analyzed” OR meta-analyzed OR “pooled analysis” OR “systematic review”). We also extensively searched the 3 databases for recently published original prospective studies to update previous meta-analyses or derive de novo meta-analyses. Predefined search strategies for meta-analyses and primary studies are presented in Supplemental Tables 1 and 2. Two investigators (XD, MZ) independently performed a 3-step parallel screening of titles, abstracts, and full texts for all identified studies according to the inclusion and exclusion criteria. Any discrepancies were discussed and resolved by a third investigator (ZH).

### Eligibility criteria

Meta-analyses of population-based prospective studies (i.e., prospective cohort studies, case-cohort studies, nested case–control studies, and randomized controlled trials) exploring the association between cheese consumption (primary or secondary exposure of interest) and major health outcomes were included in the umbrella review. Original prospective studies eligible for updated or de novo meta-analyses were also included. Conference abstracts, interviews, letters, and narrative reviews were excluded. Meta-analyses or original studies without full text, effect size [e.g., risk ratio (RR), odds ratio (OR), or hazard ratio (HR)], or not written in English were also excluded. Studies with changes in cheese consumption rather than absolute intake as exposure, using substitution analysis, or using surrogate end points (e.g., blood lipids, blood pressure, and body weight) as outcomes were removed. If more than 1 article reported the results for an identical outcome from the same study population (or cohort), only the one with the largest sample size, the longest follow-up, or the most complete information was included.

### Data extraction

From each included meta-analysis, the following information was extracted and verified by three investigators (XD, MZ, ZH): the first author’s name, publication year, outcome of interest, study population (general or disease status), study design of the primary studies, type of comparison (highest compared with lowest category of cheese consumption or each increment in cheese consumption), number of included studies, number of participants and cases, and the reported summary risk estimates (RR, OR, or HR) with corresponding 95% CIs. For meta-analyses on over 1 health outcome, each outcome was recorded separately. For original studies, the extracted data covered information on the first author’s name, publication year, study design, study population characteristics, geographic location, number of participants and cases, length of follow-up (cohort study), dietary assessment method (e.g., food frequency questionnaire and 3-d 24-h dietary records), cheese type, categorization and amount of cheese consumption, adjustment factors, and effect size with 95% CIs.

### Evaluation of methodological quality

The AMSTAR-2 (A Measurement Tool to Assess Systematic Reviews) tool [19] was used to evaluate the methodological quality of the included published meta-analyses and systematic reviews. It includes 16 individual items and 7 of them are identified as critical. Systematic reviews with no or 1 noncritical weakness are rated as high confidence; those with more than 1 noncritical weakness are rated as moderate confidence; those with 1 critical flaw with or without noncritical weaknesses are rated as low confidence; and those with more than 1 critical flaw with or without noncritical weaknesses are rated as critically low confidence. Two investigators (XD, MZ) implemented the evaluation independently, with disagreements reconciled by discussion and consensus.

### Assessment of evidence credibility

The credibility of evidence was assessed using the NutriGrade scoring system [20]. It comprised 8 items: risk of bias of the primary studies (0–2 points), precision of the estimate (0–1 point), heterogeneity (0–1 point), directness (0–1 point), publication bias (0–1 point), funding bias (0–1 point), effect size (0–2 points), and dose–response association (0–1 point). The meta-evidence was graded as high, moderate, low, and very low if the overall score is ≥8 points, 6 to <8 points, 4 to <6 points, and <4 points, respectively. Two investigators (XD, MZ) independently performed the rating and any discrepancies were resolved by discussion.

### Statistical analysis

We reanalyzed previous meta-analyses to obtain necessary information for subsequent assessment of the credibility of evidence. If the existing meta-analysis included cross-sectional, retrospective, and prospective studies, we only kept the results from prospective studies in our meta-analysis. Furthermore, we incorporated newly identified original studies into previous meta-analyses to update or derive de novo meta-analyses, where appropriate. For each outcome, we recalculated the summary risk estimates and their corresponding 95% CIs for the highest compared with the lowest category of cheese consumption and/or per 30-g/d increment in cheese consumption by using the random-effects model by DerSimonian and Laird [21]. When results from the same cohort were reported separately for different cheese types (e.g., hard cheese and cottage cheese or low-fat cheese and high-fat cheese instead of total cheese) and disease subtypes [e.g., coronary heart disease (CHD) and stroke rather than total CVD], we used a fixed-effects model to generate an overall estimate before pooling with other studies.

Heterogeneity across studies was investigated using *I*^2^ statistic. We also performed subgroup analyses according to adjustment for total energy intake in the models (adjusted and unadjusted) and geographical location (Europe, North America, and Oceania; Asia and other regions; multiregion) to explore potential sources of heterogeneity. We computed 95% prediction intervals (95% PIs) to predict the effect size range of a future original study will lie after considering both the uncertainty in the mean effect and the heterogeneity from the random-effects model [22,23]. We assessed potential small-study effects by Egger’s test [24] if ≥3 studies were available. A *P* value of <0.10 was interpreted as the presence of small-study effects. The excess statistical significance test was performed to evaluate whether the observed number of nominally statistically significant studies was larger than their expected number using the χ^2^ test [25]. A *P* value of <0.10 was considered statistically significant.

We further examined the nonlinear dose–response association among studies that provided risk estimates with ≥3 exposure categories using a 2-stage restricted cubic splines (3 knots at 25, 50, and 75 percentiles) analysis [26,27]. The *P* value for nonlinearity was calculated by testing whether the coefficient of the second spline was equal to 0 [27]. We used the median/mean of each consumption category if available or the midpoint between the lower and upper bounds of each intake category to represent the intake levels. We assumed zero as the lower bound for the open-ended lowest category and multiplied the lower bound value by 1.2 as the upper bound for the open-ended highest category. All statistical analyses were conducted using the “metafor,” “meta,” “dosresmeta,” and “forestplot” packages in R software version 4.1.0 (The R Foundation).

## Results

**FIGURE 1:**
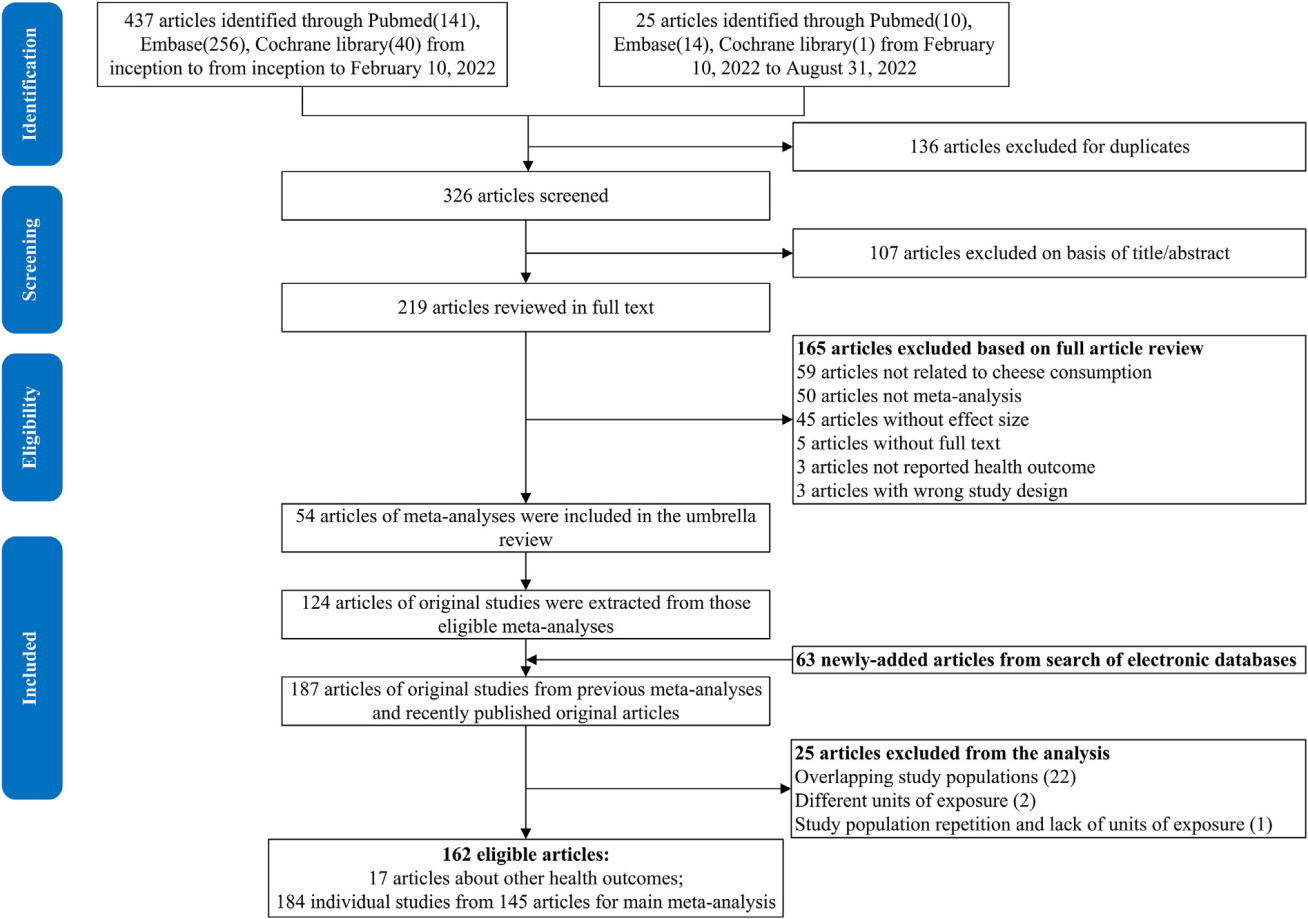
Flow diagram of the study search and selection process.

**FIGURE 2:**
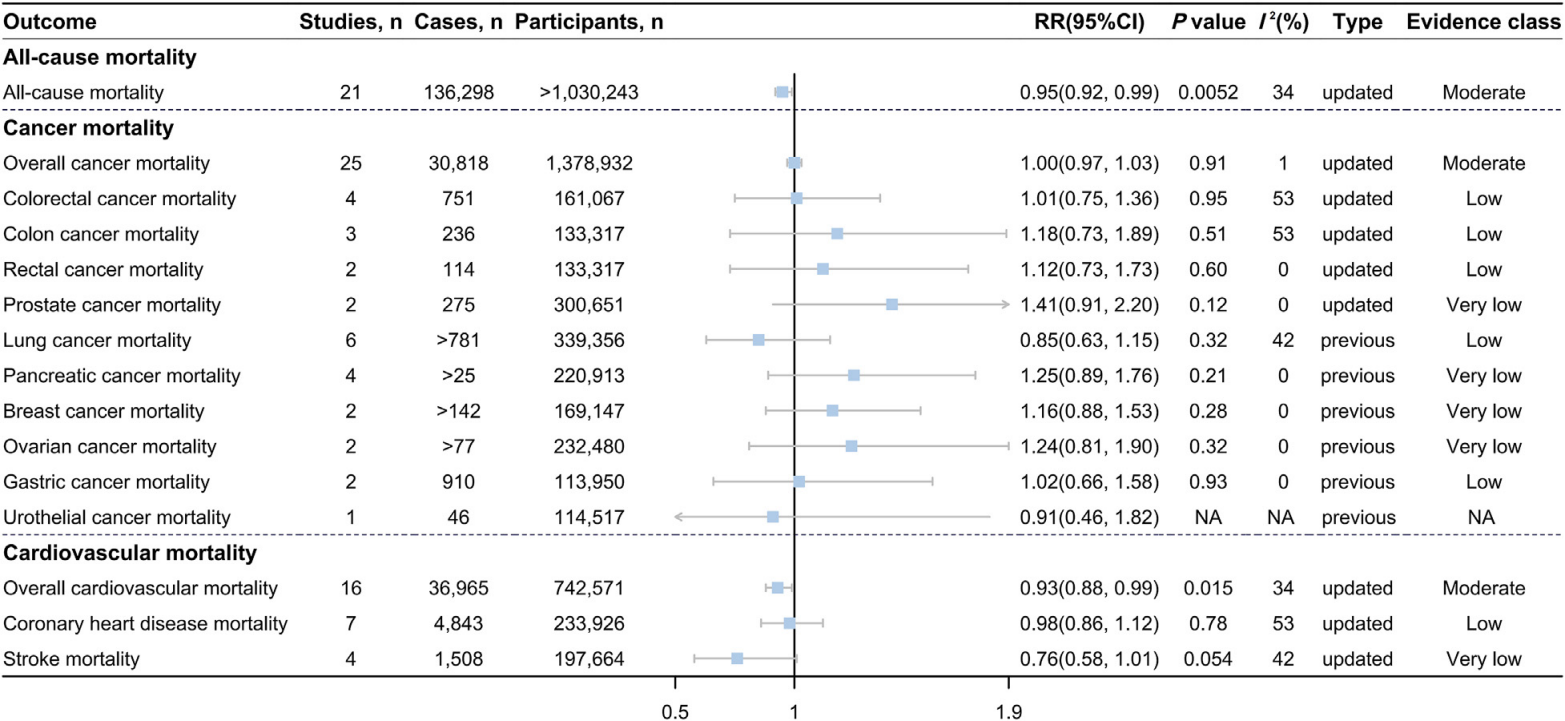
Association between cheese consumption (highest compared with lowest intake level) and all-cause and cause-specific mortality.

**FIGURE 3:**
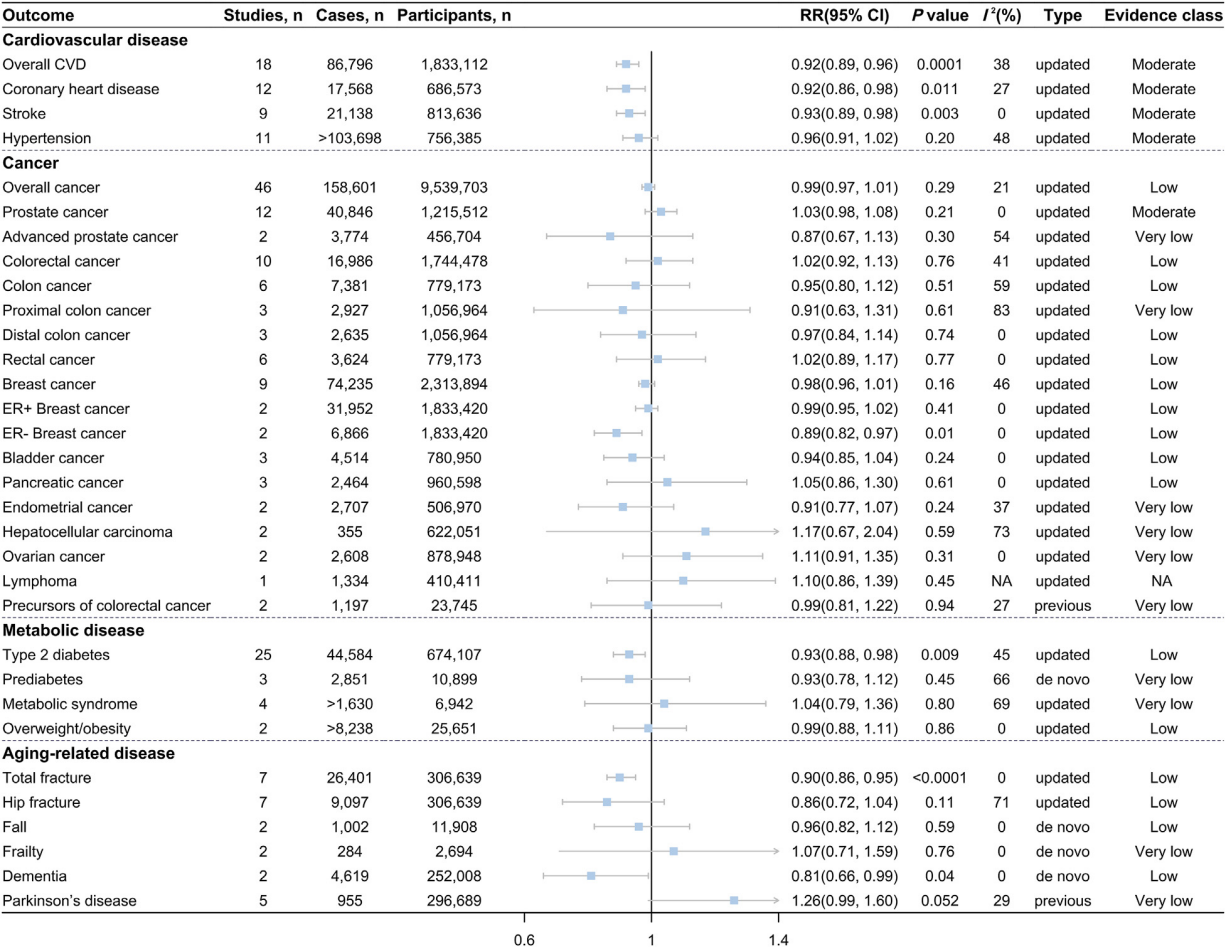
Association between cheese consumption (highest compared with lowest intake level) and disease incidence.

Figure 1 shows the flowchart of the study search and selection process. We identified 54 eligible articles of meta-analyses/pooled analyses on 34 health outcomes, including 11 mortality outcomes [12,[28], [29], [30], [31], [32], [33], [34]] (Supplemental Table 3), 4 CVD outcomes [13,28,[35], [36], [37], [38], [39], [40], [41], [42], [43], [44], [45]] (Supplemental Table 4), 13 cancer outcomes [14,32,[46], [47], [48], [49], [50], [51], [52], [53], [54], [55], [56], [57], [58], [59], [60], [61], [62], [63], [64], [65], [66]] (Supplemental Table 5), 3 metabolic disease outcomes [15,[67], [68], [69], [70], [71], [72]] (Supplemental Table 6), and 3 aging-related outcomes [16,17,[73], [74], [75]] (Supplemental Table 7). A total of 124 articles of original studies were extracted from previous meta-analyses, which combined with 63 newly added primary articles, resulting in 187 original articles. After excluding 25 articles with overlapping study populations or without absolute intake as exposures (Supplemental Table 8), we included 162 original articles, such as 17 single articles about other health outcomes (Supplemental Table 9). A total of 184 prospective observational studies from the 145 articles were applied for 35 updated meta-analyses and 4 de novo meta-analyses on 39 health outcomes. Combined with 8 health outcomes from previous meta-analyses, we obtained 47 unique health outcomes in our umbrella review (Figures 2 and 3 and Supplemental Tables 10 and 11). For the updated and de novo meta-analyses, most of the included cohorts were conducted in North America and Europe (Supplemental Figure 1)**.** No randomized controlled trials were eligible in this analysis; thus, all results were from prospective observational studies.

### Methodological quality

The evaluation of methodological quality for previous meta-analyses using AMSTAR-2 is summarized in Supplemental Table 12. A total of 11 meta-analyses [12,37,39,43,53,59,65,68,[73], [74], [75]] were of high quality, 12 meta-analyses [28,[30], [31], [32], [33],^40^,^47^,^55^,^57^,^67^,^70^,^76^] of moderate quality, 13 meta-analyses [16,29,34,36,38,41,42,44,49,51,54,56,69] of low quality, and the remaining 14 meta-analyses [[13], [14], [15],^17^,^35^,^45^,^46^,^48^,^50^,^52^,^58^,^60^,^71^,^72^] of critically low quality. Four pooled analyses [[61], [62], [63], [64]] were not suitable for the evaluation.

### All-cause mortality

**FIGURE 4:**
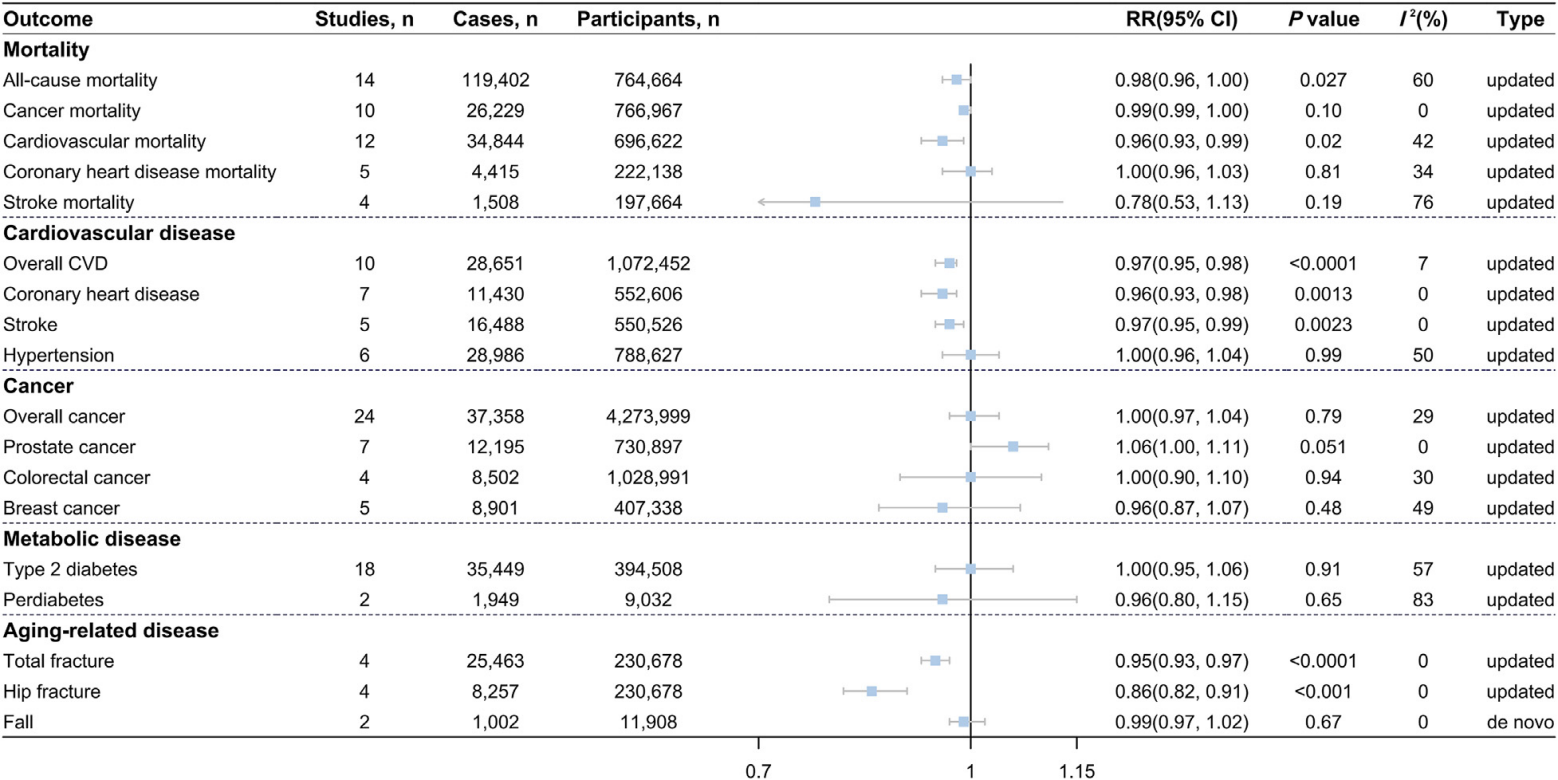
Association between cheese consumption (per 30-g/d intake level) and mortality and multiple disease incidence.

**FIGURE 5:**
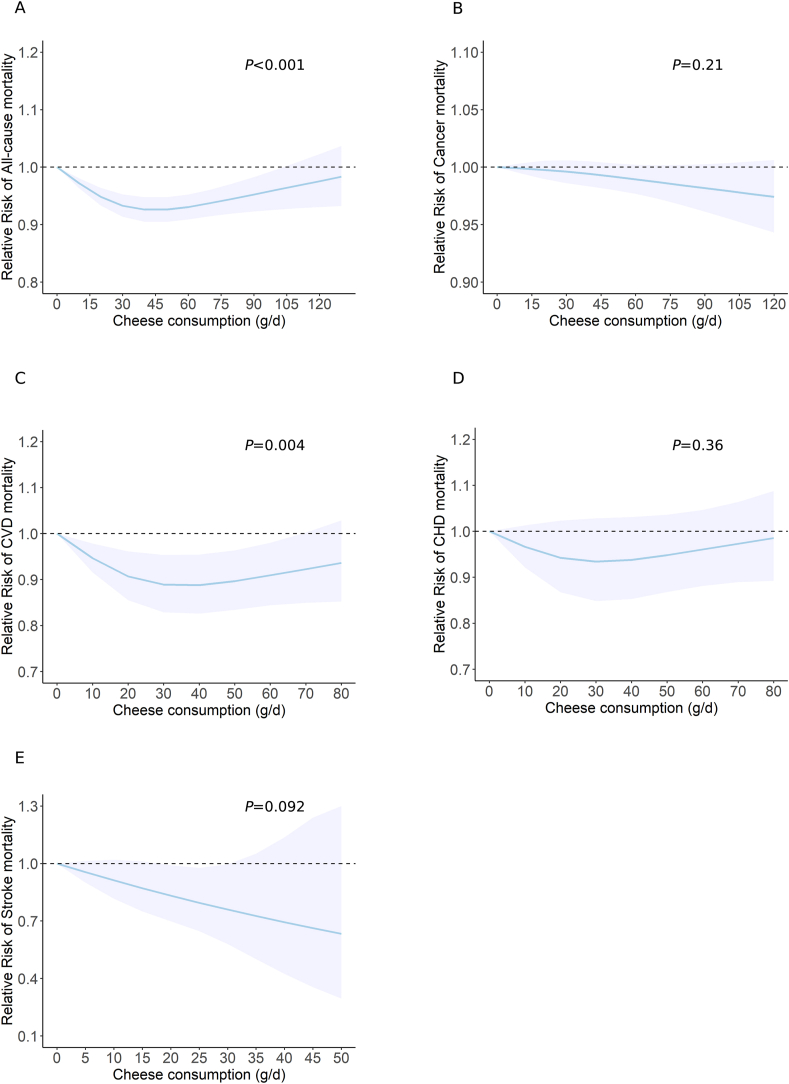
Dose–response association between cheese consumption and the risk of (A) all-cause mortality, (B) cancer mortality, (C) CVD mortality, (D) CHD mortality, and (E) stroke mortality. CHD, coronary heart disease; CVD, cardiovascular disease. P value was from the test of nonlinearity.

The updated meta-analysis of 21 studies [[77], [78], [79], [80], [81], [82], [83], [84], [85], [86], [87], [88], [89], [90], [91], [92], [93], [94]], including 136,298 cases among >1,030,243 participants, revealed an inverse association between cheese consumption and all-cause mortality (highest compared with lowest: RR = 0.95; 95% CI: 0.92, 0.99; *P* = 0.0052; *I*^2^ = 34%) (Figure 2 and Supplemental Figure 2). In the dose–response meta-analysis of 14 studies [77,79,[81], [82], [83], [84], [85],[87], [88], [89], [90], [91]], each 30-g/d cheese increment was associated with a 2% lower risk of all-cause mortality (RR = 0.98; 95% CI: 0.96, 1.00; *P* = 0.027; *I*^2^ = 60%) (Figure 4 and Supplemental Figure 3). Nonlinear analyses further showed a U-shaped association, where the lowest risk of all-cause mortality was observed at ∼40 g/d of cheese consumption (*P*-nonlinearity < 0.001) (Figure 5A).

### Cardiovascular mortality

The updated meta-analysis of 16 studies [77,78,81,82,84,85,87,88,90,91,[93], [94], [95]] encompassing 36,965 cases among 742,571 participants showed an inverse association between cheese consumption and cardiovascular mortality (highest compared with lowest: RR = 0.93; 95% CI: 0.88, 0.99; *P* = 0.015; *I*^2^ = 34%) (Figure 2 and Supplemental Figure 4). In addition, we observed a significant inverse association when cheese consumption was expressed as per 30-g/d increment in 12 studies [77,81,82,84,85,87,88,90,91,95] (RR = 0.96; 95% CI: 0.93, 0.99; *P* = 0.02; *I*^2^ = 42%) (Figure 4 and Supplemental Figure 5), the nonlinear dose–response meta-analysis suggested that the association between cheese consumption and cardiovascular mortality followed a U shape with the minimal risk at ∼35 g/d (*P*-nonlinearity = 0.004) (Figure 5C). No association was detected between cheese consumption and CHD mortality or stroke mortality (Figures 2 and 4 and Supplemental Figures 6–9).

### Cancer mortality

The updated meta-analysis of 25 studies [78,[81], [82], [83],^85^,^87^,^88^,[96], [97], [98], [99], [100], [101], [102], [103], [104], [105], [106], [107], [108]] with 30,818 cases among 1,378,932 participants found no association between cheese consumption and overall cancer mortality (highest compared with lowest: RR = 1.00; 95% CI: 0.97, 1.03; *P* = 0.91; *I*^2^ = 1%) (Figure 2 and Supplemental Figure 10). The linear and nonlinear dose–response meta-analyses yielded similar conclusions (FIGURE 4, FIGURE 5B and Supplemental Figure 11). In addition, cheese consumption was not associated with the risk of site-specific cancer mortality (Figure 2 and Supplemental Figure 12).

### Cardiovascular disease

**FIGURE 6:**
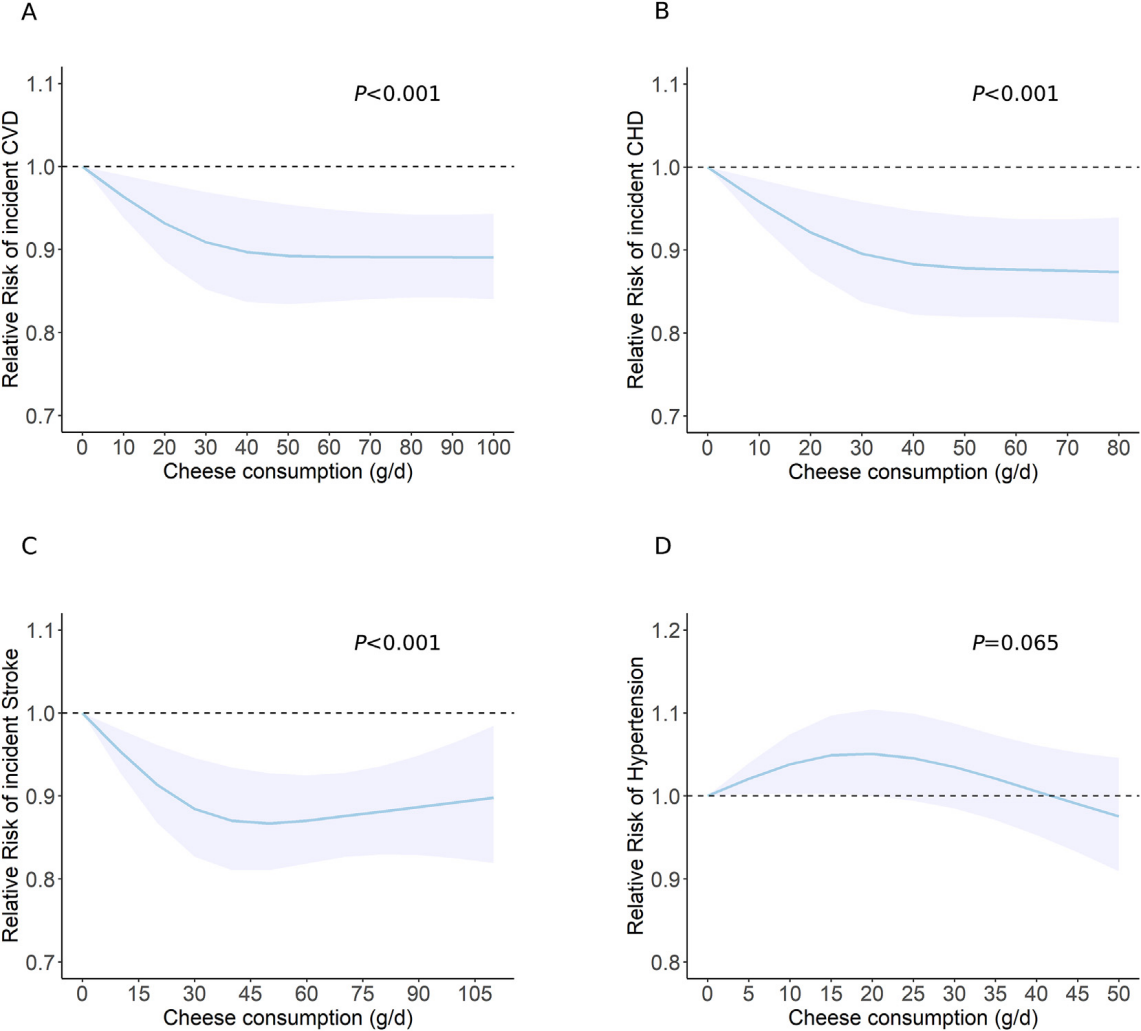
Dose–response association between cheese consumption and the risk of (A) CVD, (B) CHD, (C) stroke, and (D) hypertension. CHD, coronary heart disease; CVD, cardiovascular disease. *P* value was from the test of nonlinearity.

The updated meta-analyses of the highest compared with the lowest category of cheese consumption included 18 studies (86,796 cases among 1,833,112 participants) for overall CVD risk [80,89,[109], [110], [111], [112], [113], [114], [115], [116], [117], [118], [119], [120], [121], [122]], 12 studies (17,568 cases among 686,573 participants) for CHD risk [89,109,[112], [113], [114], [115], [116], [117],^119^,^120^], 9 studies (21,138 cases among 813,636 participants) for stroke risk [80,111,112,115,118,119,121,122], and 11 studies (>103,698 cases among 756,385 participants) for hypertension risk [110,[123], [124], [125], [126], [127], [128], [129], [130], [131]], respectively. Higher cheese consumption was associated with reduced risk of overall CVD (RR = 0.92; 95% CI: 0.89, 0.96; *P* = 0.0001; *I*^2^ = 38%) (Figure 3 and Supplemental Figure 13), CHD (RR = 0.92; 95% CI: 0.86, 0.98; *P* = 0.0108; *I*^2^ = 27%) (Figure 3 and Supplemental Figure 15), and stroke (RR = 0.93; 95% CI: 0.89, 0.98; *P* = 0.003; *I*^2^ = 0%) (Figure 3 and Supplemental Figure 17). Each 30 g/d cheese intake increment was associated with approximately 3% lower risk of overall CVD (RR = 0.97; 95%CI: 0.95, 0.98; *P*<0.0001) (Figure 4 and Supplemental Figure 14), CHD (RR = 0.96; 95%CI: 0.93, 0.98; *P* = 0.0013) (Figure 4 and Supplemental Figure 16), and stroke (RR = 0.97; 95%CI: 0.95, 0.99; *P* = 0.0023) (Figure 4 and Supplemental Figure 18). The nonlinear dose–response meta-analysis demonstrated an L-shaped association of cheese consumption with the risk of overall CVD, CHD, and stroke, leveling off at ∼40 g/d (all *P*-nonlinearity<0.001) (Figure 6A–C). Cheese consumption was not associated with hypertension risk (FIGURE 3, FIGURE 4, FIGURE 6D and Supplemental Figures 19 and 20).

### Cancer

Forty-six studies [[61], [62], [63], [64],^66^,^98^,[132], [133], [134], [135], [136], [137], [138], [139], [140], [141], [142], [143], [144], [145], [146], [147], [148], [149], [150], [151], [152], [153], [154], [155], [156], [157], [158], [159], [160], [161], [162], [163], [164], [165], [166], [167], [168], [169]] comprising 158,601 cases and 9,539,703 participants were included in the updated meta-analysis for overall cancer risk in the highest compared with lowest cheese intake comparison, yielding a nonsignificant summary RR (RR = 0.99; 95% CI: 0.97, 1.01; *P* = 0.29; *I*^2^ = 21%) (Figure 3 and Supplemental Figure 21). Meta-analyses based on 24 studies [61,66,132,136, [139], [140], [141], 143,146,149,[152], [153], [154], [155], [156], [157], [158], [159], [160], [161], [162], [163], [164]] also showed that each 30-g/d increment in cheese intake was not related to overall cancer risk (RR = 1.00, 95% CI: 0.97, 1.04; *P* = 0.79; *I*^2^ = 29%) (Figure 4 and Supplemental Figure 22). Concerning the risk of site-specific cancer and its subtypes, cheese consumption was not associated with the risk of total and advanced prostate cancer (Figure 3 and Supplemental Figure 23); colorectal cancer (FIGURE 3, FIGURE 4 and Supplemental Figures 25 and 26); total, proximal and distal colon cancer, and rectal cancer (Figure 3 and Supplemental Figure 27); total and estrogen receptor-positive (ER+) breast cancer (FIGURE 3, FIGURE 4 and Supplemental Figures 28 and 29); and other cancers (Figure 3 and Supplemental Figure 30) but was inversely associated with estrogen receptor-negative (ER−) breast cancer (RR = 0.89, 95% CI: 0.82, 0.97; *P* = 0.01; *I*^2^ = 0%) (Figure 3 and Supplemental Figure 28C). Linear dose–response meta-analysis of 7 studies suggested a marginally positive association between cheese consumption and prostate cancer risk (per 30-g/d increase: RR = 1.06; 95% CI: 1.00, 1.11; *P* = 0.051; *I*^2^ = 0%) (Figure 4 and Supplemental Figure 24). No nonlinear associations were observed between cheese consumption and the risk of overall cancer, prostate cancer, colorectal cancer, and breast cancer (Supplemental Figure 42A–D). In addition, a previous meta-analysis of 2 studies reported that cheese consumption was not associated with the risk of precursors of colorectal cancer (Figure 3).

### Metabolic disease

Our updated meta-analysis of 25 studies [80,89,95,112,[170], [171], [172], [173], [174], [175], [176], [177], [178], [179], [180], [181], [182], [183], [184], [185], [186]] enrolling 674,107 participants and 44,584 cases observed that cheese consumption was inversely associated with type 2 diabetes (T2D) risk (RR = 0.93; 95% CI: 0.88, 0.98, *P* = 0.009; *I*^2^ = 45%) (Figure 3 and Supplemental Figure 31) when comparing the highest with lowest levels of intake. However, the dose–response meta-analyses did not support the inverse association between cheese consumption and T2D risk (Figure 4 and Supplemental Figures 32 and 43A). The de novo meta-analysis of 3 studies [[186], [187], [188]] with 2,851 cases among 10,899 participants showed no association of cheese consumption with prediabetes risk (highest compared with lowest: RR = 0.93; 95% CI: 0.78, 1.12; *P* = 0.45; *I*^2^ = 66%) (FIGURE 3, FIGURE 4 and Supplemental Figures 33 and 34). Similarly, cheese consumption was not associated with metabolic syndrome risk in the updated meta-analysis of 4 studies [[123], [181], [189], [190]] (Figure 3 and Supplemental Figure 35A) and overweight/obesity risk in the meta-analysis of 2 studies [191,192] (Figure 3 and Supplemental Figure 35B).

### Aging-related disease

The updated meta-analysis of 7 studies [88,[193], [194], [195], [196]] for cheese consumption (the highest compared with lowest intake) and total fracture risk yielded a summary RR of 0.90 (95% CI: 0.86, 0.95; *P* < 0.0001; *I*^2^ = 0%) (Figure 3 and Supplemental Figure 36). The meta-analysis of 4 studies showed that the risk of total fracture decreased by 5% for each 30-g/d cheese increment (RR = 0.95, 95% CI: 0.93, 0.97; *P* < 0.0001; *I*^2^ = 0%) (Figure 4 and Supplemental Figure 37). The dose–response meta-analysis showed an L-shaped association between cheese consumption and total fracture risk, where total fracture risk decreased sharply until ∼40 g/d (*P*-nonlinearity < 0.0001) (Supplemental Figure 43B). Null association was found between cheese consumption and hip fracture risk in the updated meta-analysis of 7 studies [88,[193], [194], [195], [196]] (RR = 0.86; 95% CI: 0.72, 1.04; *P* = 0.11; *I*^2^ = 71%) (Figure 3 and Supplemental Figure 38) for the highest compared with lowest intake level. However, a meta-analysis of 4 studies showed that the risk of hip fracture decreased by 14% for each 30-g/d cheese increment (RR = 0.86, 95% CI: 0.82, 0.91; *P* < 0.001; *I*^2^ = 0%) (Figure 4 and Supplemental Figure 39). Meanwhile, the dose–response meta-analysis revealed an L-shaped association between cheese consumption and hip fracture risk, where hip fracture risk decreased with cheese consumption and reached a plateau at ∼40 g/d (*P*-nonlinearity < 0.001) (Supplemental Figure 43C). The de novo meta-analysis showed no association of cheese consumption with the risk of fall (FIGURE 3, FIGURE 4 and Supplemental Figures 40A and 41) and frailty (Figure 3 and Supplemental Figure 40B). Moreover, the de novo meta-analysis of 2 studies [197,198] observed that dementia risk was 19% lower for the highest than that for lowest cheese intake (RR = 0.81; 95% CI: 0.66, 0.99; *P* = 0.04; *I*^2^ = 0%) (Figure 3 and Supplemental Figure 40C). In addition, cheese consumption was marginally related to an increased risk of Parkinson disease based on a previous meta-analysis of 5 studies (RR = 1.26; 95% CI: 0.99, 1.60; *P* = 0.052; *I*^2^ = 29%) (Figure 3).

### Other health outcomes

Seventeen original articles [[199], [200], [201], [202], [203], [204], [205], [206], [207], [208], [209], [210], [211], [212], [213], [214], [215]] reported the prospective association between cheese consumption and 26 other health outcomes (Supplemental Table 9). Among them, inverse associations were found for the risk of childhood dental caries [201] (OR = 0.37; 95%CI: 0.17, 0.76), osteoporosis [205] (OR = 0.28; 95%CI: 0.12, 0.66), wheezing in infants [209] (OR = 0.51; 95%CI: 0.31, 0.85), type 1 diabetes [215] (HR = 0.55; 95%CI: 0.33, 0.94), and infant colic [210] (OR = 0.89; 95%CI: 0.79, 0.99).

### Subgroup analyses

Of the 184 original studies included in the meta-analyses, 149 (81.0%) studies adjusted for total energy intake in the models, and 152 (82.6%) studies were conducted in North America, Europe, and Oceania. Among the 47 major outcomes in our study, 4 outcomes were solely based on studies without energy adjustment, 18 outcomes were only based on studies with energy adjustment, and 30 outcomes were entirely based on studies conducted in Europe, North America, and Oceania (Supplemental Tables 13–16).

Subgroup analyses indicated that the association between cheese consumption and most health outcomes remained consistent regardless of adjustment for total energy intake or geographic location (Supplemental Tables 13–16). However, heterogeneity existed in subgroups by total energy adjustment for overall and breast cancer incidence, where higher cheese consumption was linked with an increased risk of overall cancer (RR = 1.14; 95% CI: 1.01, 1.29; *P* = 0.0305; *I*^2^ = 0%; *P*-subgroup = 0.02) (Supplemental Table 14) and was marginally associated with elevated risk of breast cancer (RR = 1.43; 95% CI:0.99, 2.06; *P* = 0.0557; *P*-subgroup = 0.04) (Supplemental Table 14) in studies without adjustment for total energy intake, whereas null associations were found in studies with adjustment for total energy intake. Although heterogeneity was observed in subgroup analyses by energy adjustment for metabolic syndrome (*P*-subgroup = 0.03), the null association was consistent between subgroups. Meanwhile, an inverse association was detected for CHD mortality (RR: 0.67; 95% CI: 0.51, 0.89; *P*-subgroup = 0.03) (Supplemental Table 15) in studies conducted in Asia and other regions but not in studies conducted in North America, Europe, and Oceania.

### Evidence credibility

The credibility of the identified associations with cheese consumption is summarized in Supplemental Tables 10 and 11, and the detailed NutriGrade scores for each meta-analysis are presented in Supplemental Table 17. No health outcome met the standards for high meta-evidence. Eight health outcomes (17%)—death from any cause, cancer, and CVD and incidence of overall CVD, CHD, stroke, hypertension, and prostate cancer—presented moderate meta-evidence. Twenty-two health outcomes (47%)—site-specific cancer mortality (the colorectum, colon, rectum, lung, and stomach), CHD mortality, overall and site-specific cancer (colorectum, total and distal colon, rectum, total, ER+ and ER− breast, bladder, and pancreas), T2D, overweight/obesity, total and hip fractures, fall, and dementia—presented low meta-evidence. The rest health outcomes indicated very low meta-evidence.

## Discussion

This umbrella review provides a broad overview of the existing evidence on the association between cheese consumption and 47 unique outcomes through 35 updated, 4 de novo, and 8 previous meta-analyses based on 184 prospective observational studies from 145 primary articles. Moderate quality of evidence showed that cheese consumption was associated with reduced risk of all-cause mortality, CVD mortality, and incident CVD, CHD, and stroke but not related to the risk of cancer mortality, hypertension, and prostate cancer. Low quality of evidence was observed for inverse associations of cheese intake with incidence of ER− breast cancer, T2D, total fracture, and dementia and null association with site-specific cancer mortality (i.e., colorectum, colon, rectum, lung, and stomach), CHD mortality, and incidence of overall, site-specific cancer and its subtypes (i.e., colorectum, total and distal colon, rectum, total and ER+ breast, bladder, and pancreas), overweight/obesity, hip fracture, and fall. The nonlinear dose–response analyses additionally suggested a U-shaped association between cheese consumption and the risk of all-cause mortality and cardiovascular mortality and an L-shaped association with the risk of overall CVD, CHD, stroke, and total and hip fractures with the optimal intake at ∼40 g/d.

Although cheese is theorized to have detrimental effects on blood pressure and blood lipid profile based on its high sodium and saturated fat contents, a moderate quality of evidence suggest that cheese consumption does not increase the risk of cardiovascular diseases and may even have protective associations with overall CVD, CHD, and stroke incidence and cardiovascular and all-cause mortality in our updated meta-analyses. The inverse associations are in line with findings from most previous meta-analyses [13,36,37,39,40,42]. In terms of the nonlinear analysis, 1 meta-analysis in 2016 reported an L-shaped association with the risk of stroke leveling off at 25 g/d of cheese intake [41]. Still, another meta-analysis in 2017 derived a U-shaped association with the lowest CVD risk at 40 g/d, which was consistent with the findings from our study [36]. Regarding cheese intake and hypertension, a null association with moderate quality of evidence was observed in our study, which was in line with previous studies [[43], [44], [45]]. In subgroup analyses, an inverse association between cheese consumption and CHD mortality was only notable in Asian populations but not in European and American populations, which may be attributable to the differences in the amount and patterns of cheese consumption among different regions [43].

Results from pervious meta-analyses [31,57,59,60,216] and large prospective cohort studies [217,218] have raised the concern regarding high consumption of dairy products (particularly whole milk) increasing the incidence and mortality of several cancers, for example, prostate, breast, ovarian, and liver cancers and lymphoma. By contrast, cheese consumption has been reported to be inversely related to the risk of colorectal cancer, breast cancer, and prostate cancer in earlier meta-analyses including both prospective studies and case–control studies [46,51,58]. However, our latest meta-analyses of prospective observational studies found null associations between cheese consumption and overall and site-specific cancer incidence and mortality, consistent with previous meta-analyses for overall cancer incidence and mortality [14,30,31] and colorectal cancer [32,53]. Of note, total energy intake is a crucial confounder in the association between cheese consumption and cancer risk. Failure to adjust for total energy intake in the analyses could lead to spurious conclusions, such as in the case that higher cheese consumption was associated with an elevated risk of overall and breast cancer when not controlling total energy intake. The quality of evidence was moderate for null associations with total cancer mortality and incident prostate cancer and low for null associations with mortality from colorectal, colon, rectal, lung, and gastric cancers and incidence of overall, colorectal, colon (total and distal), rectal, breast (total and ER+), bladder, and pancreatic cancers. In addition, low-quality evidence revealed an inverse association between cheese intake and ER− breast cancer incidence, which was driven by a protective association of cottage/ricotta cheese consumption rather than hard cheese consumption with ER− breast cancer risk in a pooled analysis of 21 cohort studies [62]. The findings for prostate cancer incidence are also warranted to be confirmed in large-scale, long-term, prospective cohort studies because our linear dose–response meta-analysis of 7 cohort studies suggested a borderline positive association between cheese consumption and prostate cancer risk.

We found a low quality of evidence for an inverse association between cheese consumption and T2D risk in the highest compared with that of the lowest intake, which is in accordance with previous meta-analyses [67,68]. Substitution analysis demonstrated that replacing red and processed meat (per 50 g/d) with cheese (per 30 g/d) was associated with 10% decreased risk of T2D [219]. However, our linear and nonlinear dose–response analyses did not find significant associations, which was consistent with the most recent dose–response meta-analysis [220]. Inconsistently, a meta-analysis published in 2013 showed that per 50-g cheese/d was associated with 8% reduced risk of T2D, and there was a marginal nonlinear association between cheese consumption and T2D risk, with a reduction ≤50 g/d [15]. More studies are needed to clarify the discrepancy between categorized and dose–response analyses.

Dairy products are rich in calcium, magnesium, phosphorus, and protein, which are essential for good bone health [221,222]. Nevertheless, the role of dairy intake in preventing bone fractures remains debated [223]. Previous meta-analyses reported both inverse and null associations between cheese intake and the risk of hip fracture [[73], [74], [75]] and fracture at any site [73]. Our updated meta-analyses including only prospective studies supported a favorable association of cheese intake with total fracture risk in the highest compared with that of the lowest intake and with hip fracture risk per 30-g/d increase in cheese consumption. Nonlinear dose–response analyses showed an L-shaped association between cheese consumption and total and hip fracture risk, leveling off at ∼40 g/d. Given that the quality of evidence was low, further research is warranted to confirm these findings.

Additionally, low quality of evidence also showed that higher cheese intake was associated with a lower dementia risk in our de novo meta-analysis of 2 prospective cohort studies [197,198]. The beneficial association was supported by previous randomized controlled crossover trial [224] and observational studies [225,226], suggesting that cheese consumption may improve cognitive function.

The protective association of cheese consumption with mortality, CVD, bone fracture, and dementia may be attributed to the abundance of nutrients, bioactive compounds, and probiotics in cheese. Dairy products, especially cheese, are a predominant dietary source of vitamin K_2_ in many regions [227,228]. Vitamin K_2_ can improve cardiovascular health by inhibiting and reversing vascular calcification [229,230], reduce age-related bone loss through promoting the γ-carboxylation of osteocalcin and increasing osteoprotegerin [230], and maintain neurocognitive functions through contributing to the biological activation of proteins Gas6 and protein S and the synthesis of sphingolipids [231]. Probiotic bacteria in cheese may also interact with the gut microbiome [232], exerting various health enhancing functions [233]. Additionally, the cheese matrix can mitigate the harmful effects of saturated fat and sodium [[234], [235], [236], [237]]. Besides the components of cheese itself (i.e., protein or specific micronutrients), the observed inverse associations could also be owing to the fact that increased cheese intake may replace consumption of other foods (e.g., processed/red meat and refined carbohydrates) that have been consistently associated with higher risk of incidence or mortality from chronic diseases [[238], [239], [240]] because the studies adjusting for total energy intake hold calories constant, as in isocaloric intervention trials.

It is noteworthy that a borderline positive association between cheese intake and Parkinson disease risk was observed in a previous meta-analysis of 5 cohort studies, which accords with findings from the latest meta-analysis on total dairy and milk [241] and 1 recent prospective study on low-fat dairy foods (including cottage cheese and low-fat cheese) [242]. If causal, suggested mechanisms include reduction on uric acid by dairy proteins and the inhibition of calcium and phosphate in dairy products on the formation of 1,25(OH)_2_D_3_ (1,25-dihydroxy-vitamin D_3_ = calcitriol) because urate and 1,25(OH)_2_D_3_ may protect against Parkinson’s disease [242,243]. However, the quality of the meta-evidence was very low, and inconsistent results were observed for cheese and other fermented dairy products in some prospective studies [244,245]. Accordingly, the findings should be interpreted with caution and validated by further studies.

### Strengths and limitations

This umbrella review provides the most recent evidence from prospective observational studies on the association between cheese intake and a wide range of health outcomes. Different from traditional umbrella reviews focusing only on published meta-analyses, we thoroughly and systematically resynthesized the available evidence by incorporating newly identified prospective studies into prospective studies included in previous meta-analyses. On one hand, we updated those outdated meta-analyses to reflect up-to-date conclusions with more statistical power. On the other hand, we performed de novo meta-analyses for specific health outcomes without previous meta-analyses despite enough published original studies to include as many potentially related health outcomes as possible. Furthermore, dose–response analyses were conducted to reveal further the linear and nonlinear association between cheese intake and multiple health outcomes, thereby determining the optimal consumption level of cheese.

There are also some limitations in our research. Given that the original studies included in this review are all observational, some of their inherent limitations could not be excluded, such as residual confounding and reverse causality. Besides, the updated meta-analyses for some health outcomes are highly heterogeneous (*I*^2^ ≥ 50%), probably due to the inclusion of original studies involving different populations. Also, caution should be taken when generalizing the conclusions to populations with different genetic backgrounds and dietary habits because most primary studies included were conducted in Europe and North America. Moreover, different types of cheese also vary a lot in dairy matrix and nutrient content like fat and sodium, which may deliver divergent health effects. However, the lack of information on cheese type deterred finer stratified analyses by cheese type. Finally, the limited number of prospective observational studies in meta-analyses for cheese consumption and specific health outcomes—such as cancer at sites other than colorectum, breast, and prostate, overweight/obesity, dementia, fall, and frailty—leads to insufficient statistical power and low credibility of evidence. Thus, further large-scale prospective studies are warranted to ascertain the association of cheese intake with these health outcomes.

## Conclusions

Our results indicate that cheese consumption has neutral to moderate benefits for human health, particularly ≥40 g/d, with a moderate quality of evidence for inverse associations with all-cause and CVD mortality and overall CVD, CHD, and stroke incidences. Null associations were observed with cancer mortality, hypertension, and prostate cancer incidence. Although high saturated fat and sodium in some cheeses tend to be emphasized as a health concern in dietary guidelines, cheese also provides some nutrients and bioactive compounds, which potentially may confer some benefits. Environmental effects of cheese production should also be considered.

### Author contribution

The authors’ responsibilities were as follows—AF, YW: conceived the study; AF: designed the study; MZ, XD, ZH: performed the literature search and screening and extracted the data; MZ, XD: conducted the data analyses and drafted the manuscript; AF: revised the manuscript; ELG, XL: critically reviewed the manuscript; AF, ELG: had primary responsibility for final content; and all authors: contributed substantially to the interpretation of the data and read and approved the final manuscript.

### Conflicts of interest

The authors report no conflicts of interest.

### Data availability

All data included in this umbrella review were extracted from publicly available systematic reviews and original studies.

## Appendix A Supplementary data

Multimedia component1 Multimedia component1

Multimedia component10 Multimedia component10

Multimedia component11 Multimedia component11

Multimedia component12 Multimedia component12

Multimedia component13 Multimedia component13

Multimedia component2 Multimedia component2

Multimedia component3 Multimedia component3

Multimedia component4 Multimedia component4

Multimedia component5 Multimedia component5

Multimedia component6 Multimedia component6

Multimedia component7 Multimedia component7

Multimedia component8 Multimedia component8

Multimedia component9 Multimedia component9
