# Supplementary material for: Cheese consumption and multiple health outcomes: an umbrella review and updated meta-analysis of prospective studies
Source: Adv Nutr. 2023 Jun 15;14(5):1170–86. doi: 10.1016/j.advnut.2023.06.007 (PMC10509445; doi:10.1016/j.advnut.2023.06.007)
Supplement: Multimedia component1 [file mmc1.docx]

**Cheese consumption and multiple health outcomes: an umbrella review and updated meta-analysis of prospective studies**

Mingjie Zhang, Xiaocong Dong, Zihui Huang, Xue Li, Yue Zhao, Yingyao Wang, Huilian Zhu, Aiping Fang, Edward L. Giovannucci

**List of Supplementary Tables**

[Supplementary Table 1. Search strategy for systematic reviews and meta-analyses on cheese consumption and health outcomes. 3](#_Toc134632278)

[Supplementary Table 2. Search strategy for original articles on cheese consumption and health outcomes. 4](#_Toc134632279)

[Supplementary Table 3. Description of meta-analyses on cheese consumption and all-cause and cause-specific mortality included in the umbrella review. 6](#_Toc134632280)

[Supplementary Table 4. Description of meta-analyses on cheese consumption and cardiovascular disease risk included in the umbrella review. 8](#_Toc134632281)

[Supplementary Table 5. Description of meta-analyses/pooled analyses on cheese consumption and cancer risk included in the umbrella review. 11](#_Toc134632282)

[Supplementary Table 6. Description of meta-analyses on cheese consumption and metabolic disease risk included in the umbrella review. 16](#_Toc134632283)

[Supplementary Table 7. Description of meta-analyses on cheese consumption and aging-related disease risk included in the umbrella review. 18](#_Toc134632284)

[Supplementary Table 8. List of studies excluded from updated meta-analysis and reasons for exclusion. 19](#_Toc134632285)

[Supplementary Table 9. Description of original studies on the association between cheese consumption and other health outcomes. 21](#_Toc134632286)

[Supplementary Table 10. Association between cheese consumption (highest vs. lowest intake level) and all-cause and cause-specific mortality. 25](#_Toc134632287)

[Supplementary Table 11. Association between cheese consumption (highest vs. lowest intake level) and disease risk. 27](#_Toc134632288)

[Supplementary Table 12. The detailed assessments of methodological quality of the included systematic reviews and meta-analyses. 30](#_Toc134632289)

[Supplementary Table 13. Subgroup analyses of the association between cheese consumption (highest vs. lowest intake level) and all-cause and cause-specific mortality according to whether adjusting for total energy intake in the models 35](#_Toc134632290)

[Supplementary Table 14. Subgroup analyses of the association between cheese consumption (highest vs. lowest intake level) and disease risk according to whether adjusting for total energy intake in the models 37](#_Toc134632291)

[Supplementary Table 15. Subgroup analyses of the association between cheese consumption (highest vs. lowest intake level) and all-cause and cause-specific mortality according to geographic locations 41](#_Toc134632292)

[Supplementary Table 16. Subgroup analyses of the association between cheese consumption (highest vs. lowest intake level) and disease risk according to geographic locations 43](#_Toc134632293)

[Supplementary Table 17. Scoring for the different components of NutriGrade for each health outcome. 47](#_Toc134632294)

**Supplementary Table** **1. Search strategy for systematic reviews and meta-analyses on cheese consumption and health outcomes.**

| Database | Search Strategy |
| --- | --- |
| Pubmed | (cheese) AND (“meta analysis” OR meta-analysis OR “meta analyzed” OR meta-analyzed OR “pooled analysis” OR “systematic review”) |
| Embase | #1 Cheese  #2 'meta analysis' OR 'meta-analysis' OR 'meta analyzed' OR 'meta-analyzed' OR 'pooled analysis' OR 'systematic review'  #3 #1 AND #2 |
| Cochrane library | (cheese) AND (“meta analysis” OR meta-analysis OR “meta analyzed” OR meta-analyzed OR “pooled analysis” OR “systematic review”) |

**Supplementary Table 2. Search strategy for original articles on cheese consumption and health outcomes.**

| Database | Search Strategy |
| --- | --- |
| Pubmed | (("Cheese"[Mesh]) OR (cheese[Text Word])) AND (((((((((prospective*[Title/Abstract]) OR (cohort*[Title/Abstract])) OR (longitudinal[Title/Abstract])) OR (follow-up[Title/Abstract])) OR (case-cohort[Title/Abstract])) OR ("nested case-control"[Title/Abstract])) OR ((("clinical trial"[Publication Type]) OR ("controlled clinical trial"[Publication Type])) OR ("randomized controlled trial"[Publication Type]))) OR ((((("Clinical Trials as Topic"[Mesh]) OR "Longitudinal Studies"[Mesh]) OR "Prospective Studies"[Mesh]) OR "Cohort Studies"[Mesh]) OR "Follow-Up Studies"[Mesh])) OR (((randomized[Title/Abstract]) OR (randomised[Title/Abstract])) AND ((trial[Title/Abstract]) OR (intervention[Title/Abstract])))) Filters: from 1000/1/1 - 2022/2/10 Sort by: Most Recent |
| Embase | #1 'cheese'/exp  #2 cheese:ti,ab,kw  #3 #1 OR #2  #4 'longitudinal study'/exp  #5 'prospective study'/exp  #6 'cohort analysis'/exp  #7 'follow up'/exp  #8 'randomized controlled trial'/exp  #9 prospective*:ti,ab,kw  #10 cohort*:ti,ab,kw  #11 longitudinal:ti,ab,kw  #12 'follow up':ti,ab,kw  #13'case cohort':ti,ab,kw  #14 'nested case-control':ti,ab,kw  #15 (randomized:ti,ab,kw OR randomised:ti,ab,kw) AND (trial:ti,ab,kw OR intervention:ti,ab,kw)  #16 #4 OR #5 OR #6 OR #7 OR #8  #17 #9 OR #10 OR #11 OR #12 OR #13 OR #14 OR #15  #18 #16 OR #17  #19 #3 AND #18  #20 #3 AND #18 AND [01-01-1960]/sd NOT [11-02-2022]/sd |
| Cochrane library | #1 MeSH descriptor: [Cheese] explode all trees  #2 (cheese*):ti,ab,kw (Word variations have been searched)  #3 #1 OR #2  #4 MeSH descriptor: [Longitudinal Studies] explode all trees  #5 MeSH descriptor: [Prospective Studies] explode all trees  #6 MeSH descriptor: [Cohort Studies] explode all trees  #7 MeSH descriptor: [Follow-Up Studies] explode all trees  #8 MeSH descriptor: [Randomized Controlled Trial] explode all trees  #9 #4 OR #5 OR #6 OR #7 OR #8 (Word variations have been searched)  #10 (prospective*):ti,ab,kw (Word variations have been searched)  #11 (cohort*):ti,ab,kw (Word variations have been searched)  #12 (longitudinal):ti,ab,kw (Word variations have been searched)  #13 ('follow up'):ti,ab,kw (Word variations have been searched)  #14 ('case cohort'):ti,ab,kw (Word variations have been searched)  #15 ('nested case-control'):ti,ab,kw (Word variations have been searched)  #16 (randomized OR randomised):ti,ab,kw AND (trial OR intervention):ti,ab,kw (Word variations have been searched)  #17 #10 OR #11 OR #12 OR #13 OR #14 OR #15 OR #16  #18 #9 OR #17  #19 #3 AND #18 with Cochrane Library publication date to Feb 2022, in Cochrane Protocols, Trials |

# Supplementary Table 3. Description of meta-analyses on cheese consumption and all-cause and cause-specific mortality included in the umbrella review.

| **Author, year** | **Outcome** | **Population** | **Study design** | **Comparison/Intake** | **No of studies** | **Cases/Total** | **Metric** | **Effect size**  **(95% CI)** | ***P* value** | **Egger’s *P*** | ***I*^2^**  **(%)** |
| --- | --- | --- | --- | --- | --- | --- | --- | --- | --- | --- | --- |
| O'Sullivan, 2013^1^ | All-cause mortality | general | CS | highest vs. lowest | 6 | 17,753/23,076 | RR | 1.03 (0.97, 1.09) | 0.38 | NA | 0 |
|  |  | general | CS | per 40g/w | 5 | NA | RR | 1.00 (1.00, 1.00) | 0.106 | NA | NA |
|  | CVD mortality | general | CS | highest vs. lowest | 6 | 4,777/3,3716 | RR | 1.00 (0.81, 1.24) | >0.999 | NA | 15 |
|  |  | general | CS | per 40g/w | 3 | NA | RR | 1.01 (0.99, 1.02) | 0.013 | NA | NA |
| Guo, 2017^2^ | All-cause mortality | general | CS | per 10g/d | 13 | 54,125/342,120 | RR | 0.99 (0.96, 1.01) | NA | 0.31 | 93.3 |
| Tong, 2017^3^ | All-cause mortality | general | CS | highest vs. lowest | 9 | 21,365/177,655 | RR | 1.02 (0.97, 1.06) | NA | 0.37 | 0 |
|  |  |  | CS | per 50g/d | 8 | 20,962/175,699 | RR | 1.03 (0.99, 1.07) | NA | NA | 0 |
| Lu, 2016^4^ | Cancer mortality | general | CS | highest vs. lowest | 3 | 1,569/436,121 | RR | 1.23 (0.94, 1.61) | 0.127 | NA | 0 |
|  |  | general | CS | per 1 serving/d | NA | NA | RR | 1.36 (0.90, 2.05) | NA | NA | 5.3 |
| Jin, 2021^5^ | Cancer mortality | general | CS | highest vs. lowest | 10 | 25,894/473,990 | RR | 0.99 (0.98, 1.01) | NA | NA | 0 |
|  |  | general | CS | per 50g/d | 8 | 24,823/44,635 | RR | 1.01 (0.95, 1.07) | NA | NA | 36.3 |
|  | Colorectal cancer mortality | general | CS | highest vs. lowest | 6 | >393/271,899 | RR | 1.22 (1.02, 1.46) | NA | NA | 0 |
|  | Pancreatic cancer mortality | general | CS | highest vs. lowest | 4 | >25/220,913 | RR | 1.25 (0.89, 1.76) | NA | NA | 0 |
|  | Lung cancer mortality | general | CS | highest vs. lowest | 6 | >781/339,356 | RR | 0.85 (0.63, 1.15) | NA | NA | 41.5 |
|  | Breast cancer mortality | general | CS/NCC | highest vs. lowest | 3 | >142/169,147 | RR | 1.16 (0.88, 1.53) | NA | NA | 0 |
|  | Ovarian cancer mortality | general | CS | highest vs. lowest | 3 | >77/232,480 | RR | 1.24 (0.81, 1.90) | NA | NA | 0 |
|  | Prostate cancer mortality | general | CS | highest vs. lowest | 3 | >277/350,253 | RR | 1.17 (0.89, 1.55) | NA | NA | 0 |
|  | Urothelial cancer mortality | general | CS | highest vs. lowest | 1 | 88/114,517 | RR | 0.91 (0.68, 1.21) | NA | NA | NA |
| Jin, 2020^6^ | Colorectal cancer mortality | general | CS | highest vs. lowest | 3 | 483/136,475 | RR | 1.18 (0.95, 1.47) | NA | >0.2 | 0 |
| Sun, 2014^7^ | Gastric cancer mortality | general | CS | highest vs. lowest | 2 | 910/113,950 | RR | 1.02 (0.66, 1.58) | NA | NA | 0 |
| Yang, 2016^8^ | Lung cancer mortality | general | CS | highest vs. lowest | 2 | 730/118,094 | RR | 0.63 (0.46, 0.87) | NA | NA | 0 |

**Abbreviation：** CS=cohort study; CC= case-control study; RR= relative risk; OR= odds ratio; HR= hazard ratio; CVD= cardiovascular disease; NA= not available.

# Supplementary Table 4. Description of meta-analyses on cheese consumption and cardiovascular disease risk included in the umbrella review.

| **Author, year** | **Outcome** | **Population** | **Study design** | **Comparison/Intake** | **No of studies** | **Cases/Total** | **Metric** | **Effect size**  **(95% CI)** | ***P* value** | **Egger’s *P*** | ***I*^2^**  **(%)** |
| --- | --- | --- | --- | --- | --- | --- | --- | --- | --- | --- | --- |
| Zhang, 2020^9^ | Cardiovascular disease | general | CS | highest vs. lowest | 8 | 58,305/279,289 | OR | 0.87 (0.80, 0.94) | NA | NA | NA |
| Alexander, 2016^10^ | Cardiovascular disease | general | CS | highest vs. lowest | 3 | 3,362/62,810 | RR | 0.89 (0.78, 1.01) | NA | NA | 13 |
|  | Coronary heart disease | general | CS | highest vs. lowest | 5 | 5,108/194,911 | RR | 0.82 (0.72, 0.93) | NA | NA | 0 |
|  | Stroke | general | CS | highest vs. lowest | 4 | 8,455/256,778 | RR | 0.87 (0.77, 0.99) | NA | NA | 33.5 |
| Chen, 2017^11^ | Cardiovascular disease | general | CS | highest vs. lowest | 7 | 7,022/102,013 | RR | 0.90 (0.82, 0.99) | NA | >0.10 | 0 |
|  |  | general | CS | per 50g/d | 7 | 7,022/102,013 | RR | 0.92 (0.83, 1.02) | NA | NA | 16.9 |
|  | Coronary heart disease | general | CS | highest vs. lowest | 8 | 7,425/121,226 | RR | 0.86 (0.77, 0.96) | NA | 0.04 | 14.9 |
|  |  | general | CS | per 50g/d | 8 | 7,425/121,226 | RR | 0.90 (0.84, 0.95) | NA | NA | 0 |
|  | Stroke | general | CS | highest vs. lowest | 6 | 10,449/257,069 | RR | 0.90 (0.84, 0.97) | NA | >0.10 | 0 |
|  |  | general | CS | per 50g/d | 5 | 9,759/171,305 | RR | 0.94 (0.85, 1.04) | NA | NA | 63.7 |
| Chen, 2021^12^ | Cardiovascular disease | general | CS | highest vs. lowest | 23 | 566,96/1,073,855 | RR | 0.92 (0.88, 0.96) | NA | NA | 42.4 |
|  |  | general | CS | 1-serving/d increase | 13 | 31,683/563,889 | RR | 0.97 (0.94, 1.00) | NA | NA | 69.7 |
|  | Coronary heart disease | general | CS | highest vs. lowest | 15 | 19,940/684,832 | RR | 0.90 (0.84, 0.97) | NA | NA | 47.1 |
|  |  | general | CS | 1-serving/d increase | 7 | 7,299/139,207 | RR | 0.97 (0.94, 1.01) | NA | NA | 62.2 |
|  | Stroke | general | CS | highest vs. lowest | 10 | 14,799/335,543 | RR | 0.94 (0.88, 1.00) | NA | NA | 23.8 |
|  |  | general | CS | 1-serving/d increase | 7 | 10,986/202,562 | RR | 0.96 (0.91, 1.01) | NA | NA | 52.1 |
|  | Hypertension | general | CS | highest vs. lowest | 9 | >95,066/264,514 | RR | 0.97 (0.94, 1.01) | NA | NA | 41.8 |
|  |  | general | CS | 1-serving/d increase | 6 | 12,702/50,502 | RR | 1.00 (0.96, 1.04) | NA | NA | 54 |
| Guo, 2017^2^ | Cardiovascular disease | general | CS | per 10g/d | 11 | 15,519/234,447 | RR | 0.98 (0.95, 1.00) | NA | NA | 82.6 |
|  | Coronary heart disease | general | CS | per 10g/d | 10 | 4,022/256,091 | RR | 0.99 (0.97, 1.02) | NA | 0.273 | 40.3 |
| Gholami, 2017^13^ | Coronary heart disease | general | CS | highest vs. lowest | 10 | >8,414/228,909 | RR | 0.90 (0.81, 1.01) | NA | 0.38 | 47.4 |
|  | Stroke | general | CS | highest vs. lowest | 7 | 10,483/286,674 | RR | 0.93 (0.88, 0.99) | NA | 0.65 | 0 |
| Jakobsen, 2021^14^ | Coronary heart disease | general | CS | highest vs. lowest | 9 | 14,698/554,323 | RR | 0.91 (0.84, 0.99) | NA | NA | 37 |
|  |  | general | CS | highest vs. lowest  (Low-fat cheese) | 4 | 5,081/133,967 | RR | 1.17 (0.85, 1.61) | NA | NA | 85 |
|  |  | general | CS | highest vs. lowest  (High-fat cheese) | 2 | 5,065/132,208 | RR | 0.94 (0.77, 1.14) | NA | NA | 71 |
|  |  | general | CS | per 20g/d | 8 | 1,0403/455,751 | RR | 0.96 (0.93, 0.98) | NA | NA | 3 |
|  |  | general | CS | per 20g/d  (Low-fat cheese) | 3 | 1,843/35,395 | RR | 1.24 (0.76, 2.04) | NA | NA | 90 |
|  | Ischemic stroke | general | CS | highest vs. lowest | 3 | 6,208/187,281 | RR | 0.89 (0.78, 1.01) | NA | NA | 37 |
|  |  | general | CS | per 20g/d | 3 | 6,208/187,281 | RR | 0.96 (0.91, 1.01) | NA | NA | 67 |
| Qin, 2015^15^ | Coronary heart disease | general | CS | highest vs. lowest | 7 | 6,263/197,749 | RR | 0.84 (0.71, 1.00) | NA | NA | 31.8 |
|  | Stroke | general | CS | highest vs. lowest | 4 | 8,212/222,369 | RR | 0.91 (0.84, 0.98) | NA | NA | 0 |
| de Goede, 2016^16^ | Stroke | general | CS | per 40g/d | 8 | 11,126/272,368 | RR | 0.97 (0.94, 1.01) | 0.12 | NA | 31.2 |
| Hu, 2014^17^ | Stroke | general | CS | highest vs. lowest | 6 | 9,919/282,439 | RR | 0.94 (0.89, 0.995) | 0.03 | NA | 0 |
| Heidari, 2021^18^ | Hypertension | general | CS | highest vs. lowest | 11 | >103,109/291,472 | RR | 0.97 (0.92, 1.01) | NA | 0.704 | 53 |
| Soedamah-Muthu, 2012^19^ | Hypertension | general | CS | per 30g/d | 8 | 15,066/51,007 | RR | 1.00 (0.98, 1.03) | NA | NA | 0 |
| Ralston, 2012^20^ | Hypertension | general | CS | highest vs. lowest | 4 | 10,739/38,889 | RR | 1.00 (0.89, 1.12) | 0.99 | NA | 11 |

**Abbreviation：** CS=cohort study; CC= case-control study; RR= relative risk; OR= odds ratio; HR= hazard ratio; NA= not available.

# Supplementary Table 5. Description of meta-analyses/pooled analyses on cheese consumption and cancer risk included in the umbrella review.

| **Author, year** | **Outcome** | **Population** | **Study design** | **Comparison/Intake** | **No of studies** | **Cases/Total** | **Metric** | **Effect size**  **(95% CI)** | ***P* value** | **Egger’s *P*** | ***I*^2^**  **(%)** |
| --- | --- | --- | --- | --- | --- | --- | --- | --- | --- | --- | --- |
| Zhang, 2019^21^ | Cancer | general/cases | CS/CC | highest vs. lowest | 66 | 36,290/1,916,717 | OR | 1.02 (0.95, 1.10) | NA | NA | NA |
|  |  | general | CS | highest vs. lowest | 18 | 12,464/1,862,271 | OR | 0.91 (0.81, 1.02) | NA | NA | NA |
|  |  | general/cases | CC | highest vs. lowest | 48 | 23,826/54,446 | OR | 1.08 (0.99, 1.19) | NA | NA | NA |
| Boyd, 1993^22^ | Breast cancer | general/cases | CS/CC | highest vs. lowest | 6 | 2,521/7,452 | RR | 1.17 (1.02, 1.36) | NA | NA | NA |
| Kazemi, 2021^23^ | Breast cancer | general | CS | per 30g/d | 10 | 39,703/1,419,872 | RR | 0.95 (0.91, 0.996) | NA | NA | 75.1 |
| Wu, 2021^24^ | Breast cancer | general | CS | highest vs. lowest  (Hard cheese) | 14 | 37,580/1,141,849 | HR | 1.01 (0.93, 1.10) | NA | NA | NA |
|  |  | general | CS | highest vs. lowest  (Cottage/ricotta cheese) |  | 30,463/1,141,849 | HR | 0.97 (0.93, 1.01) | NA | NA | NA |
| Li, 2011^25^ | Bladder cancer | general/cases | CS/CC | highest vs. lowest | 4 | 2,341/203,918 | RR | 0.76 (0.47, 1.21) | NA | 0.73 | NA |
| Acham, 2020^26^ | Bladder cancer | general | CS | highest vs. lowest | 4 | 3,253/597,227 | HR | 0.97 (0.79, 1.17) | NA | NA | NA |
| Aune, 2012^27^ | Colorectal cancer | general | CS | highest vs. lowest | 7 | 1,635/177,551 | RR | 0.94 (0.75, 1.18) | NA | NA | 39 |
|  |  | general | CS | per 50g/d | 7 | 1,635/177,551 | RR | 0.96 (0.83, 1.12) | NA | NA | 28 |
| Barrubés, 2019^28^ | Colorectal cancer | general | CS | highest vs. lowest | 4 | 5857/590,352 | RR | 0.85 (0.76, 0.96) | <0.01 | NA | 27 |
|  |  | general/cases | CC | highest vs. lowest | 5 | 2,221/5,439 | OR | 0.95 (0.79, 1.14) | 0.56 | NA | 0 |
|  | Colon cancer | general | CS | highest vs. lowest | 4 | >3,687/>583,136 | RR | 0.88 (0.77, 1.01) | 0.06 | NA | 44 |
|  |  | general/cases | CC | highest vs. lowest | 3 | 2,225/4,894 | OR | 0.87 (0.74, 1.02) | 0.09 | NA | 0 |
|  | Rectal cancer | general | CS | highest vs. lowest | 3 | 2,067/583,136 | RR | 0.93 (0.76, 1.13) | 0.44 | NA | 0 |
|  |  | general/cases | CC | highest vs. lowest | 2 | 945/1,904 | OR | 0.77 (0.55, 1.09) | 0.14 | NA | 0 |
| Vieira, 2017^29^ | Colorectal cancer | general | CS | per 50g/d | 7 | 6,462/NA | RR | 0.94 (0.87, 1.02) | NA | 0.72 | 9.5 |
|  | Colon cancer | general | CS | per 50g/d | 6 | 3,958/NA | RR | 0.91 (0.80, 1.03) | NA | NA | 18.5 |
|  | Rectal cancer | general | CS | per 50g/d | 4 | 2,101/NA | RR | 0.95 (0.90, 1.00) | NA | NA | 0 |
| Ralston, 2014^30^ | Colorectal cancer | general | CS | highest vs. lowest  (Solid cheese) | 7 | 1,347/283,225 | RR | 1.11 (0.90, 1.36) | 0.34 | NA | 16 |
| Cho, 2004^31^ | Colorectal cancer | general | CS | highest vs. lowest | 10 | 7,157/534,536 | RR | 1.10 (0.98, 1.24) | NA | NA | NA |
|  | Colon cancer | general | CS | highest vs. lowest | 10 | 2,912/534,536 | RR | 1.14 (0.95, 1.36) | NA | NA | NA |
|  | Rectal cancer | general | CS | highest vs. lowest | 10 | 1,208/534,536 | RR | 1.08 (0.86, 1.36) | NA | NA | NA |
| Jin, 2020^6^ | Colorectal cancer | general | CS | highest vs. lowest | 11 | 8,690  /1,025,510 | RR | 0.95 (0.83, 1.08) | NA | >0.1 | 23.1 |
|  |  | general | CS | per 50g/d | 8 | 7,854  /867,812 | RR | 0.93 (0.89, 0.97) | NA | >0.1 | 0 |
|  | Colon cancer | general | CS | highest vs. lowest | 7 | 4,323/750,793 | RR | 0.95 (0.76, 1.20) | NA | >0.1 | 48.3 |
|  | Rectal cancer | general | CS | highest vs. lowest | 5 | 2,212/670,807 | RR | 0.93 (0.77, 1.12) | NA | >0.1 | 0 |
| Liang, 2022^32^ | Colorectal cancer | general/cases | CC | highest vs. lowest | 7 | 6,544/14,666 | OR | 0.89 (0.82, 0.97) | <0.05 | NA | 0 |
|  | Colon cancer | general/cases | CC | highest vs. lowest | 4 | 2,944/4,373 | OR | 0.89 (0.79, 1.00) | <0.05 | NA | 0 |
|  | Rectal cancer | general/cases | CC | highest vs. lowest | 3 | 1,669/2,412 | OR | 0.86 (0.74, 1.00) | <0.05 | NA | 0 |
|  | Colorectal cancer | general | CS | highest vs. lowest | 5 | 1,704/193,216 | RR | 0.89 (0.73, 1.08) | >0.05 | NA | 37 |
|  | Colon cancer | general | CS | highest vs. lowest | 5 | 1,052/186,000 | RR | 0.88 (0.68, 1.13) | >0.05 | NA | 41.1 |
|  | Rectal cancer | general | CS | highest vs. lowest | 2 | 422/106,014 | RR | 0.84 (0.54, 1.29) | >0.05 | NA | 0 |
| Li, 2018^33^ | Endometrial cancer | general/cases | CC/CS | highest vs. lowest | 5 | 3,943/509,785 | OR | 0.89 (0.76, 1.05) | NA | NA | 39 |
|  |  | general/cases | CC | highest vs. lowest | 3 | 2,834/506,970 | OR | 0.81 (0.53, 1.24) | NA | NA | 59.5 |
|  |  | general | CS | highest vs. lowest | 2 | 1109/2,815 | OR | 0.91 (0.77, 1.07) | NA | NA | 37.4 |
| Yang, 2017^34^ | Hepatocellular carcinoma | general/cases | CC/CS | highest vs. lowest | 3 | 421/477,938 | RR | 1.45 (1.02, 2.07) | NA | NA | 0 |
| Liao, 2020^35^ | Ovarian cancer | general/cases | CC/CS | highest vs. lowest | 12 | 7,080/143,628 | RR | 1.00 (0.85, 1.17) | 0.993 | 0.266 | 60.1 |
|  |  | general/cases | CC/CS | per 100g/d | 9 | NA/1,807,220 | RR | 0.87 (0.76, 0.98) | 0.025 | NA | NA |
| Khodavandi, 2021^36^ | Ovarian cancer | general | CS | highest vs. lowest  (Total cheese) | 8 | 2,627/913,008 | RR | 1.11 (0.97, 1.28) | 0.132 | NA | 0 |
|  |  | general | CS | highest vs. lowest  (Hard cheese) | 3 | 2,734/713,801 | RR | 0.88 (0.56, 1.39) | 0.58 | NA | 75.7 |
|  |  | general | CS | highest vs. lowest  (Cottage cheese) | 3 | 2,579/665,468 | RR | 0.90 (0.69, 1.18) | 0.464 | NA | 0 |
| Genkinger, 2006^37^ | Ovarian cancer | general | CS | highest vs. lowest  (Hard cheese) | 12 | 2,027/ 553,217 | RR | 1.30 (0.96, 1.78) | NA | NA | NA |
|  |  | general | CS | highest vs. lowest  (Cottage cheese) | 12 | 1,464/ 553,217 | RR | 0.88 (0.63, 1.23) | NA | NA | NA |
| Aune, 2015^38^ | Prostate cancer | general | CS | highest vs. lowest | 11 | 22,950/887,759 | RR | 1.07 (1.01, 1.13) | NA | 0.57 | 0 |
|  |  | general | CS | per 50g/d | 11 | 22,950/887,759 | RR | 1.10 (1.03, 1.18) | NA | NA | 0 |
| Zhao, 2022^39^ | Prostate cancer | general | CS | highest vs. lowest | 15 | 53,518/1,328,775 | RR | 1.03 (0.99, 1.08) | NA | NA | 0 |
|  |  | general | CS | per 40g/d | 15 | 53,518/1,328,775 | RR | 1.01 (1.00, 1.03) | NA | NA | NA |
| Arafa, 2021^40^ | Pancreatic cancer | general | CS | highest vs. lowest | 2 | 2,371/911,982 | HR | 1.16 (0.87, 1.55) | NA | NA | 0 |
| Wang, 2016^41^ | Non-Hodgkin lymphoma | general/cases | CC/CS | highest vs. lowest | 10 | 5,519/423,441 | RR | 1.14 (0.96, 1.34) | NA | 0.278 | 58.2 |
|  |  | general/cases | CC | highest vs. lowest | 9 | 4,252/13,030 | RR | 1.14 (0.94, 1.38) | NA | NA | 61.9 |
|  |  | general | CS | highest vs. lowest | 1 | 1,267/410,411 | RR | 1.09 (0.85, 1.39) | NA | NA | NA |
| Guo, 2021^42^ | Conventional and serrated Precursors of colorectal cancer | general/cases | CC/CS | highest vs. lowest | 5 | 1,973/23,453 | RR | 0.96 (0.93, 0.99) | 0.017 | NA | 0 |
|  |  | general | CS | highest vs. lowest | 2 | 1,197/22,548 | RR | 0.99 (0.81, 1.22) | 0.94 | NA | 26.6 |
|  |  | general/cases | CC | highest vs. lowest | 3 | 776/905 | RR | 0.96 (0.93, 0.99) | 0.016 | NA | 0 |

**Abbreviation：** CS=cohort study; CC= case-control study; RR= relative risk; OR= odds ratio; HR= hazard ratio; NA= not available.

# Supplementary Table 6. Description of meta-analyses on cheese consumption and metabolic disease risk included in the umbrella review.

| **Author, year** | **Outcome** | **Population** | **Study design** | **Comparison/Intake** | **No of studies** | **Cases/Total** | **Metric** | **Effect size**  **(95% CI)** | ***P* value** | | **Egger’s *P*** | ***I*^2^**  **(%)** |
| --- | --- | --- | --- | --- | --- | --- | --- | --- | --- | --- | --- | --- |
| Aune, 2013^43^ | type 2 diabetes mellitus | general | CS | per 50g/d | 8 | 17,620/242,960 | RR | 0.92 (0.86, 0.99) | NA | 0.74 | | 0 |
|  |  | general | CS | highest vs. lowest | 8 | 17,620/242,960 | RR | 0.91 (0.84, 0.98) | NA | 0.74 | | 0 |
|  |  | general | CS | highest vs. lowest  (Cottage cheese) | 2 | 2,846/78,437 | RR | 0.91 (0.79, 1.04) | NA | NA | | 0 |
| Fan, 2019^44^ | type 2 diabetes mellitus | general | CS | highest vs. lowest | 15 | 9,907/201,451 | RR | 0.94 (0.89, 1.00) | NA | 0.656 | | 1.9 |
|  |  | general | CS | per 30g/d | 11 | 9,484/190,637 | RR | 0.97 (0.93, 1.02) | NA | NA | | 13.9 |
| Gao, 2013^45^ | type 2 diabetes mellitus | general | CS | highest vs. lowest | 7 | 14,810/178,429 | RR | 0.82 (0.77, 0.87) | NA | NA | | 0 |
|  |  | general | CS | per 30g/d | 7 | 14,810/178,429 | RR | 0.80 (0.69, 0.93) | NA | NA | | 59 |
| Gijsbers, 2016^46^ | type 2 diabetes mellitus | general | CS | per 10g/d | 13 | 32,936/369,697 | RR | 1.00 (0.99, 1.02) | 0.65 | 0.88 | | 61.7 |
| Khoramdad, 2017^47^ | type 2 diabetes mellitus | general | CS | highest vs. lowest | 5 | 2,683/76,510 | RR^*^ | 0.92 (0.82, 1.04) | NA | NA | | 17.3 |
|  |  | general | CS | highest vs. lowest | 5 | 14,908/234,913 | RR^**^ | 1.04 (0.93, 1.16) | NA | NA | | 36.2 |
| Jin, 2021^48^ | Metabolic syndrome | general | CS/ Cross-sectional study | highest vs. lowest | 8 | >2,145/129,822 | RR | 0.98 (0.86, 1.11) | NA | >0.05 | | 85.6 |
|  |  | general | CS | highest vs. lowest | 4 | 2,145/10,290 | RR | 1.03 (0.87, 1.22) | NA | NA | | 82.1 |
|  |  | general/cases | Cross-sectional study | highest vs. lowest | 4 | 119,532 | RR | 0.91 (0.74, 1.14) | NA | NA | | 89.7 |
|  |  | general | CS | per 50g/d | 3 | 1,555/6,674 | RR | 0.99 (0.73, 1.35) | NA | NA | | 86.2 |
| Babio, 2022^49^ | Overweight/Obesity | general/cases | Cross-sectional study | highest vs. lowest | 3 | 1,646/8,565 | RR | 0.86 (0.63, 1.16) | NA | NA | | 14.4 |
|  |  | general | CS | highest vs. lowest | 1 | NA/8,763 | RR | 0.91 (0.73, 1.13) | NA | NA | | NA |

*relative risk; **rate ratio

**Abbreviation：**CS=cohort study; CC= case-control study; RR= relative risk; OR= odds ratio; HR= hazard ratio; NA= not available.

# Supplementary Table 7. Description of meta-analyses on cheese consumption and aging-related disease risk included in the umbrella review.

| **Author, year** | **Outcome** | **Population** | **Study design** | **Comparison/Intake** | **No of studies** | **Cases/Total** | **Metric** | **Effect size**  **(95% CI)** | ***P* value** | **Egger’s *P*** | ***I^2^***  **(%)** |
| --- | --- | --- | --- | --- | --- | --- | --- | --- | --- | --- | --- |
| Ong, 2020^50^ | Hip fracture | general | CS | highest vs. lowest | 2 | 2,214/81,069 | RR | 0.89 (0.73, 1.10) | NA | NA | 0 |
| Matía-Martín, 2019^51^ | Hip fracture | general | CS | highest vs. lowest | 6 | 8,411/236,136 | HR | 0.80 (0.62, 1.03) | NA | NA | 86.5 |
|  |  | general | CS | per 1 increment | 5 | 8,354/231,442 | HR | 0.96 (0.88, 1.04) | NA | NA | 90.8 |
|  | Fracture at any site | general | CS | highest vs. lowest | 3 | 22,944/109,134 | HR | 0.89 (0.81, 0.98) | NA | NA | 59 |
| Bian, 2018^52^ | Hip fracture | general | CS | highest vs. lowest | 4 | 5,579/109,018 | RR | 0.68 (0.61, 0.77) | NA | NA | 19.5 |
|  |  | general/cases | CC | highest vs. lowest | 3 | 1,071/3,022 | OR | 0.77 (0.53, 1.11) | NA | NA | 27.6 |
| Hidayat, 2020^53^ | Hip fracture | general | CS | highest vs. lowest | 8 | 8,860/305,157 | RR | 0.85 (0.66, 1.08) | NA | >0.45 | 76.9 |
| Jiang, 2014^54^ | Parkinson’s disease | general | CS | highest vs. lowest | 5 | 955/296,689 | RR | 1.26 (0.99, 1.60) | NA | NA | 29.2 |
|  |  | general | CS | per 10g/d | 3 | 659/ 271,282 | RR | 1.13 (0.91, 1.40) | NA | NA | NA |

**Abbreviation：** CS=cohort study; CC= case-control study; RR= relative risk; OR= odds ratio; HR= hazard ratio; NA= not available.

# Supplementary Table 8. List of studies excluded from updated meta-analysis and reasons for exclusion.

| **Outcome** | **Author, year** | **Reason(s) for exclusion** |
| --- | --- | --- |
| **Metabolism related health outcomes** | | |
| Type 2 diabetes mellitus | Drouin-Chartier, 2019^55^ | Use the same cohort (NHS、NHS II、HPFS) as study by Chen, 2014 [^56^] with greater population size and the identical health outcome |
| Type 2 diabetes mellitus | Yuzbashian, 2021^57^ | Not applicable for data summary and analysis due to the different units (reported effect sizes for change in cheese consumption). |
| Metabolic syndrome | Cheraghi, 2018^58^ | Use the same cohort (TLGS) as study by Yuzbashian, 2021 [^59^] with the identical health outcome |
| Metabolic syndrome | Fumeron, 2011^60^ | Use the same cohort (DESIR) as study by Fumeron, 2011 [^61^] with the identical health outcome |
| **Cancer incidence** | | |
| Ovarian cancer | Fairfield, 2004^62^ | Use cohort (NHS) included in the study by Genkinger, 2006^37^ with the identical health outcome. |
| Ovarian cancer | Kushi, 1999^63^ | Use cohort (IWHS) included in the study by Genkinger, 2006^37^ with the identical health outcome. |
| Ovarian cancer | Larsson, 2004^64^ | Use cohort (SMC) included in the study by Genkinger, 2006^37^ with the identical health outcome. |
| Ovarian cancer | Bertone, 2002^65^ | Use cohort (NHS) included in the studies by Fairfield, 2004^62^ and Genkinger, 2006^37^ with the identical health outcome. |
| Breast cancer | Genkinger, 2013^66^ | Use cohort (BWHS) included in the study by Wu, 2021^24^ with the identical health outcome. |
| Colorectal cancer | Kampman, 1994^67^ | Use cohort (NLCS) included in the study by Cho, 2004^31^ with the identical health outcome. |
| Colon cancer | Kearney, 1996^68^ | Use cohort (HPFS) included in the study by Cho, 2004^31^ with the identical health outcome. |
| Colorectal cancer | Kesse, 2005^69^ | Use sub-cohort (E3N-EPIC) of that (EPIC) in the study by Murphy, 2013^70^ with the identical health outcome. |
| Bladder cancer | Keszei, 2010^71^ | Use cohort (NLCS) included in the study by Acham, 2020^26^ with the identical health outcome. |
| Ovarian cancer | Kiani, 2006^72^ | Use cohort (AHS) included in the study by Genkinger, 2006^37^ with the identical health outcome. |
| Ovarian cancer | Koralek, 2006^73^ | Use cohort (BCDDP) included in the study by Genkinger, 2006^37^ with the identical health outcome. |
| Colorectal cancer | Larsson, 2005^74^ | Use cohort (SMC) included in the study by Cho, 2004^31^ with the identical health outcome. |
| Pancreatic cancer | Michaud, 2003^75^ | Use cohort (NHS) included in the study by Genkinger, 2014^76^ with the identical health outcome. |
| Ovarian cancer | Mommers, 2006^77^ | Use cohort (NLCS) included in the study by Genkinger, 2006^37^ with the identical health outcome. |
| Breast cancer | Shin, 2002^78^ | Use cohort (NHS) included in the study by Wu, 2021^24^ with the identical health outcome. |
| Colorectal cancer | Singh, 1998^79^ | Use cohort (AHS) included in the study by Cho, 2004^31^ with the identical health outcome. |
| Breast cancer | Voorrips, 2002^80^ | Use cohort (NLCS) included in the study by Wu, 2021^24^ with the identical health outcome. |
| Colorectal cancer | Papadimitriou, 2022^81^ | The results of EPIC cohort are covered by another study (Murphy, 2013^70^) with greater population size and the identical health outcome, and the rest of the results from NLCS cohort are not applicable for data summary and analysis due to the lack of units. |
| **All-cause mortality** | | |
| All-cause mortality | Bongard, 2016^82^ | Use cohort (the MONICA Project) included in the study by Tognon, 2017^83^ with the identical health outcome. |
| All-cause mortality | Fraser, 1997^84^ | Use the same cohort (AHS) as study by Fraser, 1997^85^ with the identical health outcome. |
|  | Zupo, 2020^86^ | Not applicable for unit transformation and data summary due to the different unit (per times/d) |

**Abbreviation：**NHS= Nurses’ Health Study; NHS II= Nurses’ Health Study II; HPFS=Health Professional Follow-up Study; TLGS= Tehran Lipid and Glucose Study; DESIR= Data from an Epidemiological Study on the Insulin Resistance Syndrome; IWHS= Iowa Women’s Health Study; SMC= Swedish Mammography Cohort; BWHS= Black Women's Health Study; NLCS= Netherlands Cohort Study/ Netherlands Cohort Study on Diet and Cancer; E3N-EPIC= Etude Epidémiologique de femmes de la Mutuelle Générale de l'Education Nationale cohort study; EPIC= European Prospective Investigation into Cancer and Nutrition; NLCS= Netherlands Cohort Study/ Netherlands Cohort Study on Diet and Cancer; AHS= Adventist Health Study; the MONICA Project= Northern Sweden Multinational Monitoring of Trends and Determinants in Cardiovascular Disease.

# Supplementary Table 9. Description of original studies on the association between cheese consumption and other health outcomes.

| **Author, year** | **Study design** | **Cohort** | **Population** | **Country** | **Comparison** | **Outcome** | **Cases/Total** | **Dietary assessment** | **Metric** | **Effect size (95%CI)** | ***P* value** |
| --- | --- | --- | --- | --- | --- | --- | --- | --- | --- | --- | --- |
| Downer, 2017^87^ | CS | NA | prostate cancer | Sweden | ≥2servings/d vs. <1servings/d | Prostate cancer mortality | 222/525 | 68-item FFQ | HR | 0.94 (0.65, 1.35) | 0.68 |
| Downer, 2017^87^ | CS | NA | prostate cancer | Sweden | ≥2servings/d vs. <1servings/d | Overall mortality | 490/525 | 68-item FFQ | HR | 0.96 (0.76, 1.21) | 0.98 |
| Dik, 2014^88^ | CS | EPIC | colorectal cancer | Europe | >49g/d vs. <15g/d | Colorectal cancer-specific death | 1,028/3,859 | FFQ | HR | 0.93(0.76, 1.14) | 0.48 |
| Dik, 2014^88^ | CS | EPIC | colorectal cancer | Europe | per 25g/d increase | Colorectal cancer-specific death | 1,028/3,859 | FFQ | HR | 0.98 (0.92, 1.05) | NA |
| Dik, 2014^88^ | CS | EPIC | colorectal cancer | Europe | >49g/d vs. <15g/d | All-cause death | 1,525/3,859 | FFQ | HR | 0.87(0.74, 1.04) | 0.19 |
| Dik, 2014^88^ | CS | EPIC | colorectal cancer | Europe | per 25g/d increase | All-cause death | 1,525/3,859 | FFQ | HR | 0.98 (0.93, 1.03) | NA |
| Andersen, 2020^89^ | CS | DCH | breast cancer | Denmark | per 50g/d increment (pre-diagnosis) | Breast cancer recurrence | 309/1,965 | FFQ | HR | 1.17 (0.94, 1.45) | NA |
| Andersen, 2020^89^ | CS | DCH | breast cancer | Denmark | per 50g/d increment (pre-diagnosis) | Breast cancer mortality | 301/1,965 | FFQ | HR | 1.18 (0.94, 1.47) | NA |
| Andersen, 2020^89^ | CS | DCH | breast cancer | Denmark | per 50g/d increment (pre-diagnosis) | All-cause mortality | 460/1,965 | FFQ | HR | 1.16 (0.97, 1.39) | NA |
| Andersen, 2020^89^ | CS | DCH | breast cancer | Denmark | per 50g/d increment (post-diagnosis) | Breast cancer recurrence | 152/977 | FFQ | HR | 1.23 (0.85, 1.78) | NA |
| Andersen, 2020^89^ | CS | DCH | breast cancer | Denmark | per 50g/d increment (post-diagnosis) | Breast cancer mortality | 121/977 | FFQ | HR | 0.95 (0.66, 1.37) | NA |
| Andersen, 2020^89^ | CS | DCH | breast cancer | Denmark | per 50g/d increment (post-diagnosis) | All-cause mortality | 175/977 | FFQ | HR | 1.09 (0.70, 1.47) | NA |
| Miyake, 2010^90^ | CS | OMCHS | general | Japan | 12.5g/d vs. 0g/d | Wheeze in infants | 169/763 | SAQ | OR | 0.51 (0.31, 0.85) | 0.02 |
| Miyake, 2010^90^ | CS | OMCHS | general | Japan | 12.5g/d vs. 0g/d | Eczema in infants | 142/763 | SAQ | OR | 0.67 (0.38, 1.16) | 0.16 |
| Yalçin, 2010^91^ | NCC | NA | general | Turkey | non-colic group: 223g/w vs. colic cases: 184g/w | Infant colic | 47/189 | FFQ | OR | 0.89 (0.79, 0.99) | 0.03 |
| Ito, 2019^92^ | CS | JECS | general | Japan | ≥3 times/w vs. <1 time/w | Preterm birth | 2,343/77,667 | FFQ | OR | 0.98 (0.87, 1.10) | 0.691 |
| Nicklaus, 2018^93^ | CS | PASTURE | general | Europe | yes vs. no | Atopic dermatitis | 211/722 | Questionnaire | OR | 0.71 (0.35, 1.44) | NA |
| Nicklaus, 2018^93^ | CS | PASTURE | general | Europe | yes vs. no | Food allergy | 48/724 | Questionnaire | OR | 1.13 (0.25, 5.05) | NA |
| Nicklaus, 2018^93^ | CS | PASTURE | general | Europe | yes vs. no | Allergic rhinitis | 52/751 | Questionnaire | OR | 1.00 (0.29, 3.54) | NA |
| Nicklaus, 2018^93^ | CS | PASTURE | general | Europe | yes vs. no | Asthma | 54/722 | Questionnaire | OR | 0.78 (0.26, 2.36) | NA |
| Opstelten, 2016^94^ | NCC | EPIC | general | Europe | 57.6-248.2g/d vs. 0.0-16.5g/d | Crohn’s disease | 110/548 | 200-item FFQ | OR | 0.85 (0.42, 1.72) | 0.64 |
| Opstelten, 2016^94^ | NCC | EPIC | general | Europe | 52.5-678.2g/d vs. 0.0-15.8g/d | Ulcerative colitis | 239/1,210 | 200-item FFQ | OR | 0.83 (0.52, 1.32) | 0.6 |
| Miyake, 2016^95^ | CS | KOMCHS | general | Japan | 10.7g/d vs. 0.0g/d | Postpartum depressive symptoms | 108/1,319 | DHQ | OR | 0.74 (0.38, 1.46) | 0.18 |
| Berkey, 2013^96^ | CS | GUTS | general | USA | no-BBD:0.46 slices/d vs.  BBD:0.45 slices/d | Benign breast disease | 105/6,860 | FFQ | OR | 0.99 (0.71, 1.40) | NA |
| Orta, 2020^97^ | CS | NHS II | general | USA | >2servings/d vs. ≤4servings/w | Uterine leiomyoma | 8,142/81,590 | over 130-item FFQ | HR | 0.97 (0.79, 1.19) | 0.51 |
| Yuan, 2021^98^ | CS | SMC; COSM | general | Sweden | per 1-serving increase | Venous thromboembolism | 5,241/81,507 | 96-item FFQ | HR | 0.98 (0.95, 1.01) | 0.11 |
| Yuan, 2021^98^ | CS | SMC; COSM | general | Sweden | per 1-serving increase | Deep vein thrombosis | 2,722/81,507 | 96-item FFQ | HR | 0.96 (0.92, 1.00) | 0.06 |
| Niinistö, 2014^99^ | CS | DIPP | general | Finland | >86g/d vs. 58.5g/d | Preclinical type 1 diabetes | 240/4,887 | FFQ | HR | 0.74 (0.52, 1.04) | 0.127 |
| Niinistö, 2014^99^ | CS | DIPP | general | Finland | >86g/d vs. 58.5g/d | Type 1 diabetes | 112/4,887 | FFQ | HR | 0.55 (0.33, 0.94) | 0.023 |
| Tanaka, 2012^100^ | CS | OMCHS | general | Japan | 10.1g/d vs. 0.5g/d | Dental caries | 74/105 | DHQ | OR | 0.37 (0.17, 0.76) | 0.01 |
| Matthews, 2011^101^ | CS | AHS-2 | general | USA | ≥1/w vs. 1-3/m | Osteoporosis | 100/337 | FFQ | OR | 0.28 (0.12, 0.66) | 0.004 |
| Camacho‑Barcia, 2019^102^ | CS | PREDIMED | general | Spain | 41.6g/d vs. 11.6g/d | Cataract | 768/5,860 | 137-item FFQ | HR | 1.04 (0.87, 1.25) | 0.741 |
| Littlejohns, 2020^103^ | CS | UKB | general | UK | 7+ servings/w vs. never | Kidney stones | 2,012/439,072 | FFQ | HR | 0.98 (0.79, 1.23) | 0.5 |
| Littlejohns, 2020^103^ | CS | UKB | general | UK | per 25g/w increase | Kidney stones | 2,012/439,072 | FFQ | HR | 0.95 (0.82, 1.10) | 0.5 |

**Abbreviation：**CS=cohort study; NCC= nested case-control study; RR= relative risk; OR= odds ratio; HR= hazard ratio; NA= not available; FFQ= food frequency questionnaire; DHQ= diet history questionnaire; 24-hDR = 24-h dietary recalls; SAQ= self-administered questionnaire; EPIC= European Prospective Investigation into Cancer and Nutrition; DCH= the Danish Diet, Cancer and Health (DCH) cohort; OMCHS= Osaka Maternal and Child Health Study; JECS= the Japan Environment and Children’s Study; PASTURE= Protection against Allergy—STUdy in Rural Environment; KOMCHS=the Kyushu Okinawa Maternal and Child Health Study; GUTS= Growing Up Today Study; NHSII= Nurses’ Health Study II; SMC= Swedish Mammography Cohort; COSM= Cohort of Swedish Men; DIPP= the Finnish Type 1Diabetes Prediction and Prevention (DIPP) Study; AHS-2=Adventist Health Study-2; PREDIMED=Prevencióncon Dieta Mediterránea; UKB=UK Biobank.

# Supplementary Table 10. Association between cheese consumption (highest vs. lowest intake level) and all-cause and cause-specific mortality.

| **Outcome** | **Population** | **Study design** | **Studies**  **(n)** | **Cases/Total** | **RR (95% CI)** | ***P* value** | **95% prediction interval** | **Egger’s *P*** | ***I*^2^**  **(%)** | ***P* value for excess significance test** | **Type** | **Evidence class** |
| --- | --- | --- | --- | --- | --- | --- | --- | --- | --- | --- | --- | --- |
| **All-cause mortality** | | | | | | | | | | | | |
| All-cause | general | CS | 21 | 136,298/>1,030,243 | 0.95 (0.92, 0.99) | 0.0052 | 0.87, 1.05 | 0.65 | 34 | 0.29 | updated | Moderate |
| **Cancer mortality** | | | | | | | | | | | | |
| Overall cancer | general | CS/NCC | 25 | 30,818/1,378,932 | 1.00 (0.97, 1.03) | 0.91 | 0.96, 1.04 | 0.65 | 1 | 0.31 | updated | Moderate |
| Colorectal cancer | general | CS | 4 | 751/161,067 | 1.01 (0.75, 1.36) | 0.95 | 0.32, 3.22 | 0.16 | 53 | 0.55 | updated | Low |
| Colon cancer | general | CS | 3 | 236/133,317 | 1.18 (0.73, 1.89) | 0.51 | 0.01, 170.04 | 0.17 | 53 | 0.51 | updated | Low |
| Rectal cancer | general | CS | 2 | 114/133,317 | 1.12 (0.73, 1.73) | 0.60 | NA | NA | 0 | 1.00 | updated | Low |
| Prostate cancer | general | CS | 2 | 275/300,651 | 1.41 (0.91, 2.20) | 0.12 | NA | NA | 0 | 1.00 | updated | Very low |
| Lung cancer | general | CS | 6 | >781/339,356 | 0.85 (0.63, 1.15) | 0.32 | 0.37, 1.95 | 0.77 | 41.5 | 0.73 | previous | Low |
| Pancreatic cancer | general | CS | 4 | >25/220,913 | 1.25 (0.89, 1.76) | 0.21 | 0.13, 11.66 | 0.78 | 0 | 1.00 | previous | Very low |
| Breast cancer | general | CS/NCC | 2 | >142/169,147 | 1.16 (0.88, 1.53) | 0.28 | NA | NA | 0 | 1.00 | previous | Very low |
| Ovarian cancer | general | CS | 2 | >77/232,480 | 1.24 (0.81, 1.90) | 0.32 | NA | NA | 0 | 1.00 | previous | Very low |
| Gastric cancer | general | CS | 2 | 910/113,950 | 1.02 (0.66, 1.58) | 0.93 | NA | NA | 0 | 1.00 | previous | Low |
| Urothelial cancer | general | CS | 1 | 46/114,517 | 0.91 (0.46, 1.82) | NA | NA | NA | NA | NA | previous | NA |
| **Cardiovascular mortality** | | | | | | | | | | | | |
| CVD | general | CS | 16 | 36,965/742,571 | 0.93 (0.88, 0.99) | 0.015 | 0.81, 1.07 | 0.78 | 34 | 0.30 | updated | Moderate |
| CHD | general | CS | 7 | 4,843/233,926 | 0.98 (0.86, 1.12) | 0.78 | 0.69, 1.39 | 0.9996 | 53 | 0.77 | updated | Low |
| Stroke | general | CS | 4 | 1,508/197,664 | 0.76 (0.58, 1.01) | 0.054 | 0.28, 2.07 | 0.64 | 42 | 0.78 | updated | Very low |

**Abbreviation:** CS=cohort study; NCC=nested case-control study; NA= not available; NS= not significant; CVD=cardiovascular disease; CHD=coronary heart disease; RR= relative risk.

# Supplementary Table 11. Association between cheese consumption (highest vs. lowest intake level) and disease risk.

| **Outcome** | **Population** | **Study design** | **Studies**  **(n)** | **Cases/Total** | **RR (95% CI)** | ***P* value** | **95% prediction interval** | **Egger’s *P*** | ***I*^2^**  **(%)** | ***P* value for excess significance test** | **Type** | **Evidence class** |
| --- | --- | --- | --- | --- | --- | --- | --- | --- | --- | --- | --- | --- |
| **Cardiovascular disease** | | | | | | | | | | | | |
| Overall CVD | general | CS | 18 | 86,796/1,833,112 | 0.92 (0.89, 0.96) | 0.0001 | 0.83, 1.02 | 0.71 | 38 | 0.49 | updated | Moderate |
| Coronary heart disease | general | CS | 12 | 17,568/686,573 | 0.92 (0.86, 0.98) | 0.0108 | 0.79, 1.07 | 0.50 | 27 | 0.72 | updated | Moderate |
| Stroke | general | CS | 9 | 21,138/813,636 | 0.93 (0.89, 0.98) | 0.003 | 0.88, 0.99 | 0.92 | 0 | 0.46 | updated | Moderate |
| Hypertension | general | CS | 11 | >103,698/756,385 | 0.96 (0.91, 1.02) | 0.20 | 0.85, 1.09 | 0.20 | 48 | 0.27 | updated | Moderate |
| **Cancer** | | | | | | | | | | | | |
| Overall cancer | general | CS/CC | 47 | 158,743/9,540,697 | 0.99 (0.97, 1.01) | 0.30 | 0.97, 1.01 | 0.04 | 19 | 0.43 | updated | Low |
| Prostate cancer | general | CS/CC | 12 | 40,846/1,215,512 | 1.03 (0.98, 1.08) | 0.21 | 0.95, 1.12 | 0.02 | 0 | 0.52 | updated | Moderate |
| Advanced prostate cancer | general | CS | 2 | 3,774/456,704 | 0.87 (0.67, 1.13) | 0.30 | NA | NA | 54 | 0.56 | updated | Very low |
| Colorectal cancer | general | CS | 10 | 16,986/1,744,478 | 1.02 (0.92, 1.13) | 0.76 | 0.79, 1.30 | 0.61 | 41 | 0.78 | updated | Low |
| Colon cancer | general | CS | 6 | 7,381/779,173 | 0.95 (0.80, 1.12) | 0.51 | 0.60, 1.49 | 0.53 | 59 | 0.80 | updated | Low |
| Proximal colon cancer | general | CS | 3 | 2,927/1,056,964 | 0.91 (0.63, 1.31) | 0.61 | 0.01, 63.96 | 0.69 | 83 | 0.38 | updated | Very low |
| Distal colon cancer | general | CS | 3 | 2,635/1,056,964 | 0.97 (0.84, 1.14) | 0.74 | 0.36, 2.63 | 0.59 | 0 | 1.00 | updated | Low |
| Rectal cancer | general | CS | 6 | 3,624/779,173 | 1.02 (0.89, 1.17) | 0.77 | 0.84, 1.24 | 0.73 | 0 | 1.00 | updated | Low |
| Breast cancer | general | CS | 9 | 74,235/2,313,894 | 0.98 (0.96, 1.01) | 0.16 | 0.96, 1.01 | 0.68 | 46 | 0.84 | updated | Low |
| Estrogen-receptor positive breast cancer | general | CS | 2 | 31,952/1,833,420 | 0.99 (0.95, 1.02) | 0.41 | NA | NA | 0 | 1.00 | updated | Low |
| Estrogen-receptor-negative breast cancer | general | CS | 2 | 6,866/1,833,420 | 0.89 (0.82, 0.97) | 0.01 | NA | NA | 0 | 0.75 | updated | Low |
| Bladder cancer | general | CS | 3 | 4,514/780,950 | 0.94 (0.85, 1.04) | 0.24 | 0.48, 1.83 | 0.38 | 0 | 1.00 | updated | Low |
| Pancreatic cancer | general | CS | 3 | 2,464/960,598 | 1.05 (0.86, 1.30) | 0.61 | 0.27, 4.07 | 0.83 | 0 | 1.00 | updated | Low |
| Endometrial cancer | general | CS | 2 | 2,707/506,970 | 0.91 (0.77, 1.07) | 0.24 | NA | NA | 37 | 1.00 | updated | Very low |
| Hepatocellular carcinoma | general | CS | 2 | 355/622,051 | 1.17 (0.67, 2.04) | 0.59 | NA | NA | 73 | 0.57 | updated | Very low |
| Ovarian cancer | general | CS | 2 | 2,608/878,948 | 1.11 (0.91, 1.35) | 0.31 | NA | NA | 0 | 1.00 | updated | Very low |
| Lymphoma | general | CS | 1 | 1,334/410,411 | 1.10 (0.86, 1.39) | 0.45 | NA | NA | NA | NA | updated | NA |
| Precursors of colorectal cancer | general | CS | 2 | 1,197/23,745 | 0.99 (0.81, 1.22) | 0.94 | NA | NA | 27 | 1.00 | previous | Very low |
| **Metabolic disease** | | | | | | | | | | | | |
| Type 2 diabetes | general | CS/NCC | 25 | 44,584/674,107 | 0.93 (0.88, 0.98) | 0.009 | 0.78, 1.10 | 0.07 | 45 | 0.92 | updated | Low |
| Prediabetes | general | CS | 3 | 2,851/10,899 | 0.93 (0.78, 1.12) | 0.45 | 0.12, 6.97 | 0.79 | 66 | 0.65 | de novo | Very low |
| Metabolic syndrome | general | CS | 4 | >1630/6,942 | 1.04 (0.79, 1.36) | 0.80 | 0.36, 3.02 | 0.63 | 69 | 0.75 | updated | Very low |
| Overweight/obesity | general | CS | 2 | > 8,238/25,651 | 0.99 (0.88, 1.11) | 0.86 | NA | NA | 0 | 1.00 | updated | Low |
| **Aging-related disease** | | | | | | | | | | | | |
| Total fracture | general | CS | 7 | 26,401/306,639 | 0.90 (0.86, 0.95) | <0.0001 | 0.85, 0.96 | 0.22 | 0 | 0.67 | updated | Low |
| Hip fracture | general | CS | 7 | 9,097/306,639 | 0.86 (0.72, 1.04) | 0.11 | 0.49, 1.51 | 0.06 | 71 | 0.78 | updated | Low |
| **Other health outcomes** | | | | | | | | | | | | |
| Fall | general | CS | 2 | 1,002/11,908 | 0.96 (0.82, 1.12) | 0.59 | NA | NA | 0 | 1.00 | de novo | Low |
| Frailty | general | CS | 2 | 284/2,694 | 1.07 (0.71, 1.59) | 0.76 | NA | NA | 0 | 1.00 | de novo | Very low |
| Dementia | general | CS | 2 | 4,619/252,008 | 0.81 (0.66, 0.99) | 0.04 | NA | NA | 0 | 0.53 | de novo | Low |
| Parkinson’s disease | general | CS | 5 | 955/296,689 | 1.26 (0.99, 1.60) | 0.052 | 0.71, 2.26 | 0.43 | 29.2 | 0.74 | previous | Very low |

**Abbreviation:** CVD= cardiovascular disease; CHD= coronary heart disease; T2DM= type 2 diabetes; CS=cohort study; NCC=nested case-control study; CC=case-cohort study; NA= not available; NS= not significant; RR= relative risk.

# Supplementary Table 12. The detailed assessments of methodological quality of the included systematic reviews and meta-analyses.

| Study  (Supplementary reference) | #1.  PICO | *2.  Protocol | #3. Explained study design inclusion criteria | *4.  Adequacy of the literature search | #5.  Performed study selection in duplicate | #6.  Performed data extraction in duplicate | *7. Excluded studies | #8.  Included studies described in adequate detail | *9.  Risk of bias of included studies | #10. Reported sources of funding for included studies | *11.  Meta-analytical methods | #12. Assessed impact of risk of bias in each study | *13. Consideration of risk of bias when interpreting the results of the review | #14. Observed heterogeneity and impact explained | *15. Assessment of presence and likely impact of publication bias | #16. Reported own conflict of interests and funding | Quality class^1^ |
| --- | --- | --- | --- | --- | --- | --- | --- | --- | --- | --- | --- | --- | --- | --- | --- | --- | --- |
| O'Sullivan, 2013^1^ | yes | partial yes | yes | partial yes | yes | yes | partial yes | yes | partial yes | no | yes | yes | yes | yes | yes | yes | High |
| Guo, 2017^2^ | yes | partial yes | yes | partial yes | unknown | unknown | partial yes | yes | partial yes | yes | yes | yes | yes | yes | yes | yes | Moderate |
| Tong, 2017^3^ | yes | partial yes | yes | no | no | yes | partial yes | yes | partial yes | no | yes | yes | yes | yes | yes | yes | Low |
| Lu, 2016^4^ | yes | partial yes | yes | partial yes | unknown | yes | partial yes | yes | partial yes | no | yes | yes | yes | yes | yes | yes | Moderate |
| Jin, 2021^5^ | yes | partial yes | yes | partial yes | unknown | yes | partial yes | yes | partial yes | no | yes | yes | yes | yes | yes | yes | Moderate |
| Jin, 2020^6^ | yes | partial yes | yes | partial yes | unknown | yes | partial yes | yes | partial yes | no | yes | yes | yes | yes | yes | yes | Moderate |
| Sun, 2014^7^ | yes | partial yes | yes | partial yes | unknown | no | partial yes | yes | partial yes | no | yes | no | yes | yes | yes | no | Low |
| Yang, 2016^8^ | yes | partial yes | yes | partial yes | unknown | yes | partial yes | yes | partial yes | no | yes | yes | yes | yes | yes | yes | Moderate |
| Zhang, 2020^9^ | yes | partial yes | yes | partial yes | unknown | yes | partial yes | partial yes | no | no | yes | no | no | yes | yes | yes | Critically low |
| Alexander, 2016^10^ | yes | partial yes | yes | partial yes | yes | yes | partial yes | partial yes | no | no | yes | no | no | yes | yes | yes | Critically low |
| Chen, 2017^11^ | yes | partial yes | yes | partial yes | yes | yes | partial yes | yes | partial yes | no | yes | yes | no | yes | yes | yes | Low |
| Chen, 2021^12^ | yes | yes | yes | partial yes | yes | yes | yes | yes | partial yes | yes | yes | yes | yes | yes | yes | yes | High |
| Gholami, 2017^13^ | yes | partial yes | yes | partial yes | yes | yes | partial yes | partial yes | no | no | yes | no | yes | yes | yes | yes | Low |
| Jakobsen, 2021^14^ | yes | yes | yes | partial yes | yes | yes | yes | partial yes | partial yes | yes | yes | yes | yes | yes | yes | yes | High |
| Qin, 2015^15^ | yes | partial yes | yes | partial yes | yes | yes | partial yes | partial yes | partial yes | no | yes | no | yes | yes | yes | yes | Moderate |
| de Goede, 2016^16^ | yes | partial yes | yes | partial yes | no | no | partial yes | partial yes | partial yes | no | yes | yes | yes | yes | yes | yes | Low |
| Hu, 2014^17^ | yes | partial yes | yes | partial yes | yes | yes | partial yes | partial yes | partial yes | no | yes | yes | no | yes | yes | yes | Low |
| Heidari, 2021^18^ | yes | partial yes | yes | partial yes | yes | unknown | partial yes | yes | partial yes | yes | yes | yes | yes | yes | yes | yes | High |
| Soedamah-Muthu, 2012^19^ | yes | partial yes | yes | no | unknown | unknown | yes | partial yes | no | no | yes | no | no | yes | yes | yes | Critically low |
| Ralston, 2012^20^ | yes | partial yes | yes | partial yes | yes | yes | partial yes | yes | yes | no | yes | yes | yes | yes | no | yes | Low |
| Zhang, 2019^21^ | yes | partial yes | yes | partial yes | unknown | yes | partial yes | partial yes | no | no | yes | no | no | yes | yes | yes | Critically low |
| Boyd, 1993^22^ | yes | no | yes | no | unknown | unknown | yes | yes | partial yes | no | yes | yes | yes | yes | no | yes | Critically low |
| Kazemi, 2021^23^ | yes | yes | yes | partial yes | unknown | yes | partial yes | partial yes | partial yes | no | yes | yes | yes | yes | yes | yes | Moderate |
| Li, 2011^25^ | yes | partial yes | yes | partial yes | unknown | yes | partial yes | yes | no | no | yes | no | no | yes | yes | yes | Critically low |
| Aune, 2012^27^ | yes | partial yes | yes | no | unknown | yes | yes | yes | no | no | yes | no | no | yes | yes | yes | Critically low |
| Barrubés, 2019^28^ | yes | yes | yes | partial yes | yes | yes | partial yes | yes | partial yes | yes | yes | yes | yes | yes | no | yes | Low |
| Vieira, 2017^29^ | yes | partial yes | yes | partial yes | unknown | unknown | partial yes | no | no | no | yes | no | no | yes | yes | yes | Critically low |
| Ralston, 2014^30^ | yes | partial yes | yes | partial yes | unknown | yes | partial yes | yes | partial yes | no | yes | no | yes | yes | no | yes | Low |
| Liang, 2022^32^ | yes | partial yes | yes | partial yes | yes | yes | partial yes | yes | partial yes | yes | yes | yes | yes | yes | yes | yes | High |
| Li, 2018^33^ | yes | partial yes | yes | partial yes | unknown | yes | partial yes | yes | partial yes | no | yes | yes | no | yes | yes | yes | Low |
| Yang, 2017^34^ | yes | partial yes | yes | partial yes | unknown | yes | partial yes | yes | partial yes | no | yes | yes | yes | yes | yes | yes | Moderate |
| Liao, 2020^35^ | yes | partial yes | yes | partial yes | yes | unknown | partial yes | partial yes | partial yes | no | yes | yes | yes | yes | yes | yes | Moderate |
| Khodavandi, 2021^36^ | yes | partial yes | yes | partial yes | unknown | yes | yes | yes | partial yes | no | yes | yes | yes | yes | no | yes | Low |
| Aune, 2015^38^ | yes | partial yes | yes | no | no | yes | partial yes | yes | no | no | yes | no | no | yes | yes | yes | Critically low |
| Zhao, 2022^39^ | yes | partial yes | yes | partial yes | yes | yes | partial yes | yes | partial yes | no | yes | yes | yes | yes | yes | yes | High |
| Arafa, 2021^40^ | yes | partial yes | yes | partial yes | unknown | unknown | partial yes | yes | partial yes | no | yes | yes | yes | yes | yes | yes | Moderate |
| Wang, 2016^41^ | yes | partial yes | yes | partial yes | yes | yes | partial yes | partial yes | no | no | yes | no | no | yes | yes | yes | Critically low |
| Guo, 2021^42^ | yes | yes | yes | partial yes | yes | yes | partial yes | yes | yes | no | yes | yes | yes | yes | yes | yes | High |
| Aune, 2013^43^ | yes | partial yes | yes | no | unknown | unknown | partial yes | yes | no | no | yes | no | no | yes | yes | yes | Critically low |
| Fan, 2019^44^ | yes | partial yes | yes | partial yes | unknown | yes | partial yes | partial yes | partial yes | no | yes | yes | yes | yes | yes | yes | Moderate |
| Gao, 2013^45^ | yes | partial yes | yes | partial yes | yes | yes | partial yes | yes | partial yes | no | yes | yes | yes | yes | yes | yes | High |
| Gijsbers, 2016^46^ | yes | partial yes | yes | partial yes | unknown | unknown | partial yes | partial yes | partial yes | no | yes | yes | no | yes | yes | yes | Low |
| Khoramdad, 2017^47^ | yes | partial yes | yes | partial yes | yes | unknown | partial yes | partial yes | partial yes | no | yes | yes | yes | yes | yes | yes | Moderate |
| Jin, 2021^48^ | yes | partial yes | yes | partial yes | no | yes | partial yes | yes | no | no | yes | no | no | yes | yes | yes | Critically low |
| Babio, 2022^49^ | yes | yes | yes | partial yes | yes | yes | partial yes | yes | partial yes | no | yes | yes | no | no | no | yes | Critically low |
| Ong, 2020^50^ | yes | yes | yes | partial yes | yes | yes | partial yes | yes | partial yes | no | yes | yes | yes | yes | no | yes | Low |
| Matía-Martín, 2019^51^ | yes | yes | yes | partial yes | yes | yes | partial yes | yes | partial yes | no | yes | yes | yes | yes | yes | yes | High |
| Bian, 2018^52^ | yes | partial yes | yes | partial yes | yes | yes | partial yes | yes | partial yes | no | yes | yes | yes | yes | yes | yes | High |
| Hidayat, 2020^53^ | yes | partial yes | yes | partial yes | yes | yes | partial yes | yes | partial yes | no | yes | yes | yes | yes | yes | yes | High |
| Jiang, 2014^54^ | yes | partial yes | yes | partial yes | unknown | unknown | partial yes | yes | no | no | yes | no | no | yes | yes | yes | Critically low |

*, critical items.

#, non-critical items.

#1. PICO: Research questions and criteria included PICO

*2. Protocol: Protocol registered before commencement of the review

*7. Excluded studies: Justification for excluding individual studies

*11. Meta-analytical methods: Appropriateness of meta-analytical methods

^1^ Quality class-

High: No or one non-critical weakness: the systematic review provides an accurate and comprehensive summary of the results of the available studies that address the question of interest;

Moderate: More than one non-critical weakness**: the systematic review has more than one weakness but no critical flaws. It may provide an accurate summary of the results of the available studies that were included in the review;

Low: One critical flaw with or without non-critical weaknesses: the review has a critical flaw and may not provide an accurate and comprehensive summary of the available studies that address the question of interest;

Critically low: More than one critical flaw with or without non-critical weaknesses: the review has more than one critical flaw and should not be relied on to provide an accurate and comprehensive summary of the available studies.

**Multiple non-critical weaknesses may diminish confidence in the review and it may be appropriate to move the overall appraisal down from moderate to low confidence.

# Supplementary Table 13. Subgroup analyses of the association between cheese consumption (highest vs. lowest intake level) and all-cause and cause-specific mortality according to whether adjusting for total energy intake in the models

| **Subgroup** | **Cohort comparisons, n** | **Cases, n** | **Participants, n** | **RR (95%CI)** | ***P* value** | ***I*^2^** | ***P*-heterogeneity** | ***P*-subgroup** |
| --- | --- | --- | --- | --- | --- | --- | --- | --- |
| **All-cause mortality** |  |  |  |  |  |  |  |  |
| All-cause mortality |  |  |  |  |  |  |  | 0.15 |
| Adjusted | 18 | 134,475 | 1,019,279 | 0.95 (0.92, 0.98) | 0.0027 | 39% | 0.05 |  |
| Unadjusted | 3 | 1,823 | >10,964 | 1.06 (0.92, 1.23) | 0.4191 | 0% | 0.88 |  |
| **Cancer mortality** |  |  |  |  |  |  |  |  |
| Overall cancer mortality |  |  |  |  |  |  |  | 0.84 |
| Adjusted | 12 | 28,781 | 801,128 | 1.00 (0.97, 1.03) | 0.9376 | 0% | 0.44 |  |
| Unadjusted | 13 | 1,970 | 577,737 | 0.98 (0.83, 1.16) | 0.8530 | 13% | 0.32 |  |
| Colorectal cancer mortality |  |  |  |  |  |  |  | 0.48 |
| Adjusted | 2 | 401 | 2,257 | 0.85 (0.40, 1.81) | 0.6747 | 81% | 0.02 |  |
| Unadjusted | 2 | 350 | 158,810 | 1.13 (0.87, 1.46) | 0.3481 | 0% | 0.76 |  |
| Colon cancer mortality |  |  |  |  |  |  |  | — |
| Adjusted | 0 | — | — | — | — | — | — |  |
| Unadjusted | 3 | 236 | 133,317 | 1.18 (0.73, 1.89) | 0.5050 | 53% | 0.12 |  |
| Rectal cancer mortality |  |  |  |  |  |  |  | — |
| Adjusted | 0 | — | — | — | — | — | — |  |
| Unadjusted | 2 | 114 | 133,317 | 1.12 (0.73, 1.73) | 0.6007 | 0% | 0.71 |  |
| Prostate cancer mortality |  |  |  |  |  |  |  | 0.70 |
| Adjusted | 1 | 178 | 293,888 | 1.24 (0.56, 2.75) | 0.5962 | — | — |  |
| Unadjusted | 1 | 97 | 6,763 | 1.50 (0.88, 2.55) | 0.1341 | — | — |  |
| Lung cancer mortality |  |  |  |  |  |  |  | 0.31 |
| Adjusted | 1 | NA | 217,755 | 1.01 (0.80, 1.28) | 0.9339 | — | — |  |
| Unadjusted | 5 | 781 | 121,601 | 0.79 (0.52, 1.20) | 0.2643 | 33% | 0.20 |  |
| Pancreatic cancer mortality |  |  |  |  |  |  |  | 0.87 |
| Adjusted | 3 | NA | 217,755 | 1.26 (0.88, 1.81) | 0.2090 | NA | NA |  |
| Unadjusted | 1 | 25 | 3158 | 1.14 (0.35, 3.68) | 0.8259 | — | — |  |
| Breast cancer mortality |  |  |  |  |  |  |  | 0.63 |
| Adjusted | 2 | NA | 168,153 | 1.21 (0.88, 1.67) | 0.2435 | NA | NA |  |
| Unadjusted | 1 | 142 | 994 | 1.04 (0.61, 1.75) | 0.8840 | — | — |  |
| Ovarian cancer mortality |  |  |  |  |  |  |  | 0.49 |
| Adjusted | 2 | NA | 168,153 | 1.15 (0.71, 1.85) | 0.5673 | NA | NA |  |
| Unadjusted | 1 | 77 | 64,327 | 1.66 (0.65, 4.25) | 0.2900 | — | — |  |
| Gastric cancer mortality |  |  |  |  |  |  |  | — |
| Adjusted | 0 | — | — | — | — | — | — |  |
| Unadjusted | 2 | 910 | 113,950 | 1.02 (0.66, 1.58) | 0.9337 | 0% | 0.61 |  |
| Urothelial cancer mortality |  |  |  |  |  |  |  | — |
| Adjusted | 0 | — | — | — | — | — | — |  |
| Unadjusted | 1 | 46 | 114,517 | 0.91 (0.46, 1.82) | NA | — | — |  |
| **Cardiovascular mortality** |  |  |  |  |  |  |  |  |
| Overall cardiovascular mortality |  |  |  |  |  |  |  | 0.27 |
| Adjusted | 14 | 36,537 | 730,783 | 0.92 (0.87, 0.97) | 0.0034 | 18% | 0.26 |  |
| Unadjusted | 2 | 428 | 11,788 | 1.43 (0.65, 3.11) | 0.3712 | 67% | 0.08 |  |
| Coronary heart disease mortality |  |  |  |  |  |  |  | 0.29 |
| Adjusted | 5 | 4,415 | 222,138 | 0.93 (0.79, 1.10) | 0.3954 | 51% | 0.08 |  |
| Unadjusted | 2 | 428 | 11,788 | 1.43 (0.65, 3.11) | 0.3712 | 67% | 0.08 |  |
| Stroke mortality |  |  |  |  |  |  |  | — |
| Adjusted | 4 | 1,508 | 197,664 | 0.76 (0.58, 1.01) | 0.0544 | 42% | 0.16 |  |
| Unadjusted | 0 | — | — | — | — | — | — |  |

**Abbreviation：** NA= not available; RR= relative risk.

# Supplementary Table 14. Subgroup analyses of the association between cheese consumption (highest vs. lowest intake level) and disease risk according to whether adjusting for total energy intake in the models

| **Subgroup** | **Cohort comparisons, n** | **Cases, n** | **Participants, n** | **RR (95%CI)** | ***P* value** | ***I*^2^** | ***P*-heterogeneity** | ***P*-subgroup** |
| --- | --- | --- | --- | --- | --- | --- | --- | --- |
| **Cardiovascular disease** |  |  |  |  |  |  |  |  |
| Overall CVD |  |  |  |  |  |  |  | 0.27 |
| Adjusted | 13 | 47,612 | 550,678 | 0.93 (0.89, 0.98) | 0.0039 | 37% | 0.08 |  |
| Unadjusted | 5 | 39,184 | 1,282,434 | 0.84 (0.70, 1.01) | 0.0618 | 29% | 0.23 |  |
| Coronary heart disease |  |  |  |  |  |  |  | 0.60 |
| Adjusted | 9 | 758 | 2,759 | 0.92 (0.86, 0.99) | 0.0172 | 32% | 0.16 |  |
| Unadjusted | 3 | 16,810 | 683,814 | 0.82 (0.54, 1.24) | 0.3527 | 34% | 0.22 |  |
| Stroke |  |  |  |  |  |  |  | 0.09 |
| Adjusted | 8 | 20,448 | 727,872 | 0.94 (0.89, 0.98) | 0.0055 | 0% | 0.74 |  |
| Unadjusted | 1 | 690 | 85,764 | 0.63 (0.40, 0.99) | 0.0457 | — | — |  |
| Hypertension |  |  |  |  |  |  |  | 0.10 |
| Adjusted | 10 | >103,698 | 294,230 | 0.98 (0.92, 1.03) | 0.4251 | 49% | 0.04 |  |
| Unadjusted | 1 | NA | 462,155 | 0.83 (0.69, 1.00) | 0.0490 | — | — |  |
| **Cancer** |  |  |  |  |  |  |  |  |
| Overall cancer |  |  |  |  |  |  |  | 0.02 |
| Adjusted | 42 | 155,132 | 9,044,713 | 0.99 (0.97, 1.01) | 0.1654 | 17% | 0.17 |  |
| Unadjusted | 4 | 3,469 | 494,990 | 1.14 (1.01, 1.29) | 0.0305 | 0% | 0.57 |  |
| Prostate cancer |  |  |  |  |  |  |  | 0.34 |
| Adjusted | 11 | 40,204 | 1,213,987 | 1.03 (0.98, 1.08) | 0.2913 | 0% | 0.58 |  |
| Unadjusted | 1 | 642 | 1,525 | 1.21 (0.87, 1.69) | 0.2647 | — | — |  |
| Advanced prostate cancer |  |  |  |  |  |  |  | — |
| Adjusted | 2 | 3,774 | 456,704 | 0.87 (0.67, 1.13) | 0.2967 | 54% | 0.14 |  |
| Unadjusted | 0 | — | — | — | — | — | — |  |
| Colorectal cancer |  |  |  |  |  |  |  | 0.33 |
| Adjusted | 8 | 14,362 | 1,271,354 | 1.00 (0.89, 1.13) | 0.9942 | 49% | 0.06 |  |
| Unadjusted | 2 | 2,624 | 473,124 | 1.10 (0.95, 1.26) | 0.1935 | 0% | 0.76 |  |
| Colon cancer |  |  |  |  |  |  |  | — |
| Adjusted | 6 | 7,381 | 779,173 | 0.95 (0.80, 1.12) | 0.5127 | 59% | 0.03 |  |
| Unadjusted | 0 | — | — | — | — | — | — |  |
| Proximal colon cancer |  |  |  |  |  |  |  | — |
| Adjusted | 3 | 2,927 | 1,056,964 | 0.91 (0.63, 1.31) | 0.6065 | 83% | <0.01 |  |
| Unadjusted | 0 | — | — | — | — | — | — |  |
| Distal colon cancer |  |  |  |  |  |  |  | — |
| Adjusted | 3 | 2,635 | 1,056,964 | 0.97 (0.84, 1.14) | 0.7394 | 0% | 0.71 |  |
| Unadjusted | 0 | — | — | — | — | — | — |  |
| Rectal cancer |  |  |  |  |  |  |  | — |
| Adjusted | 6 | 3,624 | 779,173 | 1.02 (0.89, 1.17) | 0.7692 | 0% | 0.84 |  |
| Unadjusted | 0 | — | — | — | — | — | — |  |
| Breast cancer |  |  |  |  |  |  |  | 0.04 |
| Adjusted | 8 | 74,032 | 2,293,553 | 0.98 (0.96, 1.01) | 0.1232 | 35% | 0.15 |  |
| Unadjusted | 1 | 203 | 20,341 | 1.43 (0.99, 2.06) | 0.0557 | — | — |  |
| Estrogen-receptor positive breast cancer |  |  |  |  |  |  |  | — |
| Adjusted | 2 | 31,952 | 1,833,420 | 0.99 (0.95, 1.02) | 0.4108 | 0% | 0.78 |  |
| Unadjusted | 0 | — | — | — | — | — | — |  |
| Estrogen-receptor-negative breast cancer |  |  |  |  |  |  |  | — |
| Adjusted | 2 | 6,866 | 1,833,420 | 0.89 (0.82, 0.97) | 0.0059 | 0% | 0.44 |  |
| Unadjusted | 0 | — | — | — | — | — | — |  |
| Bladder cancer |  |  |  |  |  |  |  | — |
| Adjusted | 3 | 4,514 | 780,950 | 0.94 (0.85, 1.04) | 0.2411 | 0% | 0.44 |  |
| Unadjusted | 0 | — | — | — | — | — | — |  |
| Pancreatic cancer |  |  |  |  |  |  |  | — |
| Adjusted | 3 | 2,464 | 960,598 | 1.05 (0.86, 1.30) | 0.6143 | 0% | 0.85 |  |
| Unadjusted | 0 | — | — | — | — | — | — |  |
| Endometrial cancer |  |  |  |  |  |  |  | — |
| Adjusted | 2 | 2,707 | 506,970 | 0.91 (0.77, 1.07) | 0.2354 | 37% | 0.21 |  |
| Unadjusted | 0 | — | — | — | — | — | — |  |
| Hepatocellular carcinoma |  |  |  |  |  |  |  | — |
| Adjusted | 2 | 355 | 622,051 | 1.17 (0.67, 2.04) | 0.5911 | 73% | 0.05 |  |
| Unadjusted | 0 | — | — | — | — | — | — |  |
| Ovarian cancer |  |  |  |  |  |  |  | — |
| Adjusted | 2 | 2,608 | 878,948 | 1.11 (0.91, 1.35) | 0.3054 | 0% | 0.75 |  |
| Unadjusted | 0 | — | — | — | — | — | — |  |
| Lymphoma |  |  |  |  |  |  |  | — |
| Adjusted | 1 | 1,334 | 410,411 | 1.10 (0.86, 1.39) | 0.4491 | — | — |  |
| Unadjusted | 0 | — | — | — | — | — | — |  |
| Precursors of colorectal cancer |  |  |  |  |  |  |  | — |
| Adjusted | 2 | 1,197 | 23,745 | 0.99 (0.81, 1.22) | 0.9405 | 27% | 0.24 |  |
| Unadjusted | 0 | — | — | — | — | — | — |  |
| **Metabolic disease** |  |  |  |  |  |  |  |  |
| Type 2 diabetes |  |  |  |  |  |  |  | 0.44 |
| Adjusted | 23 | 43,559 | 662,289 | 0.93 (0.88, 0.99) | 0.0228 | 48% | <0.01 |  |
| Unadjusted | 2 | 1,025 | 11,818 | 0.87 (0.73, 1.03) | 0.098 | 0% | 0.69 |  |
| Prediabetes |  |  |  |  |  |  |  | — |
| Adjusted | 3 | 2,851 | 10,899 | 0.93 (0.78, 1.12) | 0.4522 | 66% | 0.05 |  |
| Unadjusted | 0 | — | — | — | — | — | — |  |
| Metabolic syndrome |  |  |  |  |  |  |  | 0.03 |
| Adjusted | 3 | >982 | 3,525 | 1.21 (0.96, 1.52) | 0.1017 | 0% | 0.4 |  |
| Unadjusted | 1 | 648 | 3,417 | 0.84 (0.67, 1.06) | 0.1363 | — | — |  |
| Overweight/obesity |  |  |  |  |  |  |  | 0.38 |
| Adjusted | 1 | 8,238 | 18,295 | 1.02 (0.89,1.16) | 0.7673 | — | — |  |
| Unadjusted | 1 | NA | 7,356 | 0.91 (0.73,1.13) | 0.3975 | — | — |  |
| **Aging-related disease** |  |  |  |  |  |  |  |  |
| Total fracture |  |  |  |  |  |  |  | 0.23 |
| Adjusted | 6 | 25,715 | 232,924 | 0.90 (0.85, 0.94) | <0.0001 | 0% | 0.49 |  |
| Unadjusted | 1 | 686 | 73,715 | 1.08 (0.81, 1.46) | 0.6086 | — | — |  |
| Hip fracture |  |  |  |  |  |  |  | 0.14 |
| Adjusted | 6 | 8,411 | 232,924 | 0.83 (0.68, 1.00) | 0.0545 | 69% | <0.01 |  |
| Unadjusted | 1 | 686 | 73,715 | 1.08 (0.81, 1.46) | 0.6086 | — | — |  |
| Fall |  |  |  |  |  |  |  | — |
| Adjusted | 2 | 1,002 | 11,908 | 0.96 (0.82, 1.12) | 0.5920 | 0% | 0.47 |  |
| Unadjusted | 0 | — | — | — | — | — | — |  |
| Frailty |  |  |  |  |  |  |  | 0.44 |
| Adjusted | 1 | 134 | 1,871 | 0.91 (0.52, 1.60) | 0.7436 | — | — |  |
| Unadjusted | 1 | 150 | 823 | 1.25 (0.71, 2.21) | 0.4411 | — | — |  |
| Dementia |  |  |  |  |  |  |  | 0.37 |
| Adjusted | 1 | 337 | 2,497 | 0.72 (0.52, 0.99) | 0.0455 | — | — |  |
| Unadjusted | 1 | 4,282 | 249,511 | 0.87 (0.67, 1.13) | 0.2963 | — | — |  |
| Parkinson’s disease |  |  |  |  |  |  |  | — |
| Adjusted | 5 | 955 | 296,689 | 1.26 (0.99, 1.60) | 0.0516 | 29% | 0.23 |  |
| Unadjusted | 0 | — | — | — | — | — | — |  |

**Abbreviation:** CVD= cardiovascular disease; NA= not available; RR= relative risk.

.

# Supplementary Table 15. Subgroup analyses of the association between cheese consumption (highest vs. lowest intake level) and all-cause and cause-specific mortality according to geographic locations

| **Subgroup** | **Cohort comparisons, n** | **Cases, n** | **Participants, n** | **RR (95%CI)** | ***P* value** | ***I*^2^** | ***P*-heterogeneity** | ***P*-subgroup** |
| --- | --- | --- | --- | --- | --- | --- | --- | --- |
| **All-cause mortality** |  |  |  |  |  |  |  |  |
| All-cause mortality |  |  |  |  |  |  |  | 0.52 |
| Europe, North America, Oceania | 17 | 121,276 | >817,295 | 0.96 (0.93, 0.99) | 0.0215 | 33% | 0.09 |  |
| Asia and other regions | 3 | 10,167 | 76,564 | 0.92 (0.80, 1.06) | 0.2580 | 60% | 0.08 |  |
| Multi-region | 1 | 4,855 | 136,384 | 0.87 (0.72, 1.05) | 0.1479 | — | — |  |
| **Cancer mortality** |  |  |  |  |  |  |  |  |
| Overall cancer mortality |  |  |  |  |  |  |  | 0.75 |
| Europe, North America, Oceania | 13 | 25,942 | 803,502 | 1.01 (0.97, 1.04) | 0.7647 | 22% | 0.22 |  |
| Asia and other regions | 12 | 4,876 | 575,430 | 0.99 (0.87, 1.11) | 0.8106 | 0% | 0.62 |  |
| Colorectal cancer mortality |  |  |  |  |  |  |  | 0.44 |
| Europe, North America, Oceania | 3 | 576 | 53,243 | 0.94 (0.61, 1.45) | 0.7838 | 66% | 0.06 |  |
| Asia and other regions | 1 | 175 | 107,824 | 1.18 (0.81, 1.72) | 0.3889 | — | — |  |
| Colon cancer mortality |  |  |  |  |  |  |  | 0.96 |
| Europe, North America, Oceania | 2 | 142 | 25,493 | 1.21 (0.52, 2.81) | 0.6642 | 77% | 0.04 |  |
| Asia and other regions | 1 | 94 | 107,824 | 1.17 (0.68, 2.01) | 0.5701 | — | — |  |
| Rectal cancer mortality |  |  |  |  |  |  |  | — |
| Europe, North America, Oceania | 2 | 114 | 133,317 | 1.12 (0.73, 1.73) | 0.6007 | 0 | 0.71 |  |
| Asia and other regions | 0 | — | — | — | — | — | — |  |
| Prostate cancer mortality |  |  |  |  |  |  |  | — |
| Europe, North America, Oceania | 2 | 275 | 300,651 | 1.41 (0.91, 2.20) | 0.1234 | 0 | 0.70 |  |
| Asia and other regions | 0 | — | — | — | — | — | — |  |
| Lung cancer mortality |  |  |  |  |  |  |  | 0.95 |
| Europe, North America, Oceania | 2 | >158 | 237,950 | 0.85 (0.53, 1.38) | 0.5148 | 54% | 0.14 |  |
| Asia and other regions | 4 | 623 | 101,406 | 0.87 (0.51, 1.50) | 0.6286 | 45% | 0.14 |  |
| Pancreatic cancer mortality |  |  |  |  |  |  |  | — |
| Europe, North America, Oceania | 4 | >25 | 220,913 | 1.25 (0.89, 1.76) | 0.2057 | 0% | 0.69 |  |
| Asia and other regions | 0 | — | — | — | — | — | — |  |
| Breast cancer mortality |  |  |  |  |  |  |  | — |
| Europe, North America, Oceania | 3 | >142 | 169,147 | 1.16 (0.88, 1.53) | 0.2835 | 0% | 0.63 |  |
| Asia and other regions | 0 | — | — | — | — | — | — |  |
| Ovarian cancer mortality |  |  |  |  |  |  |  | — |
| Europe, North America, Oceania | 3 | >77 | 232,480 | 1.24 (0.81, 1.90) | 0.3220 | 0% | 0.50 |  |
| Asia and other regions | 0 | — | — | — | — | — | — |  |
| Gastric cancer mortality |  |  |  |  |  |  |  | — |
| Europe, North America, Oceania | 2 | 910 | 113,950 | 1.02 (0.66, 1.58) | 0.9337 | 0% | 0.61 |  |
| Asia and other regions | 0 | — | — | — | — | — | — |  |
| Urothelial cancer mortality |  |  |  |  |  |  |  | — |
| Europe, North America, Oceania | 1 | 46 | 114,517 | 0.91 (0.46, 1.82) | NA | — | — |  |
| Asia and other regions | 0 | — | — | — | — | — | — |  |
| **Cardiovascular mortality** |  |  |  |  |  |  |  |  |
| Overall cardiovascular mortality |  |  |  |  |  |  |  | 0.17 |
| Europe, North America, Oceania | 12 | 29,182 | 529,623 | 0.94 (0.89, 1.00) | 0.0614 | 35% | 0.11 |  |
| Asia and other regions | 3 | 3,160 | 76,564 | 0.88 (0.70, 1.10) | 0.2507 | 49% | 0.14 |  |
| Multi-region | 1 | 4,623 | 136,384 | 0.92 (0.77, 1.10) | 0.3715 | — | — |  |
| Coronary heart disease mortality |  |  |  |  |  |  |  | 0.03 |
| Europe, North America, Oceania | 6 | 4,079 | 191,523 | 1.04 (0.96, 1.13) | 0.3450 | 0% | 0.57 |  |
| Asia and other regions | 1 | 764 | 42,403 | 0.67 (0.51, 0.89) | 0.0048 | — | — |  |
| Stroke mortality |  |  |  |  |  |  |  | 0.90 |
| Europe, North America, Oceania | 3 | 1,001 | 155,261 | 0.77 (0.51, 1.15) | 0.2016 | 61% | 0.08 |  |
| Asia and other regions | 1 | 507 | 42,403 | 0.74 (0.53, 1.04) | 0.0799 | — | — |  |

**Abbreviation:** RR= relative risk.

*Multi-region: including population from Europe/North America/Australia and Asia as well as other regions simultaneously.

# Supplementary Table 16. Subgroup analyses of the association between cheese consumption (highest vs. lowest intake level) and disease risk according to geographic locations

| **Subgroup** | **Cohort comparisons, n** | **Cases, n** | **Participants, n** | **RR (95%CI)** | ***P* value** | ***I*^2^** | ***P*-heterogeneity** | ***P*-subgroup** |
| --- | --- | --- | --- | --- | --- | --- | --- | --- |
| **Cardiovascular disease** |  |  |  |  |  |  |  |  |
| Overall CVD |  |  |  |  |  |  |  | — |
| Europe, North America, Oceania | 18 | 86,796 | 1,833,112 | 0.92 (0.89, 0.96) | 0.0001 | 38% | 0.05 |  |
| Asia and other regions | 0 | — | — | — | — | — | — |  |
| Coronary heart disease |  |  |  |  |  |  |  | — |
| Europe, North America, Oceania | 12 | 17,568 | 686,573 | 0.92 (0.86, 0.98) | 0.0108 | 27% | 0.17 |  |
| Asia and other regions | 0 | — | — | — | — | — | — |  |
| Stroke |  |  |  |  |  |  |  | — |
| Europe, North America, Oceania | 9 | 21,138 | 813,636 | 0.93 (0.89, 0.98) | 0.0030 | 0% | 0.52 |  |
| Asia and other regions | 0 | — | — | — | — | — | — |  |
| Hypertension |  |  |  |  |  |  |  | 0.71 |
| Europe, North America, Oceania | 9 | >103,343 | 755,298 | 0.96 (0.91, 1.01) | 0.1233 | 37% | 0.12 |  |
| Asia and other regions | 2 | 355 | 1,087 | 1.15 (0.46, 2.86) | 0.7719 | 81% | 0.02 |  |
| **Cancer** |  |  |  |  |  |  |  |  |
| Overall cancer |  |  |  |  |  |  |  | 0.40 |
| Europe, North America, Oceania | 41 | 120,028 | 8,236,094 | 0.99 (0.97, 1.01) | 0.5022 | 26% | 0.07 |  |
| Asia and other regions | 4 | 993 | 161,760 | 1.16 (0.90, 1.49) | 0.2558 | 0% | 0.72 |  |
| Multi-region | 1 | 37,580 | 1,141,849 | 0.98 (0.95, 1.02) | 0.2702 |  |  |  |
| Prostate cancer |  |  |  |  |  |  |  | 0.23 |
| Europe, North America, Oceania | 10 | 40,105 | 1,151,670 | 1.02 (0.98, 1.07) | 0.3291 | 0% | 0.59 |  |
| Asia and other regions | 2 | 741 | 63,842 | 1.24 (0.91, 1.68) | 0.1718 | 0% | 0.48 |  |
| Advanced prostate cancer |  |  |  |  |  |  |  | — |
| Europe, North America, Oceania | 2 | 3,774 | 456,704 | 0.87 (0.67, 1.13) | 0.2967 | 54% | 0.14 |  |
| Asia and other regions | 0 | — | — | — | — | — | — |  |
| Colorectal cancer |  |  |  |  |  |  |  | — |
| Europe, North America, Oceania | 10 | 16,986 | 1,744,478 | 1.02 (0.92, 1.13) | 0.7581 | 41% | 0.08 |  |
| Asia and other regions | 0 | — | — | — | — | — | — |  |
| Colon cancer |  |  |  |  |  |  |  | — |
| Europe, North America, Oceania | 6 | 7,381 | 779,173 | 0.95 (0.80, 1.12) | 0.5127 | 59% | 0.03 |  |
| Asia and other regions | 0 | — | — | — | — | — | — |  |
| Proximal colon cancer |  |  |  |  |  |  |  | — |
| Europe, North America, Oceania | 3 | 2,927 | 1,056,964 | 0.91 (0.63, 1.31) | 0.6065 | 83% | <0.01 |  |
| Asia and other regions | 0 | — | — | — | — | — | — |  |
| Distal colon cancer |  |  |  |  |  |  |  | — |
| Europe, North America, Oceania | 3 | 2,635 | 1,056,964 | 0.97 (0.84, 1.14) | 0.7394 | 0% | 0.71 |  |
| Asia and other regions | 0 | — | — | — | — | — | — |  |
| Rectal cancer |  |  |  |  |  |  |  | — |
| Europe, North America, Oceania | 6 | 3,624 | 779,173 | 1.02 (0.89, 1.17) | 0.7692 | 0% | 0.84 |  |
| Asia and other regions | 0 | — | — | — | — | — | — |  |
| Breast cancer |  |  |  |  |  |  |  | 0.92 |
| Europe, North America, Oceania | 8 | 36,655 | 1,172,045 | 0.99 (0.90, 1.08) | 0.7562 | 52% | 0.04 |  |
| Multi-region | 1 | 37,580 | 1,141,849 | 0.98 (0.95, 1.02) | 0.2702 | — | — |  |
| Estrogen-receptor positive breast cancer |  |  |  |  |  |  |  | 0.40 |
| Europe, North America, Oceania | 1 | 9,912 | 691,571 | 0.99 (0.94**,** 1.04) | 0.6968 | — | — |  |
| Multi-region | 1 | 22,040 | 1,141,849 | 0.98 (0.93, 1.03) | 0.4381 | — | — |  |
| Estrogen-receptor-negative breast cancer |  |  |  |  |  |  |  | 0.44 |
| Europe, North America, Oceania | 1 | 1,499 | 691,571 | 0.93 (0.81, 1.06) | 0.2902 | — | — |  |
| Multi-region | 1 | 5,367 | 1,141,849 | 0.87 (0.79, 0.97) | 0.0078 | — | — |  |
| Bladder cancer |  |  |  |  |  |  |  | — |
| Europe, North America, Oceania | 3 | 4,514 | 780,950 | 0.94 (0.85, 1.04) | 0.2411 | 0% | 0.44 |  |
| Asia and other regions | 0 | — | — | — | — | — | — |  |
| Pancreatic cancer |  |  |  |  |  |  |  | 0.80 |
| Europe, North America, Oceania | 1 | 2,212 | 862,680 | 1.07 (0.85, 1.36) | 0.5726 | — | — |  |
| Asia and other regions | 2 | 252 | 97,918 | 1.00 (0.64, 1.57) | 0.9930 | 0% | 0.6 |  |
| Endometrial cancer |  |  |  |  |  |  |  | — |
| Europe, North America, Oceania | 2 | 2,707 | 506,970 | 0.91 (0.77, 1.07) | 0.2354 | 37% | 0.21 |  |
| Asia and other regions | 0 | — | — | — | — | — | — |  |
| Hepatocellular carcinoma |  |  |  |  |  |  |  | — |
| Europe, North America, Oceania | 2 | 355 | 622,051 | 1.17 (0.67, 2.04) | 0.5911 | 73% | 0.05 |  |
| Asia and other regions | 0 | — | — | — | — | — | — |  |
| Ovarian cancer |  |  |  |  |  |  |  | — |
| Europe, North America, Oceania | 2 | 2,608 | 878,948 | 1.11 (0.91, 1.35) | 0.3054 | 0% | 0.75 |  |
| Asia and other regions | 0 | — | — | — | — | — | — |  |
| Lymphoma |  |  |  |  |  |  |  | — |
| Europe, North America, Oceania | 1 | 1,334 | 410,411 | 1.10 (0.86, 1.39) | 0.4491 | — | — |  |
| Asia and other regions | 0 | — | — | — | — | — | — |  |
| Precursors of colorectal cancer |  |  |  |  |  |  |  | — |
| Europe, North America, Oceania | 2 | 1,197 | 23,745 | 0.99 (0.81, 1.22) | 0.9405 | 27% | 0.24 |  |
| Asia and other regions | 0 | — | — | — | — | — | — |  |
| **Metabolic disease** |  |  |  |  |  |  |  |  |
| Type 2 diabetes |  |  |  |  |  |  |  | 0.75 |
| Europe, North America, Oceania | 21 | 41,090 | 561,023 | 0.93 (0.88, 0.99) | 0.0213 | 48% | <0.01 |  |
| Asia and other regions | 4 | 3,494 | 113,084 | 0.90 (0.76, 1.07) | 0.2527 | 40% | 0.17 |  |
| Prediabetes |  |  |  |  |  |  |  | — |
| Western countries | 3 | 2,851 | 10,899 | 0.93 (0.78, 1.12) | 0.4522 | 66% | 0.05 |  |
| Asian countries | 0 | — | — | — | — | — | — |  |
| Metabolic syndrome |  |  |  |  |  |  |  | 0.91 |
| Europe, North America, Oceania | 3 | >1,578 | 6,411 | 1.03 (0.75, 1.41) | 0.8688 | 79% | <0.01 |  |
| Asia and other regions | 1 | 52 | 531 | 1.07 (0.54, 2.11) | 0.8457 | — | — |  |
| Overweight/obesity |  |  |  |  |  |  |  | — |
| Europe, North America, Oceania | 2 | > 8,238 | 25,651 | 0.99 (0.88, 1.11) | 0.8556 | 0% | 0.38 |  |
| Asia and other regions | 0 | — | — | — | — | — | — |  |
| **Aging-related disease** |  |  |  |  |  |  |  |  |
| Total fracture |  |  |  |  |  |  |  | — |
| Europe, North America, Oceania | 7 | 26,401 | 306,639 | 0.90 (0.86, 0.95) | <0.0001 | 0% | 0.44 |  |
| Asia and other regions | 0 | — | — | — | — | — | — |  |
| Hip fracture |  |  |  |  |  |  |  | — |
| Europe, North America, Oceania | 7 | 9,097 | 306,639 | 0.86 (0.72, 1.04) | 0.1127 | 71% | <0.01 |  |
| Asia and other regions | 0 | — | — | — | — | — | — |  |
| Fall |  |  |  |  |  |  |  | — |
| Europe, North America, Oceania | 2 | 1,002 | 11,908 | 0.96 (0.82, 1.12) | 0.5920 | 0% | 0.47 |  |
| Asia and other regions | 0 | — | — | — | — | — | — |  |
| Frailty |  |  |  |  |  |  |  | — |
| Europe, North America, Oceania | 2 | 284 | 2,694 | 1.07 (0.71, 1.59) | 0.7554 | 0% | 0.44 |  |
| Asia and other regions | 0 | — | — | — | — | — | — |  |
| Dementia |  |  |  |  |  |  |  | — |
| Europe, North America, Oceania | 2 | 4,619 | 252,008 | 0.81 (0.66, 0.99) | 0.0383 | 0% | 0.37 |  |
| Asia and other regions | 0 | — | — | — | — | — | — |  |
| Parkinson’s disease |  |  |  |  |  |  |  | — |
| Europe, North America, Oceania | 5 | 955 | 296,689 | 1.26 (0.99, 1.60) | 0.0516 | 29% | 0.23 |  |
| Asia and other regions | 0 | — | — | — | — | — | — |  |

**Abbreviation:** CVD= cardiovascular disease; RR= relative risk.

*, Multi-region: including population from Europe/North America/Australia and Asia as well as other regions simultaneously.

# Supplementary Table 17. Scoring for the different components of NutriGrade for each health outcome.

| Health outcome | Risk of bias | Precision | Heterogeneity | Directness | Publication bias | Funding bias | Effect size | Dose-response | Total score | Evidence class of NutriGrade |
| --- | --- | --- | --- | --- | --- | --- | --- | --- | --- | --- |
| **Mortality** |  |  |  |  |  |  |  |  |  |  |
| All-cause | 2 | 1 | 1 | 1 | 1 | 0 | 0 | 1 | 7 | Moderate |
| Overall cancer | 2 | 1 | 1 | 1 | 1 | 0 | 0 | 0 | 6 | Moderate |
| Colorectal cancer | 2 | 0 | 0 | 1 | 0 | 1 | 0 | 0 | 4 | Low |
| Colon cancer | 2 | 0 | 0 | 1 | 0 | 1 | 0 | 0 | 4 | Low |
| Rectal cancer | 2 | 0 | 0 | 1 | 0 | 1 | 0 | 0 | 4 | Low |
| Prostate cancer | 2 | 0 | 0 | 1 | 0 | 0.5 | 0 | 0 | 3.5 | Very low |
| Lung cancer | 2 | 0 | 0 | 1 | 0 | 1 | 0 | 0 | 4 | Low |
| Pancreatic cancer | 2 | 0 | 0 | 1 | 0 | 0.5 | 0 | 0 | 3.5 | Very low |
| Breast cancer | 2 | 0 | 0 | 1 | 0 | 0.5 | 0 | 0 | 3.5 | Very low |
| Ovarian cancer | 2 | 0 | 0 | 1 | 0 | 0.5 | 0 | 0 | 3.5 | Very low |
| Gastric cancer | 2 | 0 | 0 | 1 | 0 | 1 | 0 | 0 | 4 | Low |
| CVD | 2 | 1 | 1 | 1 | 1 | 0 | 0 | 1 | 7 | Moderate |
| CHD | 2 | 1 | 0.4 | 1 | 0.5 | 0 | 0 | 0 | 4.9 | Low |
| Stroke | 2 | 0 | 0 | 1 | 0 | 0 | 0 | 0 | 3 | Very low |
| **Cardiovascular disease** | | |  |  |  |  |  |  |  |  |
| Overall CVD | 2 | 1 | 1 | 1 | 1 | 0 | 0 | 1 | 7 | Moderate |
| Coronary heart disease | 2 | 1 | 1 | 1 | 1 | 0 | 0 | 1 | 7 | Moderate |
| Stroke | 2 | 1 | 0.5 | 1 | 0.5 | 0 | 0 | 1 | 6 | Moderate |
| Hypertension | 2 | 1 | 0.8 | 1 | 1 | 0.5 | 0 | 0 | 6.3 | Moderate |
| **Cancer** |  |  |  |  |  |  |  |  |  |  |
| Overall cancer | 2 | 1 | 1 | 1 | 0.5 | 0 | 0 | 0 | 5.5 | Low |
| Prostate cancer | 2 | 1 | 1 | 1 | 0.5 | 0.5 | 0 | 0 | 6 | Moderate |
| Advanced prostate cancer | 2 | 0 | 0 | 1 | 0 | 0.5 | 0 | 0 | 3.5 | Very low |
| Colorectal cancer | 2 | 1 | 0.8 | 1 | 1 | 0 | 0 | 0 | 5.8 | Low |
| Colon cancer | 2 | 1 | 0.4 | 1 | 0.5 | 0.5 | 0 | 0 | 5.4 | Low |
| Proximal colon cancer | 2 | 0 | 0 | 1 | 0 | 0.5 | 0 | 0 | 3.5 | Very low |
| Distal colon cancer | 2 | 1 | 0 | 1 | 0 | 0.5 | 0 | 0 | 4.5 | Low |
| Rectal cancer | 2 | 1 | 0.5 | 1 | 0.5 | 0.5 | 0 | 0 | 5.5 | Low |
| Breast cancer | 2 | 1 | 0.4 | 1 | 0.5 | 0 | 0 | 0 | 4.9 | Low |
| Estrogen-receptor positive breast cancer | 2 | 1 | 0 | 1 | 0 | 0.5 | 0 | 0 | 4.5 | Low |
| Estrogen-receptor-negative breast cancer | 2 | 1 | 0 | 1 | 0 | 0.5 | 0 | 0 | 4.5 | Low |
| Bladder cancer | 2 | 1 | 0 | 1 | 0 | 0.5 | 0 | 0 | 4.5 | Low |
| Pancreatic cancer | 2 | 0 | 0 | 1 | 0 | 1 | 0 | 0 | 4 | Low |
| Endometrial cancer | 2 | 0 | 0 | 1 | 0 | 0.5 | 0 | 0 | 3.5 | Very low |
| Hepatocellular carcinoma | 2 | 0 | 0 | 1 | 0 | 0 | 0 | 0 | 3 | Very low |
| Ovarian cancer | 1 | 0 | 0 | 1 | 0 | 0.5 | 0 | 0 | 2.5 | Very low |
| Precursors of colorectal cancer | 1 | 0 | 0 | 1 | 0 | 0 | 0 | 0 | 2 | Very low |
| **Metabolic disease** | |  |  |  |  |  |  |  |  |  |
| Type 2 diabetes | 2 | 1 | 0.8 | 1 | 1 | 0 | 0 | 0 | 5.8 | Low |
| Prediabetes | 2 | 0 | 0 | 1 | 0 | 0 | 0 | 0 | 3 | Very low |
| Metabolic syndrome | 2 | 0 | 0 | 1 | 0 | 0 | 0 | 0 | 3 | Very low |
| Overweight/obesity | 2 | 1 | 0 | 1 | 0 | 0.5 | 0 | 0 | 4.5 | Low |
| **Aging-related disease** | |  |  |  |  |  |  |  |  |  |
| Total fracture | 2 | 1 | 0 | 1 | 0.5 | 0 | 0 | 0 | 4.5 | Low |
| Hip fracture | 2 | 1 | 0 | 1 | 0.5 | 0 | 0 | 1 | 5.5 | Low |
| Fall | 2 | 1 | 0 | 1 | 0 | 0 | 0 | 0 | 4 | Low |
| Frailty | 2 | 0 | 0 | 1 | 0 | 0 | 0 | 0 | 3 | Very low |
| Dementia | 2 | 1 | 0 | 1 | 0 | 0 | 0 | 0 | 4 | Low |
| Parkinson’s disease | 2 | 0 | 0 | 1 | 0 | 0.5 | 0 | 0 | 3.5 | Very low |

**Abbreviation：** CVD=cardiovascular disease; CHD=coronary heart disease.

**References**

1. O'Sullivan TA, Hafekost K, Mitrou F, Lawrence D. Food sources of saturated fat and the association with mortality: a meta-analysis. *Am J Public Health*. Sep 2013;103(9):e31-42. doi:10.2105/ajph.2013.301492

2. Guo J, Astrup A, Lovegrove JA, Gijsbers L, Givens DI, Soedamah-Muthu SS. Milk and dairy consumption and risk of cardiovascular diseases and all-cause mortality: dose–response meta-analysis of prospective cohort studies. Article. *European Journal of Epidemiology*. 2017;32(4):269-287. doi:10.1007/s10654-017-0243-1

3. Tong X, Chen GC, Zhang Z, Wei YL, Xu JY, Qin LQ. Cheese Consumption and Risk of All-Cause Mortality: A Meta-Analysis of Prospective Studies. *Nutrients*. Jan 13 2017;9(1)doi:10.3390/nu9010063

4. Lu W, Chen H, Niu Y, Wu H, Xia D, Wu Y. Dairy products intake and cancer mortality risk: a meta-analysis of 11 population-based cohort studies. *Nutr J*. Oct 21 2016;15(1):91. doi:10.1186/s12937-016-0210-9

5. Jin S, Je Y. Dairy Consumption and Total Cancer and Cancer-Specific Mortality: A Meta-Analysis of Prospective Cohort Studies. Article in Press. *Advances in nutrition (Bethesda, Md)*. 2021;doi:10.1093/advances/nmab135

6. Jin S, Kim Y, Je Y. Dairy Consumption and Risks of Colorectal Cancer Incidence and Mortality: A Meta-analysis of Prospective Cohort Studies. Article. *Cancer epidemiology, biomarkers & prevention : a publication of the American Association for Cancer Research, cosponsored by the American Society of Preventive Oncology*. 2020;29(11):2309-2322. doi:10.1158/1055-9965.EPI-20-0127

7. Sun Y, Lin LJ, Sang LX, Dai C, Jiang M, Zheng CQ. Dairy product consumption and gastric cancer risk: a meta-analysis. *World J Gastroenterol*. Nov 14 2014;20(42):15879-98. doi:10.3748/wjg.v20.i42.15879

8. Yang Y, Wang X, Yao Q, Qin L, Xu C. Dairy Product, Calcium Intake and Lung Cancer Risk: A Systematic Review with Meta-Analysis. *Sci Rep*. Feb 15 2016;6:20624. doi:10.1038/srep20624

9. Zhang K, Chen X, Zhang L, Deng Z. Fermented dairy foods intake and risk of cardiovascular diseases: A meta-analysis of cohort studies. Article. *Critical reviews in food science and nutrition*. 2020;60(7):1189-1194. doi:10.1080/10408398.2018.1564019

10. Alexander DD, Bylsma LC, Vargas AJ, et al. Dairy consumption and CVD: A systematic review and meta-analysis. Article. *British Journal of Nutrition*. 2016;115(4):737-750. doi:10.1017/S0007114515005000

11. Chen GC, Wang Y, Tong X, et al. Cheese consumption and risk of cardiovascular disease: a meta-analysis of prospective studies. Article. *European Journal of Nutrition*. 2017;56(8):2565-2575. doi:10.1007/s00394-016-1292-z

12. Chen Z, Ahmed M, Ha V, et al. Dairy Product Consumption and Cardiovascular Health: a Systematic Review and Meta-Analysis of Prospective Cohort Studies. Article in Press. *Advances in nutrition (Bethesda, Md)*. 2021;doi:10.1093/advances/nmab118

13. Gholami F, Khoramdad M, Shakiba E, Alimohamadi Y, Shafiei J, Firouzi A. Subgroup dairy products consumption on the risk of stroke and CHD: A systematic review and meta-analysis. *Med J Islam Repub Iran*. 2017;31:25. doi:10.18869/mjiri.31.25

14. Jakobsen MU, Trolle E, Outzen M, et al. Intake of dairy products and associations with major atherosclerotic cardiovascular diseases: a systematic review and meta-analysis of cohort studies. *Sci Rep*. Jan 14 2021;11(1):1303. doi:10.1038/s41598-020-79708-x

15. Qin LQ, Xu JY, Han SF, Zhang ZL, Zhao YY, Szeto IM. Dairy consumption and risk of cardiovascular disease: an updated meta-analysis of prospective cohort studies. Article. *Asia Pacific journal of clinical nutrition*. 2015;24(1):90-100. doi:10.6133/apjcn.2015.24.1.09

16. de Goede J, Soedamah-Muthu SS, Pan A, Gijsbers L, Geleijnse JM. Dairy Consumption and Risk of Stroke: A Systematic Review and Updated Dose-Response Meta-Analysis of Prospective Cohort Studies. *Journal of the American Heart Association*. May 20 2016;5(5)doi:10.1161/jaha.115.002787

17. Hu D, Huang J, Wang Y, Zhang D, Qu Y. Dairy foods and risk of stroke: A meta-analysis of prospective cohort studies. Article. *Nutrition, Metabolism and Cardiovascular Diseases*. 2014;24(5):460-469. doi:10.1016/j.numecd.2013.12.006

18. Heidari Z, Rashidi Pour Fard N, Clark CCT, Haghighatdoost F. Dairy products consumption and the risk of hypertension in adults: An updated systematic review and dose–response meta-analysis of prospective cohort studies. Article. *Nutrition, Metabolism and Cardiovascular Diseases*. 2021;31(7):1962-1975. doi:10.1016/j.numecd.2021.02.033

19. Soedamah-Muthu SS, Verberne LDM, Ding EL, Engberink MF, Geleijnse JM. Dairy consumption and incidence of hypertension: A dose-response meta-analysis of prospective cohort studies. Article. *Hypertension*. 2012;60(5):1131-1137. doi:10.1161/HYPERTENSIONAHA.112.195206

20. Ralston RA, Lee JH, Truby H, Palermo CE, Walker KZ. A systematic review and meta-analysis of elevated blood pressure and consumption of dairy foods. Review. *Journal of Human Hypertension*. 2012;26(1):3-13. doi:10.1038/jhh.2011.3

21. Zhang K, Dai H, Liang W, Zhang L, Deng Z. Fermented dairy foods intake and risk of cancer. Article. *International Journal of Cancer*. 2019;144(9):2099-2108. doi:10.1002/ijc.31959

22. Boyd NF, Martin LJ, Noffel M, Lockwood GA, Tritchler DL. A meta-analysis of studies of dietary fat and breast cancer risk. Article. *British Journal of Cancer*. 1993;68(3):627-636.

23. Kazemi A, Barati-Boldaji R, Soltani S, et al. Intake of Various Food Groups and Risk of Breast Cancer: A Systematic Review and Dose-Response Meta-Analysis of Prospective Studies. *Adv Nutr*. Jun 1 2021;12(3):809-849. doi:10.1093/advances/nmaa147

24. Wu Y, Huang R, Wang M, et al. Dairy foods, calcium, and risk of breast cancer overall and for subtypes defined by estrogen receptor status: a pooled analysis of 21 cohort studies. *Am J Clin Nutr*. Aug 2 2021;114(2):450-461. doi:10.1093/ajcn/nqab097

25. Li F, An SL, Zhou Y, et al. Milk and dairy consumption and risk of bladder cancer: A meta-analysis. Article. *Urology*. 2011;78(6):1298-1305. doi:10.1016/j.urology.2011.09.002

26. Acham M, Wesselius A, van Osch FHM, et al. Intake of milk and other dairy products and the risk of bladder cancer: a pooled analysis of 13 cohort studies. *Eur J Clin Nutr*. Jan 2020;74(1):28-35. doi:10.1038/s41430-019-0453-6

27. Aune D, Lau R, Chan DSM, et al. Dairy products and colorectal cancer risk: A systematic review and meta-analysis of cohort studies. Review. *Annals of Oncology*. 2012;23(1):37-45. doi:10.1093/annonc/mdr269

28. Barrubés L, Babio N, Becerra-Tomás N, Rosique-Esteban N, Salas-Salvadó J. Association Between Dairy Product Consumption and Colorectal Cancer Risk in Adults: A Systematic Review and Meta-Analysis of Epidemiologic Studies. *Adv Nutr*. May 1 2019;10(suppl_2):S190-s211. doi:10.1093/advances/nmy114

29. Vieira AR, Abar L, Chan DSM, et al. Foods and beverages and colorectal cancer risk: A systematic review and meta-analysis of cohort studies, an update of the evidence of the WCRF-AICR Continuous Update Project. Review. *Annals of Oncology*. 2017;28(8):1788-1802. doi:10.1093/annonc/mdx171

30. Ralston RA, Truby H, Palermo CE, Walker KZ. Colorectal cancer and nonfermented milk, solid cheese, and fermented milk consumption: a systematic review and meta-analysis of prospective studies. Review. *Critical reviews in food science and nutrition*. 2014;54(9):1167-1179. doi:10.1080/10408398.2011.629353

31. Cho E, Smith-Warner SA, Spiegelman D, et al. Dairy foods, calcium, and colorectal cancer: A pooled analysis of 10 cohort studies. Article. *Journal of the National Cancer Institute*. 2004;96(13):1015-1022. doi:10.1093/jnci/djh185

32. Liang Z, Song X, Hu J, et al. Fermented Dairy Food Intake and Risk of Colorectal Cancer: A Systematic Review and Meta-Analysis. *Front Oncol*. 2022;12:812679. doi:10.3389/fonc.2022.812679

33. Li X, Zhao J, Li P, Gao Y. Dairy products intake and endometrial cancer risk: A meta-analysis of observational studies. Article. *Nutrients*. 2018;10(1)doi:10.3390/nu10010025

34. Yang Y, Zhou J, Yang Y, Chen Z, Zheng X. Systematic review and meta-analysis: dairy consumption and hepatocellular carcinoma risk. Article. *Journal of Public Health (Germany)*. 2017;25(6):591-599. doi:10.1007/s10389-017-0806-3

35. Liao MQ, Gao XP, Yu XX, et al. Effects of dairy products, calcium and vitamin D on ovarian cancer risk: A meta-analysis of twenty-nine epidemiological studies. Article. *British Journal of Nutrition*. 2020;124(10):1001-1012. doi:10.1017/S0007114520001075

36. Khodavandi A, Alizadeh F, Razis AFA. Association between dietary intake and risk of ovarian cancer: a systematic review and meta-analysis. Review. *European Journal of Nutrition*. 2021;60(4):1707-1736. doi:10.1007/s00394-020-02332-y

37. Genkinger JM, Hunter DJ, Spiegelman D, et al. Dairy products and ovarian cancer: A pooled analysis of 12 cohort studies. Article. *Cancer Epidemiology Biomarkers and Prevention*. 2006;15(2):364-372. doi:10.1158/1055-9965.EPI-05-0484

38. Aune D, Navarro Rosenblatt DA, Chan DSM, et al. Dairy products, calcium, and prostate cancer risk: A systematic review and meta-analysis of cohort studies. Article. *American Journal of Clinical Nutrition*. 2015;101(1):87-117. doi:10.3945/ajcn.113.067157

39. Zhao Z, Wu D, Gao S, et al. The association between dairy products consumption and prostate cancer risk: a systematic review and meta-analysis. *Br J Nutr*. Aug 10 2022:1-18. doi:10.1017/S0007114522002380

40. Arafa A, Eshak ES, Dong JY, et al. Dairy intake and the risk of pancreatic cancer: the Japan Collaborative Cohort Study (JACC Study) and meta-analysis of prospective cohort studies. *Br J Nutr*. Oct 20 2021:1-9. doi:10.1017/s0007114521004232

41. Wang J, Li X, Zhang D. Dairy Product Consumption and Risk of Non-Hodgkin Lymphoma: A Meta-Analysis. *Nutrients*. Feb 27 2016;8(3):120. doi:10.3390/nu8030120

42. Guo LL, Li YT, Yao J, et al. Dairy Consumption and Risk of Conventional and Serrated Precursors of Colorectal Cancer: A Systematic Review and Meta-Analysis of Observational Studies. *J Oncol*. 2021;2021:9948814. doi:10.1155/2021/9948814

43. Aune D, Norat T, Romundstad P, Vatten LJ. Dairy products and the risk of type 2 diabetes: A systematic review and dose-response meta-analysis of cohort studies. Article. *American Journal of Clinical Nutrition*. 2013;98(4):1066-1083. doi:10.3945/ajcn.113.059030

44. Fan M, Li Y, Wang C, et al. Dietary protein consumption and the risk of type 2 diabetes: Adose-response meta-analysis of prospective studies. Review. *Nutrients*. 2019;11(11)doi:10.3390/nu11112783

45. Gao D, Ning N, Wang C, et al. Dairy products consumption and risk of type 2 diabetes: systematic review and dose-response meta-analysis. *PLoS One*. 2013;8(9):e73965. doi:10.1371/journal.pone.0073965

46. Gijsbers L, Ding EL, Malik VS, De Goede J, Geleijnse JM, Soedamah-Muthu SS. Consumption of dairy foods and diabetes incidence: A dose-response meta-analysis of observational studies. Article. *American Journal of Clinical Nutrition*. 2016;103(4):1111-1124. doi:10.3945/ajcn.115.123216

47. Khoramdad M, Rahimi M, Cheraghi Z, et al. The effect of dairy products subgroups consumption on the risk of diabetes: A systematic review and meta-analysis. Review. *Iranian Red Crescent Medical Journal*. 2017;19(3)doi:10.5812/ircmj.42064

48. Jin S, Je Y. Dairy Consumption and Risk of Metabolic Syndrome: Results from Korean Population and Meta-Analysis. *Nutrients*. May 8 2021;13(5)doi:10.3390/nu13051574

49. Babio N, Becerra-Tomás N, Nishi SK, et al. Total dairy consumption in relation to overweight and obesity in children and adolescents: A systematic review and meta-analysis. Article. *Obesity Reviews*. 2022;23(S1)doi:10.1111/obr.13400

50. Ong AM, Kang K, Weiler HA, Morin SN. Fermented Milk Products and Bone Health in Postmenopausal Women: A Systematic Review of Randomized Controlled Trials, Prospective Cohorts, and Case-Control Studies. *Adv Nutr*. Mar 1 2020;11(2):251-265. doi:10.1093/advances/nmz108

51. Matía-Martín P, Torrego-Ellacuría M, Larrad-Sainz A, Fernández-Pérez C, Cuesta-Triana F, Rubio-Herrera MÁ. Effects of Milk and Dairy Products on the Prevention of Osteoporosis and Osteoporotic Fractures in Europeans and Non-Hispanic Whites from North America: A Systematic Review and Updated Meta-Analysis. Article. *Advances in nutrition (Bethesda, Md)*. 2019;10(2):S120-S143. doi:10.1093/advances/nmy097

52. Bian S, Hu J, Zhang K, Wang Y, Yu M, Ma J. Dairy product consumption and risk of hip fracture: a systematic review and meta-analysis. *BMC Public Health*. Jan 22 2018;18(1):165. doi:10.1186/s12889-018-5041-5

53. Hidayat K, Du X, Shi BM, Qin LQ. Systematic review and meta-analysis of the association between dairy consumption and the risk of hip fracture: critical interpretation of the currently available evidence. Review. *Osteoporosis International*. 2020;31(8):1411-1425. doi:10.1007/s00198-020-05383-3

54. Jiang W, Ju C, Jiang H, Zhang D. Dairy foods intake and risk of Parkinson’s disease: a dose–response meta-analysis of prospective cohort studies. Article. *European Journal of Epidemiology*. 2014;29(9):613-619. doi:10.1007/s10654-014-9921-4

55. Drouin-Chartier JP, Li Y, Ardisson Korat AV, et al. Changes in dairy product consumption and risk of type 2 diabetes: Results from 3 large prospective cohorts of US men and women. *American Journal of Clinical Nutrition*. 2019;110(5):1201-1212. doi:10.1093/ajcn/nqz180

56. Chen M, Sun Q, Giovannucci E, Willett W, Mozaffarian D, Hu FB. Dairy consumption and risk of type 2 diabetes: 3 cohorts of us adults and an updated meta-analysis. *Circulation*. 2014;129((Chen M.; Sun Q.; Giovannucci E.; Willett W.; Mozaffarian D.; Hu F.B.) Harvard Sch of Public Health, Boston, MA, United States)

57. Yuzbashian E, Asghari G, Mirmiran P, Chan CB, Azizi F. Changes in dairy product consumption and subsequent type 2 diabetes among individuals with prediabetes: Tehran Lipid and Glucose Study. *Nutrition Journal*. 2021;20(1)doi:10.1186/s12937-021-00745-x

58. Cheraghi Z, Nedjat S, Mirmiran P, et al. Effects of food items and related nutrients on metabolic syndrome using Bayesian multilevel modelling using the Tehran Lipid and Glucose Study (TLGS): a cohort study. *BMJ Open*. Dec 18 2018;8(12):e020642. doi:10.1136/bmjopen-2017-020642

59. Yuzbashian E, Nosrati-Oskouie M, Asghari G, Chan CB, Mirmiran P, Azizi F. Associations of dairy intake with risk of incident metabolic syndrome in children and adolescents: Tehran Lipid and Glucose Study. *Acta Diabetologica*. 2021;58(4):447-457. doi:10.1007/s00592-020-01651-0

60. Fumeron F, Lamri A, Abi Khalil C, et al. Dairy consumption and the incidence of hyperglycemia and the metabolic syndrome: Results from a French prospective study, data from the epidemiological study on the insulin resistance syndrome (DESIR). *Diabetes Care*. 2011;34(4):813-817. doi:10.2337/dc10-1772

61. Fumeron F, Lamri A, Emery N, et al. Dairy products and the metabolic syndrome in a prospective study, DESIR. *Journal of the American College of Nutrition*. 2011;30(5):454-463.

62. Fairfield KM, Hunter DJ, Colditz GA, et al. A prospective study of dietary lactose and ovarian cancer. *Int J Cancer*. Jun 10 2004;110(2):271-7. doi:10.1002/ijc.20086

63. Kushi LH, Mink PJ, Folsom AR, et al. Prospective study of diet and ovarian cancer. *Am J Epidemiol*. Jan 1 1999;149(1):21-31. doi:10.1093/oxfordjournals.aje.a009723

64. Larsson SC, Bergkvist L, Wolk A. Milk and lactose intakes and ovarian cancer risk in the Swedish Mammography Cohort. *Am J Clin Nutr*. Nov 2004;80(5):1353-7. doi:10.1093/ajcn/80.5.1353

65. Bertone ER, Rosner BA, Hunter DJ, et al. Dietary fat intake and ovarian cancer in a cohort of US women. *Am J Epidemiol*. Jul 1 2002;156(1):22-31. doi:10.1093/aje/kwf008

66. Genkinger JM, Makambi KH, Palmer JR, Rosenberg L, Adams-Campbell LL. Consumption of dairy and meat in relation to breast cancer risk in the Black Women's Health Study. *Cancer Causes Control*. Apr 2013;24(4):675-84. doi:10.1007/s10552-013-0146-8

67. Kampman E, Goldbohm RA, Van Den Brandt PA, Van't Veer P. Fermented dairy products, calcium, and colorectal cancer in the Netherlands cohort study. *Cancer Research*. 1994;54(12):3186-3190.

68. Kearney J, Giovannucci E, Rimm EB, et al. Calcium, vitamin D, and dairy foods and the occurrence of colon cancer in men. *Am J Epidemiol*. May 1 1996;143(9):907-17. doi:10.1093/oxfordjournals.aje.a008834

69. Kesse E, Boutron-Ruault MC, Norat T, Riboli E, Clavel-Chapelon F, Grp EN. Dietary calcium, phosphorus, vitamin D, dairy products and the risk of colorectal adenoma and cancer among French women of the E3N-EPIC prospective study. *Int J Cancer*. Oct 20 2005;117(1):137-144. doi:10.1002/ijc.21148

70. Murphy N, Norat T, Ferrari P, et al. Consumption of Dairy Products and Colorectal Cancer in the European Prospective Investigation into Cancer and Nutrition (EPIC). *Plos One*. Sep 2 2013;8(9)doi:ARTN e72715

10.1371/journal.pone.0072715

71. Keszei AP, Schouten LJ, Goldbohm RA, Van Den Brandt PA. Dairy intake and the risk of bladder cancer in the netherlands cohort study on diet and cancer. *American Journal of Epidemiology*. 2010;171(4):436-446. doi:10.1093/aje/kwp399

72. Kiani F, Knutsen S, Singh P, Ursin G, Fraser G. Dietary risk factors for ovarian cancer: The adventist health study (United States). *Cancer Causes and Control*. 2006;17(2):137-146. doi:10.1007/s10552-005-5383-z

73. Koralek DO, Bertone-Johnson ER, Leitzmann MF, et al. Relationship between calcium, lactose, vitamin D, and dairy products and ovarian cancer. *Nutrition and Cancer-an International Journal*. 2006;56(1):22-30. doi:DOI 10.1207/s15327914nc5601_4

74. Larsson SC, Bergkvist L, Wolk A. High-fat dairy food and conjugated linoleic acid intakes in relation to colorectal cancer incidence in the Swedish Mammography Cohort. *American Journal of Clinical Nutrition*. 2005;82(4):894-900. doi:10.1093/ajcn/82.4.894

75. Michaud DS, Giovannucci E, Willett WC, Colditz GA, Fuchs CS. Dietary meat, dairy products, fat, and cholesterol and pancreatic cancer risk in a prospective study. *Am J Epidemiol*. Jun 15 2003;157(12):1115-25. doi:10.1093/aje/kwg098

76. Genkinger JM, Wang M, Li R, et al. Dairy products and pancreatic cancer risk: a pooled analysis of 14 cohort studies. *Ann Oncol*. Jun 2014;25(6):1106-15. doi:10.1093/annonc/mdu019

77. Mommers M, Schouten LJ, Goldbohm RA, Van Den Brandt PA. Dairy consumption and ovarian cancer risk in the Netherlands Cohort Study on diet and cancer. *British Journal of Cancer*. 2006;94(1):165-170. doi:10.1038/sj.bjc.6602890

78. Shin MH, Holmes MD, Hankinson SE, Wu K, Colditz GA, Willett WC. Intake of dairy products, calcium, and vitamin D and risk of breast cancer. *Jnci-J Natl Cancer I*. Sep 4 2002;94(17):1301-1311.

79. Singh PN, Fraser GE. Dietary risk factors for colon cancer in a low-risk population. *Am J Epidemiol*. Oct 15 1998;148(8):761-74. doi:10.1093/oxfordjournals.aje.a009697

80. Voorrips LE, Brants HA, Kardinaal AF, Hiddink GJ, van den Brandt PA, Goldbohm RA. Intake of conjugated linoleic acid, fat, and other fatty acids in relation to postmenopausal breast cancer: the Netherlands Cohort Study on Diet and Cancer. *Am J Clin Nutr*. Oct 2002;76(4):873-82. doi:10.1093/ajcn/76.4.873

81. Papadimitriou N, Bouras E, van den Brandt PA, et al. A Prospective Diet-Wide Association Study for Risk of Colorectal Cancer in EPIC. *Clinical Gastroenterology and Hepatology*. 2022;20(4):864-873.e13. doi:10.1016/j.cgh.2021.04.028

82. Bongard V, Arveiler D, Dallongeville J, et al. Food groups associated with a reduced risk of 15-year all-cause death. 2016;70(6):715‐722. doi:10.1038/ejcn.2016.19

83. Tognon G, Nilsson LM, Shungin D, et al. Nonfermented milk and other dairy products: associations with all-cause mortality. *Am J Clin Nutr*. Jun 2017;105(6):1502-1511. doi:10.3945/ajcn.116.140798

84. Fraser GE, Sumbureru D, Pribis P, Neil RL, Frankson MA. Association among health habits, risk factors, and all-cause mortality in a black California population. *Epidemiology*. Mar 1997;8(2):168-74. doi:10.1097/00001648-199703000-00008

85. Fraser GE, Shavlik DJ. Risk factors for all-cause and coronary heart disease mortality in the oldest-old. The Adventist Health Study. *Arch Intern Med*. Oct 27 1997;157(19):2249-58.

86. Zupo R, Sardone R, Donghia R, et al. Traditional dietary patterns and risk of mortality in a longitudinal cohort of the salus in apulia study. *Nutrients*. 2020;12(4)doi:10.3390/nu12041070

87. Downer MK, Batista JL, Mucci LA, et al. Dairy intake in relation to prostate cancer survival. *International Journal of Cancer*. 2017;140(9):2060-2069. doi:10.1002/ijc.30642

88. Dik VK, Murphy N, Siersema PD, et al. Prediagnostic intake of dairy products and dietary calcium and colorectal cancer survival-results from the EPIC cohort study. *Cancer Epidemiology Biomarkers and Prevention*. 2014;23(9):1813-1823. doi:10.1158/1055-9965.EPI-14-0172

89. Andersen JLM, Hansen L, Thomsen BLR, Christiansen LR, Dragsted LO, Olsen A. Pre- and post-diagnostic intake of whole grain and dairy products and breast cancer prognosis: the Danish Diet, Cancer and Health cohort. *Breast Cancer Research and Treatment*. 2020;179(3):743-753. doi:10.1007/s10549-019-05497-1

90. Miyake Y, Sasaki S, Tanaka K, Hirota Y. Dairy food, calcium and vitamin D intake in pregnancy, and wheeze and eczema in infants. *European Respiratory Journal*. 2010;35(6):1228-1234. doi:10.1183/09031936.00100609

91. Yalçin SS, Örün E, Mutlu B, et al. Why are they having infant colic? A nested case-control study. *Paediatric and Perinatal Epidemiology*. 2010;24(6):584-596. doi:10.1111/j.1365-3016.2010.01150.x

92. Ito M, Takamori A, Yoneda S, et al. Fermented foods and preterm birth risk from a prospective large cohort study: The Japan Environment and Children's study. *Environmental Health and Preventive Medicine*. 2019;24(1)doi:10.1186/s12199-019-0782-z

93. Nicklaus S, Divaret-Chauveau A, Chardon ML, et al. The protective effect of cheese consumption at 18 months on allergic diseases in the first 6 years. *Allergy: European Journal of Allergy and Clinical Immunology*. 2019;74(4):788-798. doi:10.1111/all.13650

94. Opstelten JL, Leenders M, Dik VK, et al. Dairy products, dietary calcium, and risk of inflammatory bowel disease: Results from a European prospective cohort investigation. *Inflammatory Bowel Diseases*. 2016;22(6):1403-1411. doi:10.1097/MIB.0000000000000798

95. Miyake Y, Tanaka K, Okubo H, Sasaki S, Furukawa S, Arakawa M. Milk intake during pregnancy is inversely associated with the risk of postpartum depressive symptoms in Japan: the Kyushu Okinawa Maternal and Child Health Study. *Nutrition Research*. 2016;36(9):907-913. doi:10.1016/j.nutres.2016.06.001

96. Berkey CS, Willett WC, Tamimi RM, Rosner B, Frazier AL, Colditz GA. Dairy intakes in older girls and risk of benign breast disease in young women. *Cancer Epidemiology Biomarkers and Prevention*. 2013;22(4):670-674. doi:10.1158/1055-9965.EPI-12-1133

97. Orta OR, Terry KL, Missmer SA, Harris HR. Dairy and related nutrient intake and risk of uterine leiomyoma: A prospective cohort study. *Human Reproduction*. 2020;35(2):453-463. doi:10.1093/humrep/dez278

98. Yuan S, Bruzelius M, Damrauer SM, et al. Anti-inflammatory diet and venous thromboembolism: Two prospective cohort studies. *Nutrition, Metabolism and Cardiovascular Diseases*. 2021;31(10):2831-2838. doi:10.1016/j.numecd.2021.06.021

99. Niinistö S, Takkinen HM, Uusitalo L, et al. Maternal dietary fatty acid intake during pregnancy and the risk of preclinical and clinical type 1 diabetes in the offspring. *British Journal of Nutrition*. 2014;111(5):895-903. doi:10.1017/S0007114513003073

100. Tanaka K, Miyake Y, Sasaki S, Hirota Y. Dairy products and calcium intake during pregnancy and dental caries in children. *Nutrition Journal*. 2012;11(1)doi:10.1186/1475-2891-11-33

101. Matthews VL, Knutsen SF, Beeson WL, Fraser GE. Soy milk and dairy consumption is independently associated with ultrasound attenuation of the heel bone among postmenopausal women: the Adventist Health Study-2. *Nutr Res*. 2011;31(10):766-75. doi:10.1016/j.nutres.2011.09.016

102. Camacho-Barcia L, Bullo M, Garcia-Gavilan JF, et al. Dairy products intake and the risk of incident cataracts surgery in an elderly Mediterranean population: results from the PREDIMED study. 2019;58(2):619‐627. doi:10.1007/s00394-018-1647-8

103. Littlejohns TJ, Neal NL, Bradbury KE, Heers H, Allen NE, Turney BW. Fluid Intake and Dietary Factors and the Risk of Incident Kidney Stones in UK Biobank: A Population-based Prospective Cohort Study. *European Urology Focus*. 2020;6(4):752-761. doi:10.1016/j.euf.2019.05.002
