# Supplementary material for: Cheese consumption and multiple health outcomes: an umbrella review and updated meta-analysis of prospective studies
Source: Adv Nutr. 2023 Jun 15;14(5):1170–86. doi: 10.1016/j.advnut.2023.06.007 (PMC10509445; doi:10.1016/j.advnut.2023.06.007)
Supplement: Multimedia component10 [file mmc10.docx]

Cheese consumption and multiple health outcomes: an umbrella review and updated meta-analysis of prospective studies

Mingjie Zhang, Xiaocong Dong, Zihui Huang, Xue Li, Yue Zhao, Yingyao Wang, Huilian Zhu, Aiping Fang, Edward L. Giovannucci

**List of Supplementary Figures**

[Supplementary Figure 28. Association between cheese consumption (highest vs. lowest intake level) and the risk of (A) total breast cancer, (B) ER+ breast cancer, and (C) ER- breast cancer . 2](#_Toc134633419)

[Supplementary Figure 29. Association between cheese consumption (per 30 g/d increment) and breast cancer risk. 3](#_Toc134633420)

[Supplementary Figure 30. Association between cheese consumption (highest vs. lowest intake level) and the risk of (A) bladder cancer, (B) pancreatic cancer, (C) endometrial cancer, (D) hepatocellular carcinoma, and (E) ovarian cancer. 5](#_Toc134633421)


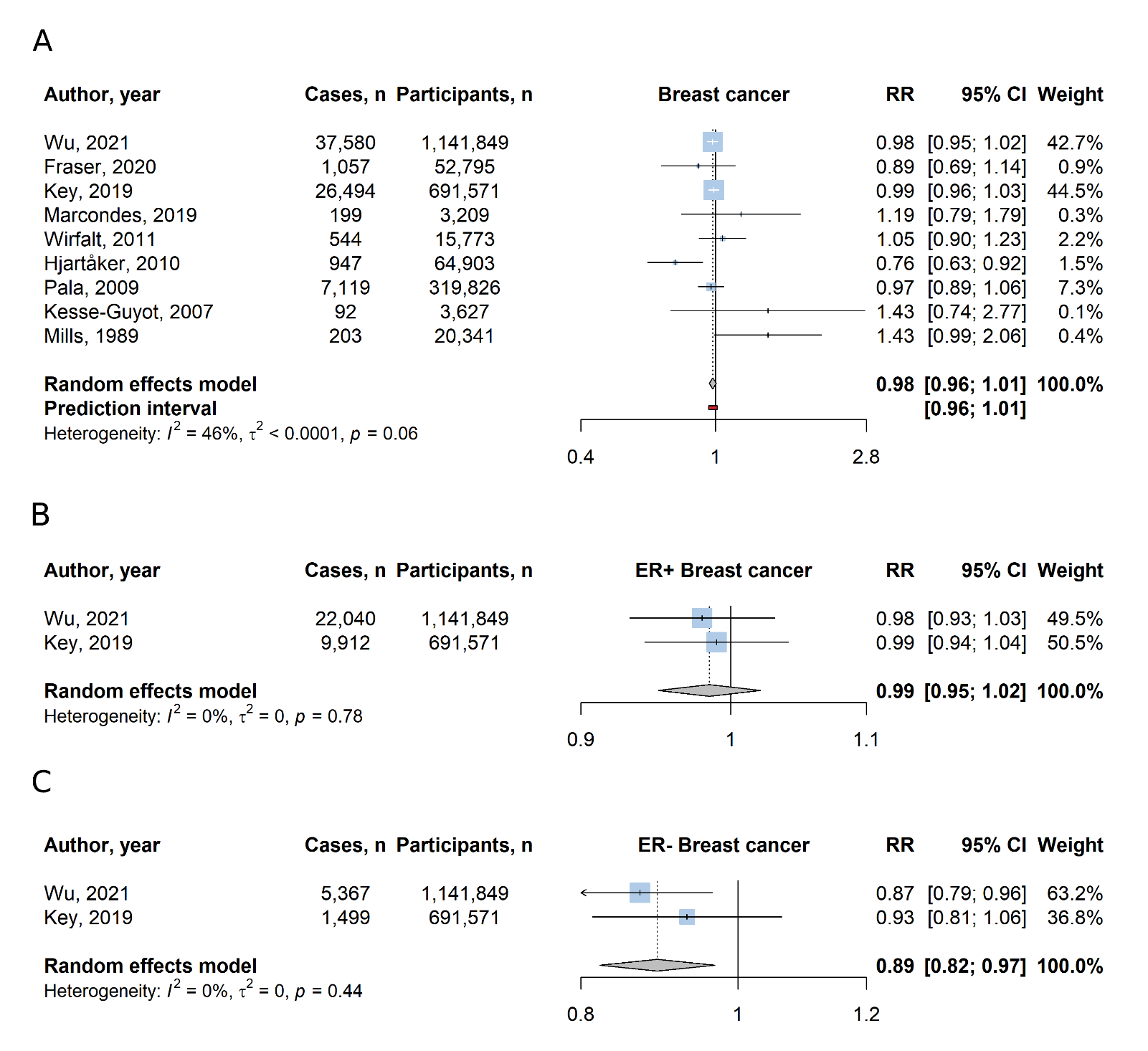


**Supplementary Figure 28. Association between cheese consumption (highest vs. lowest intake level) and the risk of (A) total breast cancer, (B) ER+** **breast cancer, and (C) ER- breast cancer .**

Study-specific effect sizes are visualized in squares and the size of squares is proportional to the specific study weight to the overall meta-analysis. Horizontal lines represent 95% CIs. Diamonds demonstrate the pooled relative risk and 95% CIs. ER+ breast cancer = Estrogen-receptor positive breast cancer; ER- breast cancer = Estrogen-receptor negative breast cancer.


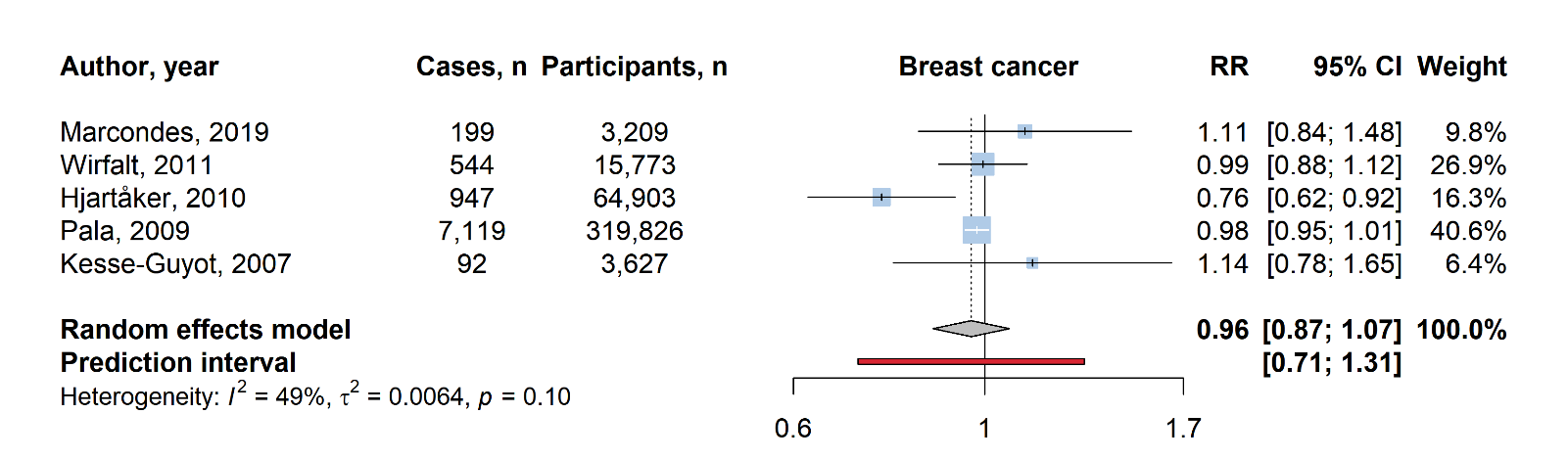


**Supplementary Figure 29. Association between cheese consumption (per 30 g/d increment) and breast cancer risk.**

Study-specific effect sizes are visualized in squares and the size of squares is proportional to the specific study weight to the overall meta-analysis. Horizontal lines represent 95% CIs. Diamonds demonstrate the pooled relative risk and 95% CIs.


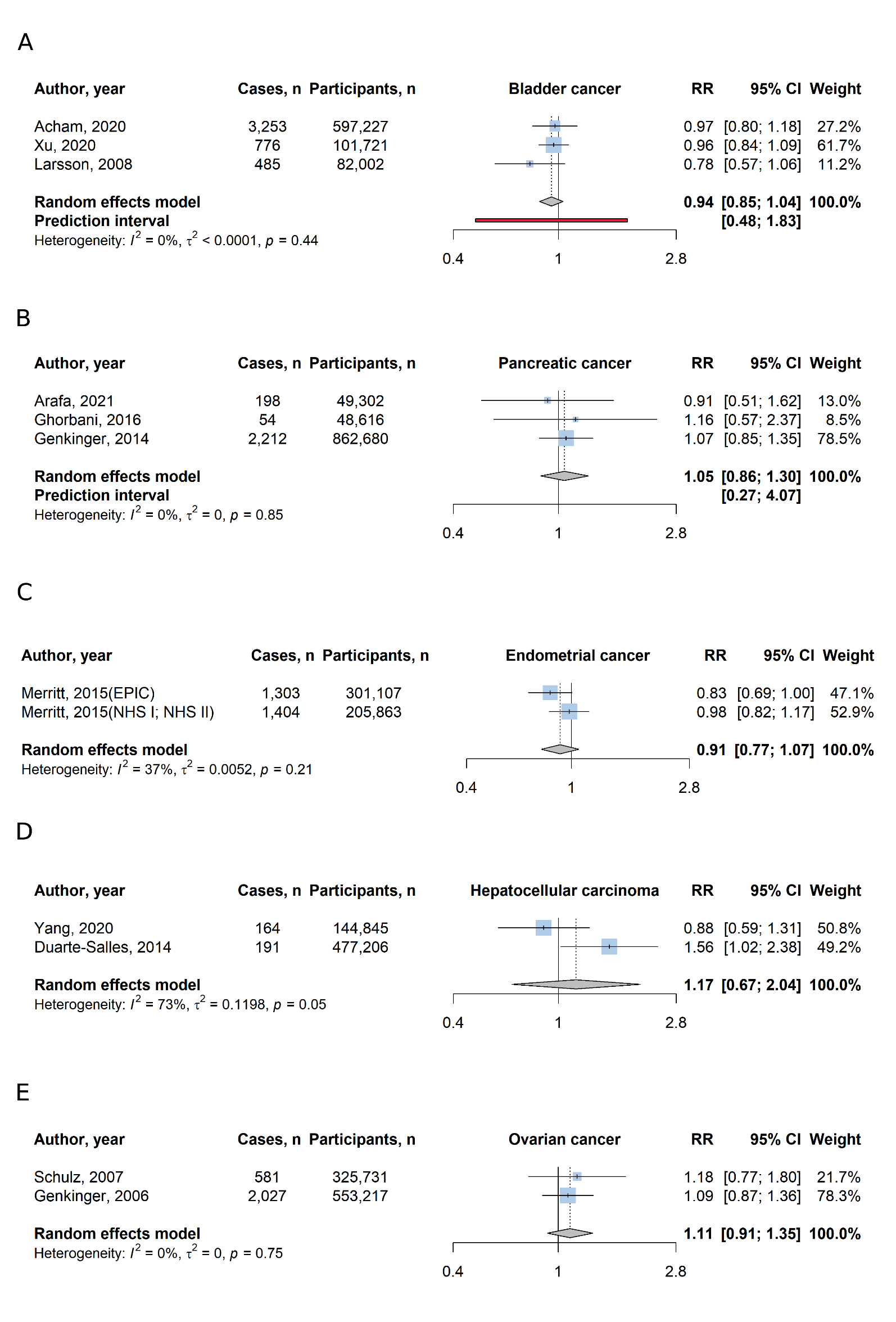


**Supplementary Figure 30. Association between cheese consumption (highest vs. lowest intake level) and the risk of (A) bladder cancer, (B) pancreatic cancer, (C) endometrial cancer, (D) hepatocellular carcinoma, and (E) ovarian cancer.**

Study-specific effect sizes are visualized in squares and the size of squares is proportional to the specific study weight to the overall meta-analysis. Horizontal lines represent 95% CIs. Diamonds demonstrate the pooled relative risk and 95% CIs. EPIC=European Prospective Investigation into Cancer and Nutrition; NHS I=Nurses’ Health Study (baseline: 1980); NHS Ⅱ= Nurses’ Health Study Ⅱ (baseline: 1991).
