# Supplementary material for: Cheese consumption and multiple health outcomes: an umbrella review and updated meta-analysis of prospective studies
Source: Adv Nutr. 2023 Jun 15;14(5):1170–86. doi: 10.1016/j.advnut.2023.06.007 (PMC10509445; doi:10.1016/j.advnut.2023.06.007)
Supplement: Multimedia component11 [file mmc11.docx]

Cheese consumption and multiple health outcomes: an umbrella review and updated meta-analysis of prospective studies

Mingjie Zhang, Xiaocong Dong, Zihui Huang, Xue Li, Yue Zhao, Yingyao Wang, Huilian Zhu, Aiping Fang, Edward L. Giovannucci

**List of Supplementary Figures**

[Supplementary Figure 31. Association between cheese consumption (highest vs. lowest intake level) and type 2 diabetes risk. 2](#_Toc128061819)

[Supplementary Figure 32. Association between cheese consumption (per 30 g/d increment) and type 2 diabetes risk. 3](#_Toc128061820)

[Supplementary Figure 33. Association between cheese consumption (highest vs. lowest level of intake) and prediabetes risk. 4](#_Toc128061821)

[Supplementary Figure 34. Association between cheese consumption (per 30g/d increment) and prediabetes risk. 4](#_Toc128061822)

[Supplementary Figure 35. Association between cheese consumption (highest vs. lowest intake level) and the risk of (A) metabolic syndrome and (B) overweight/obesity. 5](#_Toc128061823)


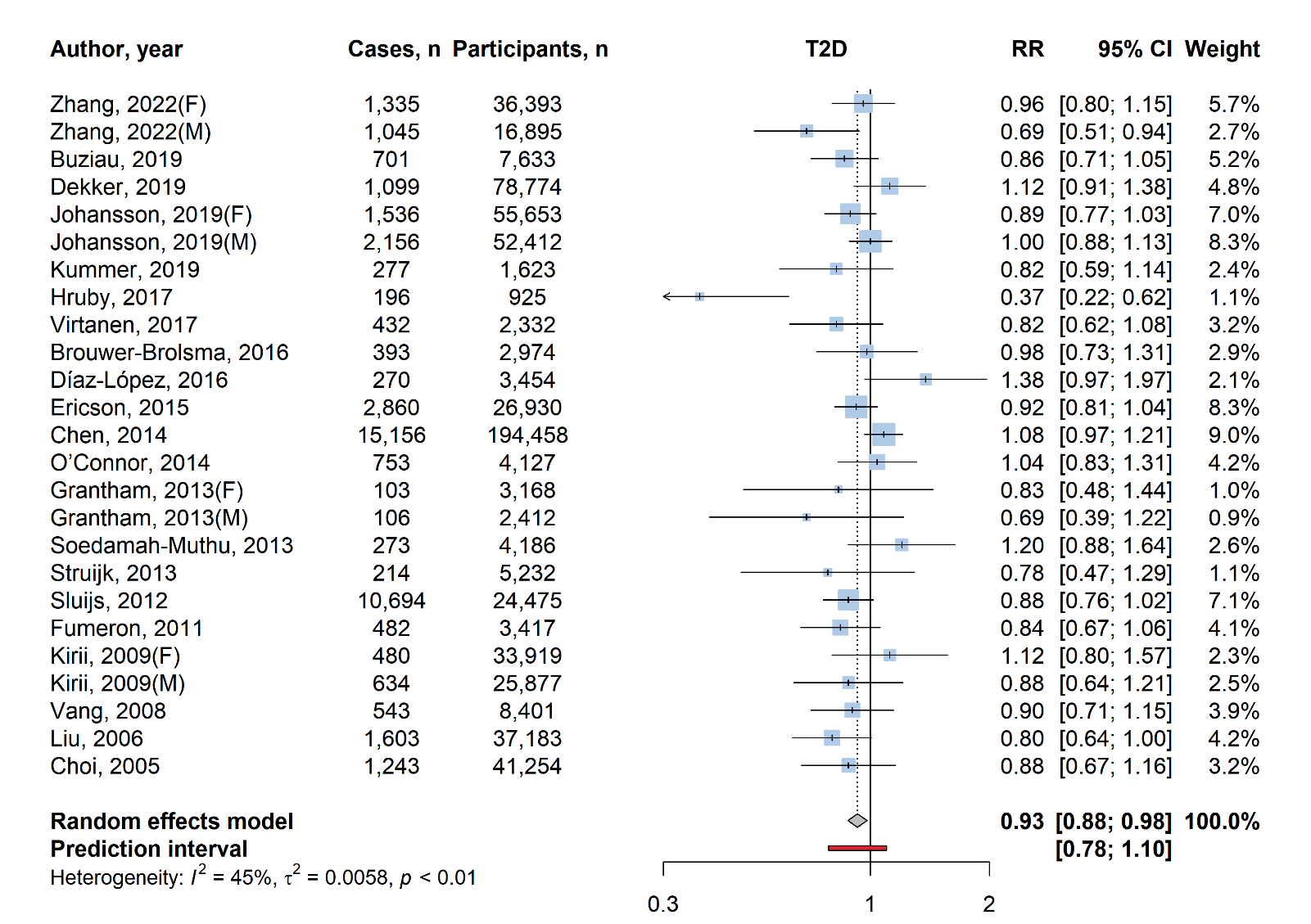


## Supplementary Figure 31. Association between cheese consumption (highest vs. lowest intake level) and type 2 diabetes risk.

Study-specific effect sizes are visualized in squares and the size of squares is proportional to the specific study weight to the overall meta-analysis. Horizontal lines represent 95% CIs. Diamonds demonstrate the pooled relative risk and 95% CIs. T2D= type 2 diabetes; F=female; M=male.


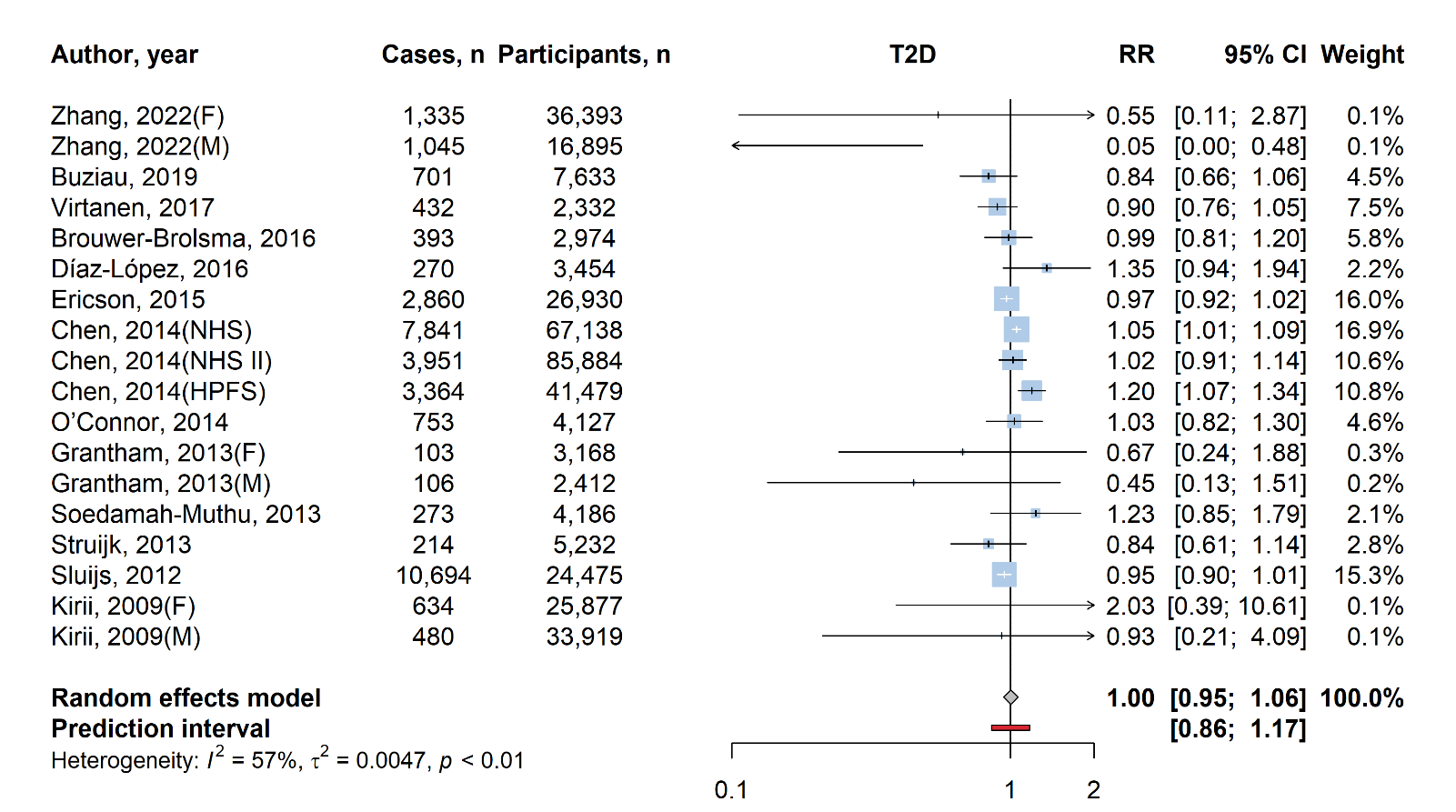


**Supplementary Figure 32. Association between cheese consumption (per 30 g/d increment) and type 2 diabetes risk.**

Study-specific effect sizes are visualized in squares and the size of squares is proportional to the specific study weight to the overall meta-analysis. Horizontal lines represent 95% CIs. Diamonds demonstrate the pooled relative risk and 95% CIs. T2D= type 2 diabetes; F=female; M=male; NHS= the Nurses' Health Study cohort; NHS Ⅱ= Nurses’ Health Study II; HPFS= Health Professional Follow-up Study.


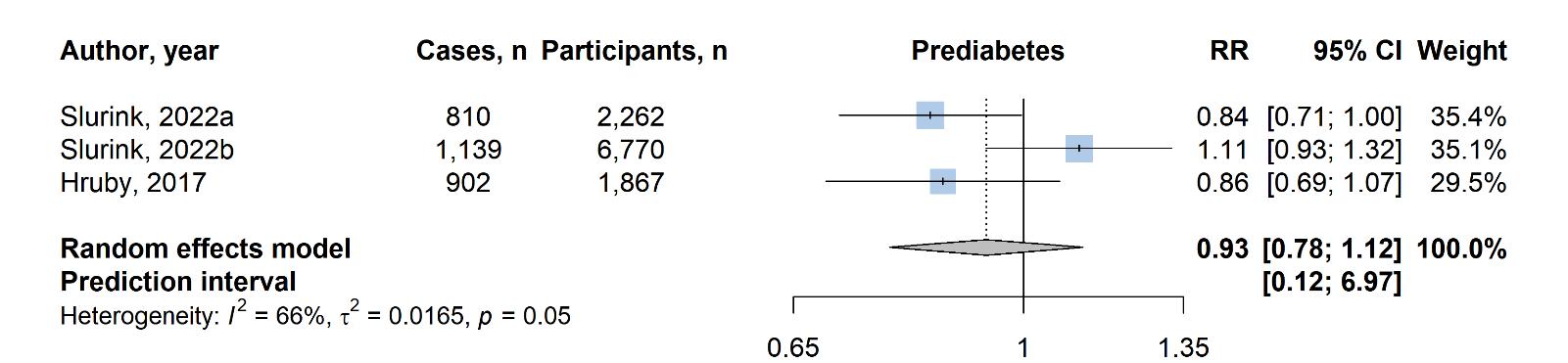


## Supplementary Figure 33. Association between cheese consumption (highest vs. lowest level of intake) and prediabetes risk.

Study-specific effect sizes are visualized in squares and the size of squares is proportional to the specific study weight to the overall meta-analysis. Horizontal lines represent 95% CIs. Diamonds demonstrate the pooled relative risk and 95% CIs. Slurink, 2022a is the result from article “Dairy product consumption and incident prediabetes in Dutch middle‑aged adults: the Hoorn Studies prospective cohort”; Slurink, 2022b is the result from article “Dairy Product Consumption in Relation to Incident Prediabetes and Longitudinal Insulin Resistance in the Rotterdam Study”.


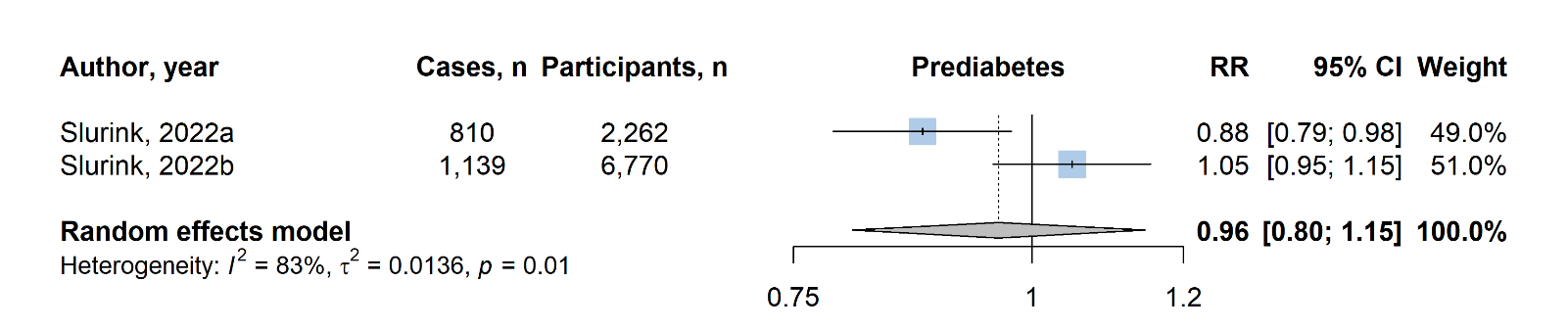


**Supplementary Figure 34. Association between cheese consumption (per 30g/d increment) and prediabetes risk.**

Study-specific effect sizes are visualized in squares and the size of squares is proportional to the specific study weight to the overall meta-analysis. Horizontal lines represent 95% CIs. Diamonds demonstrate the pooled relative risk and 95% CIs. Slurink, 2022a is the result from article “Dairy product consumption and incident prediabetes in Dutch middle‑aged adults: the Hoorn Studies prospective cohort”; Slurink, 2022b is the result from article “Dairy Product Consumption in Relation to Incident Prediabetes and Longitudinal Insulin Resistance in the Rotterdam Study”.


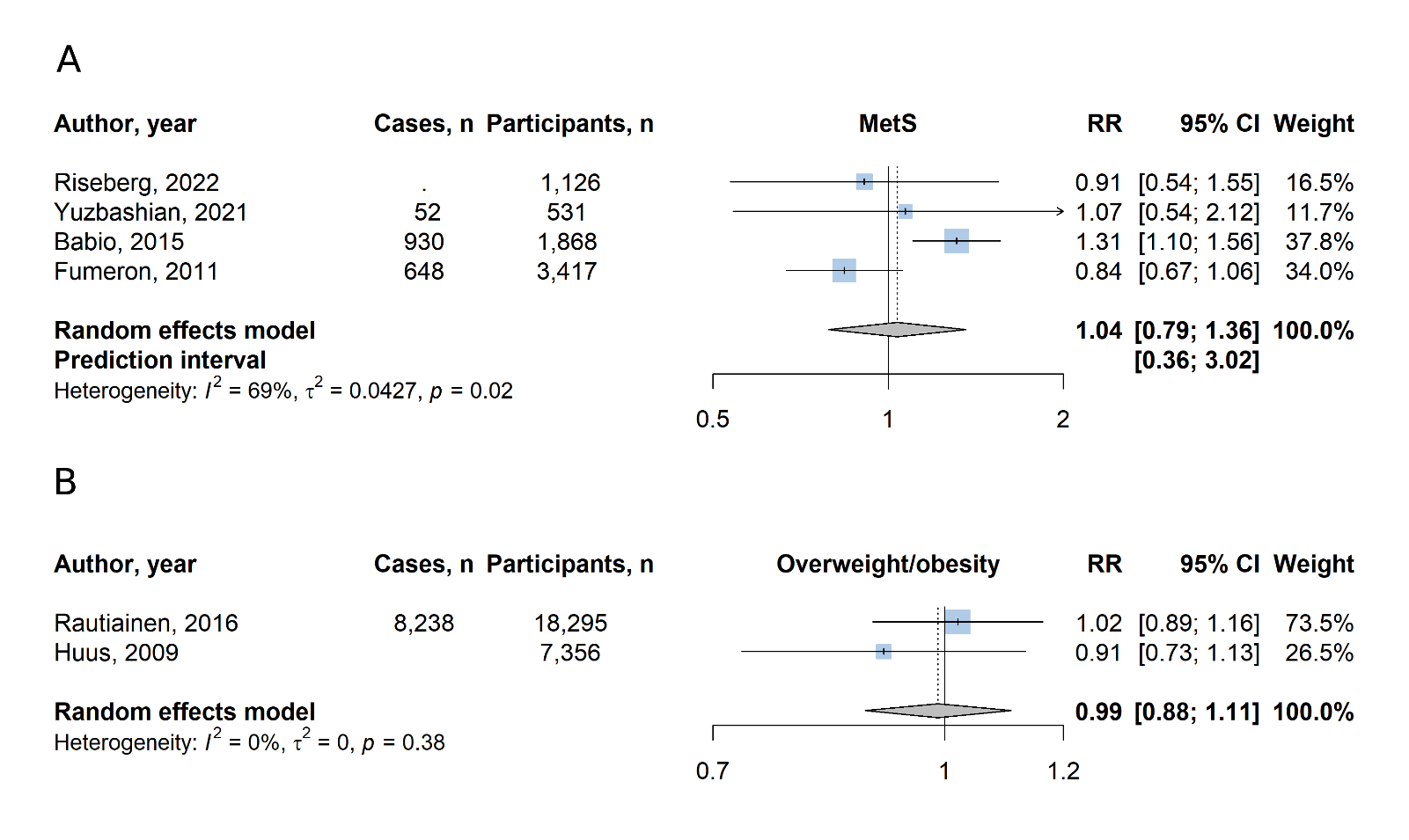


## Supplementary Figure 35. Association between cheese consumption (highest vs. lowest intake level) and the risk of (A) metabolic syndrome and (B) overweight/obesity.

Study-specific effect sizes are visualized in squares and the size of squares is proportional to the specific study weight to the overall meta-analysis. Horizontal lines represent 95% CIs. Diamonds demonstrate the pooled relative risk and 95% CIs. MetS= Metabolic syndrome.
