# Supplementary material for: Cheese consumption and multiple health outcomes: an umbrella review and updated meta-analysis of prospective studies
Source: Adv Nutr. 2023 Jun 15;14(5):1170–86. doi: 10.1016/j.advnut.2023.06.007 (PMC10509445; doi:10.1016/j.advnut.2023.06.007)
Supplement: Multimedia component12 [file mmc12.docx]

Cheese consumption and multiple health outcomes: an umbrella review and updated meta-analysis of prospective studies

Mingjie Zhang, Xiaocong Dong, Zihui Huang, Xue Li, Yue Zhao, Yingyao Wang, Huilian Zhu, Aiping Fang, Edward L. Giovannucci

**List of Supplementary Figures**

[Supplementary Figure 36. Association between cheese consumption (highest vs. lowest intake level) and total fracture risk. 2](#_Toc128061926)

[Supplementary Figure 37. Association between cheese consumption (per 30 g/d increment) and total fracture risk. 2](#_Toc128061927)

[Supplementary Figure 38. Association between cheese consumption (highest vs. lowest intake level) and hip fracture risk. 3](#_Toc128061928)

[Supplementary Figure 39. Association between cheese consumption (per 30 g/d increment) and hip fracture risk. 3](#_Toc128061929)

[Supplementary Figure 40. Association between cheese consumption (highest vs. lowest intake level) and the risk of (A) fall, (B) frailty, and (C) dementia. 4](#_Toc128061930)

[Supplementary Figure 41. Association between cheese consumption (per 30 g/d increment) and fall risk. 5](#_Toc128061931)


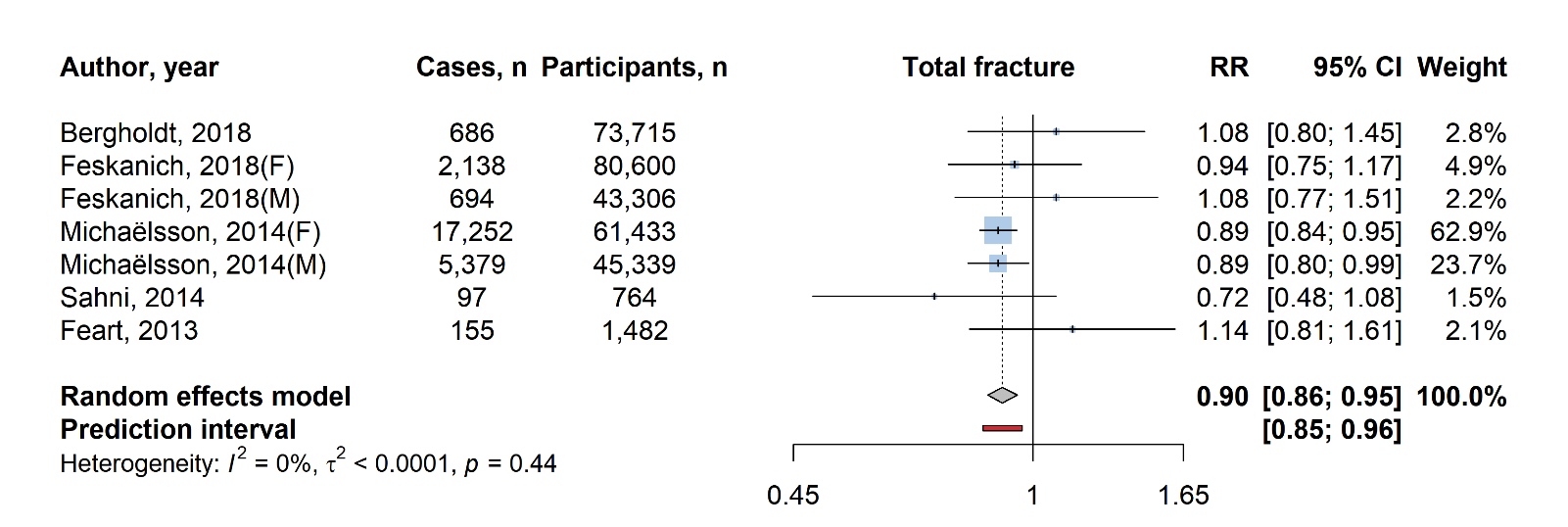


## Supplementary Figure 36. Association between cheese consumption (highest vs. lowest intake level) and total fracture risk.

Study-specific effect sizes are visualized in squares and the size of squares is proportional to the specific study weight to the overall meta-analysis. Horizontal lines represent 95% CIs. Diamonds demonstrate the pooled relative risk and 95% CIs. F=female; M=male.


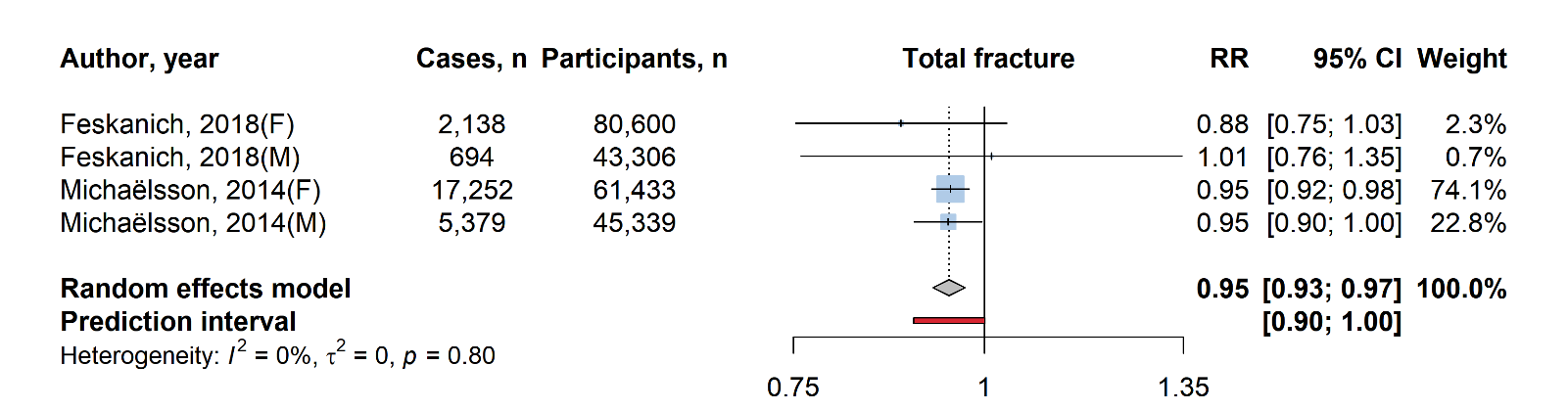


## Supplementary Figure 37. Association between cheese consumption (per 30 g/d increment) and total fracture risk.

Study-specific effect sizes are visualized in squares and the size of squares is proportional to the specific study weight to the overall meta-analysis. Horizontal lines represent 95% CIs. Diamonds demonstrate the pooled relative risk and 95% CIs. F=female; M=male.


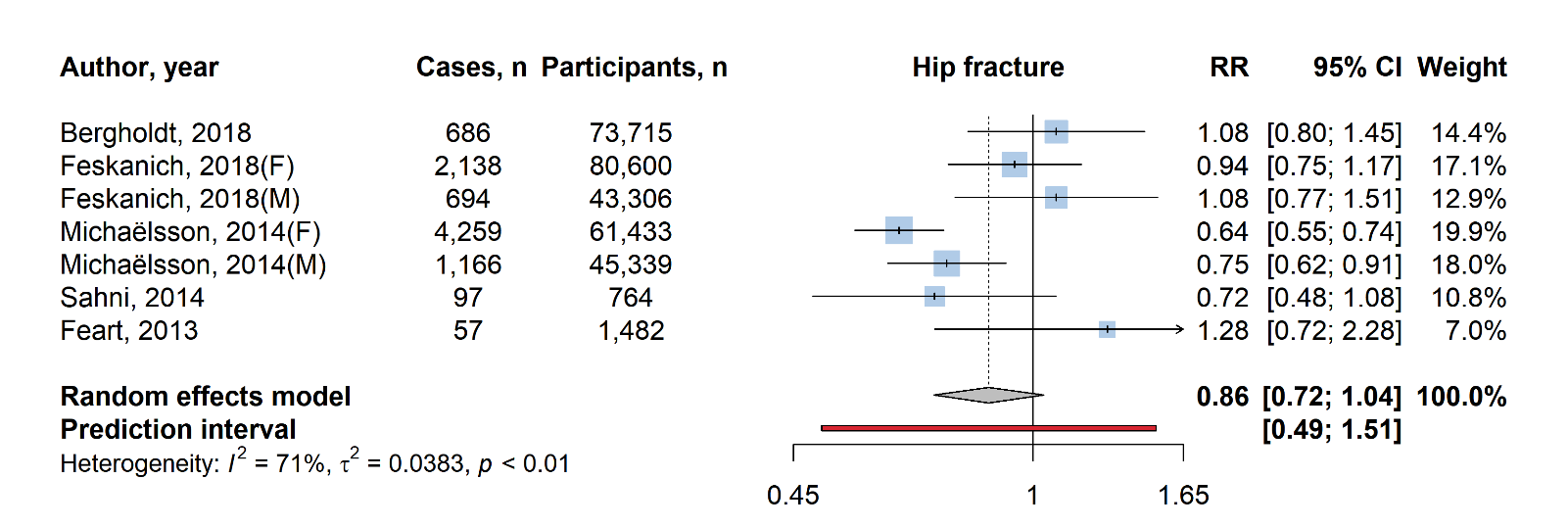


## Supplementary Figure 38. Association between cheese consumption (highest vs. lowest intake level) and hip fracture risk.

Study-specific effect sizes are visualized in squares and the size of squares is proportional to the specific study weight to the overall meta-analysis. Horizontal lines represent 95% CIs. Diamonds demonstrate the pooled relative risk and 95% CIs. F=female; M=male.


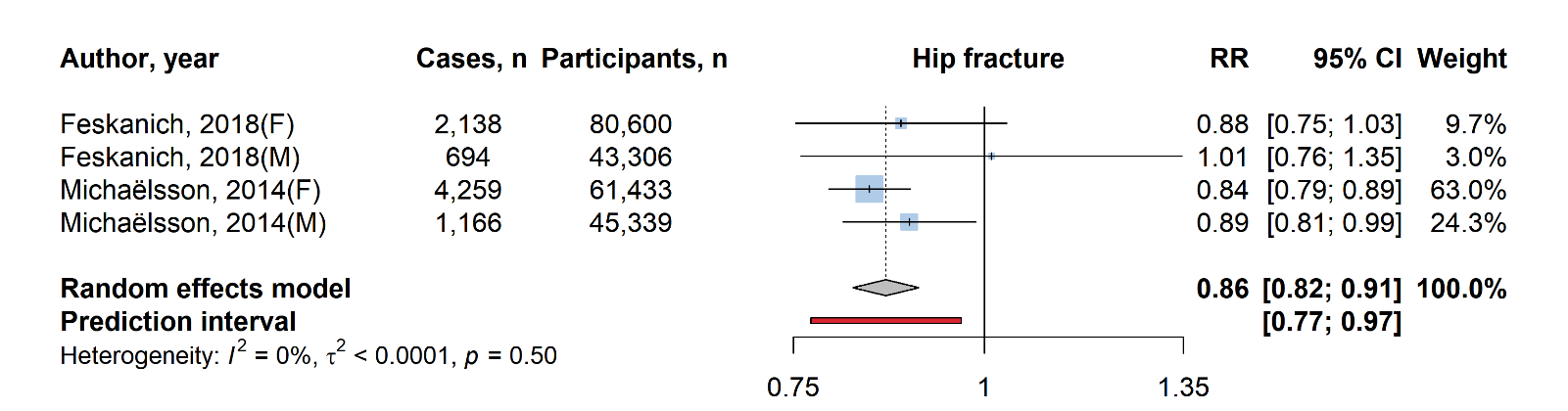


**Supplementary Figure 39. Association between cheese consumption (per 30 g/d increment) and hip fracture risk.**

Study-specific effect sizes are visualized in squares and the size of squares is proportional to the specific study weight to the overall meta-analysis. Horizontal lines represent 95% CIs. Diamonds demonstrate the pooled relative risk and 95% CIs. F=female; M=male.


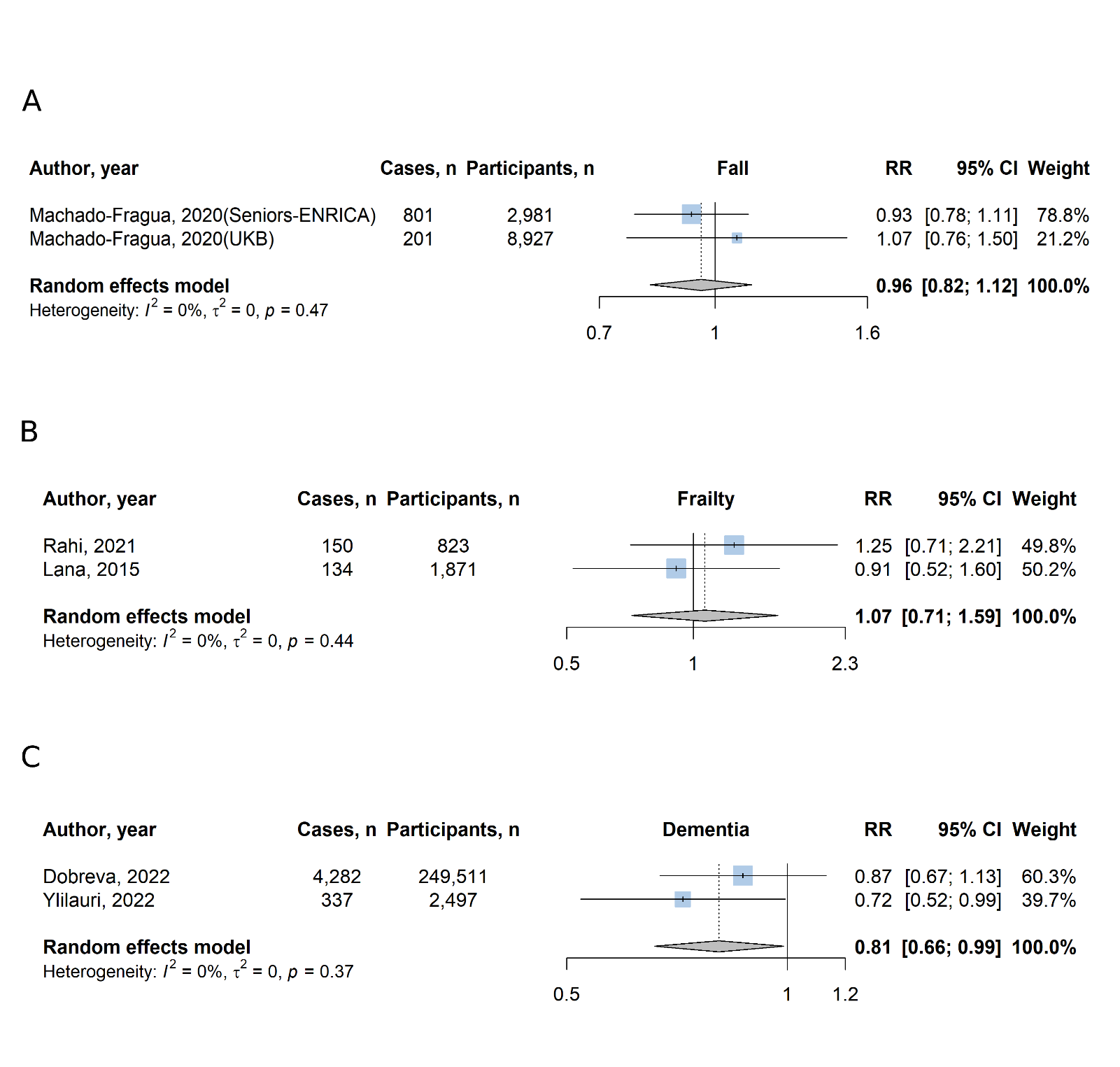


**Supplementary Figure 40. Association between cheese consumption (highest vs. lowest intake level) and the risk of (A) fall, (B) frailty, and (C) dementia.**

Study-specific effect sizes are visualized in squares and the size of squares is proportional to the specific study weight to the overall meta-analysis. Horizontal lines represent 95% CIs. Diamonds demonstrate the pooled relative risk and 95% CIs. ENRICA: Study on Nutrition and Cardiovascular Risk in Spain; Of them, participants aged ≥60y comprised the Seniors-ENRICA cohort; UKB= UK Biobank.


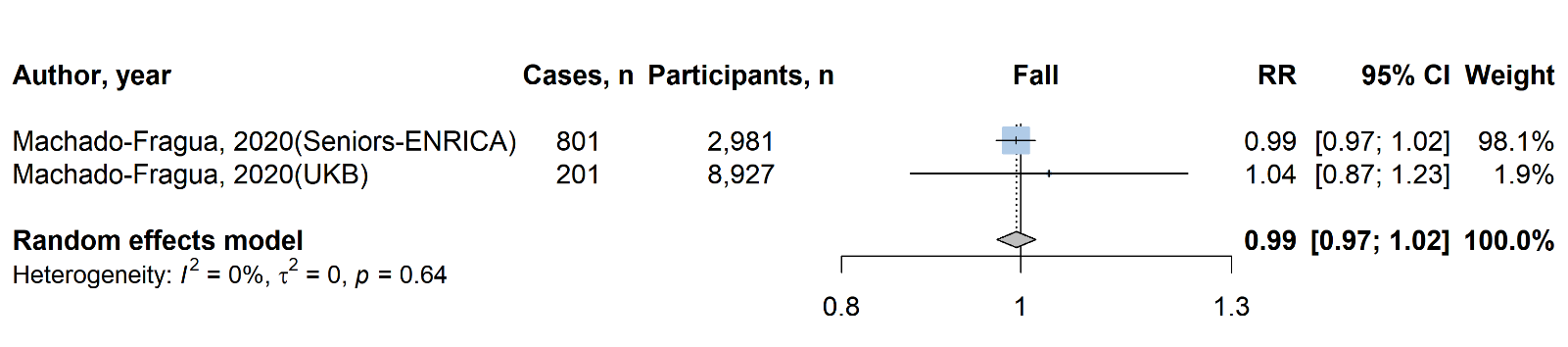


**Supplementary Figure 41. Association between cheese consumption (per 30 g/d increment) and fall risk.**

Study-specific effect sizes are visualized in squares and the size of squares is proportional to the specific study weight to the overall meta-analysis. Horizontal lines represent 95% CIs. Diamonds demonstrate the pooled relative risk and 95% CIs. ENRICA: Study on Nutrition and Cardiovascular Risk in Spain; Of them, participants aged ≥60y comprised the Seniors-ENRICA cohort; UKB= UK Biobank.
