# Supplementary material for: Cheese consumption and multiple health outcomes: an umbrella review and updated meta-analysis of prospective studies
Source: Adv Nutr. 2023 Jun 15;14(5):1170–86. doi: 10.1016/j.advnut.2023.06.007 (PMC10509445; doi:10.1016/j.advnut.2023.06.007)
Supplement: Multimedia component13 [file mmc13.docx]

Cheese consumption and multiple health outcomes: an umbrella review and updated meta-analysis of prospective studies

Mingjie Zhang, Xiaocong Dong, Zihui Huang, Xue Li, Yue Zhao, Yingyao Wang, Huilian Zhu, Aiping Fang, Edward L. Giovannucci

**List of Supplementary Figures**

[Supplementary Figure 42. Dose-response association between cheese consumption and the risk of (A) overall cancer, (B) prostate cancer, (C) colorectal cancer and (D) breast cancer. 2](#_Toc128062039)

[Supplementary Figure 43. Dose-response association between cheese consumption and the risk of (A) type 2 diabetes and (B) hip fracture. 3](#_Toc128062040)


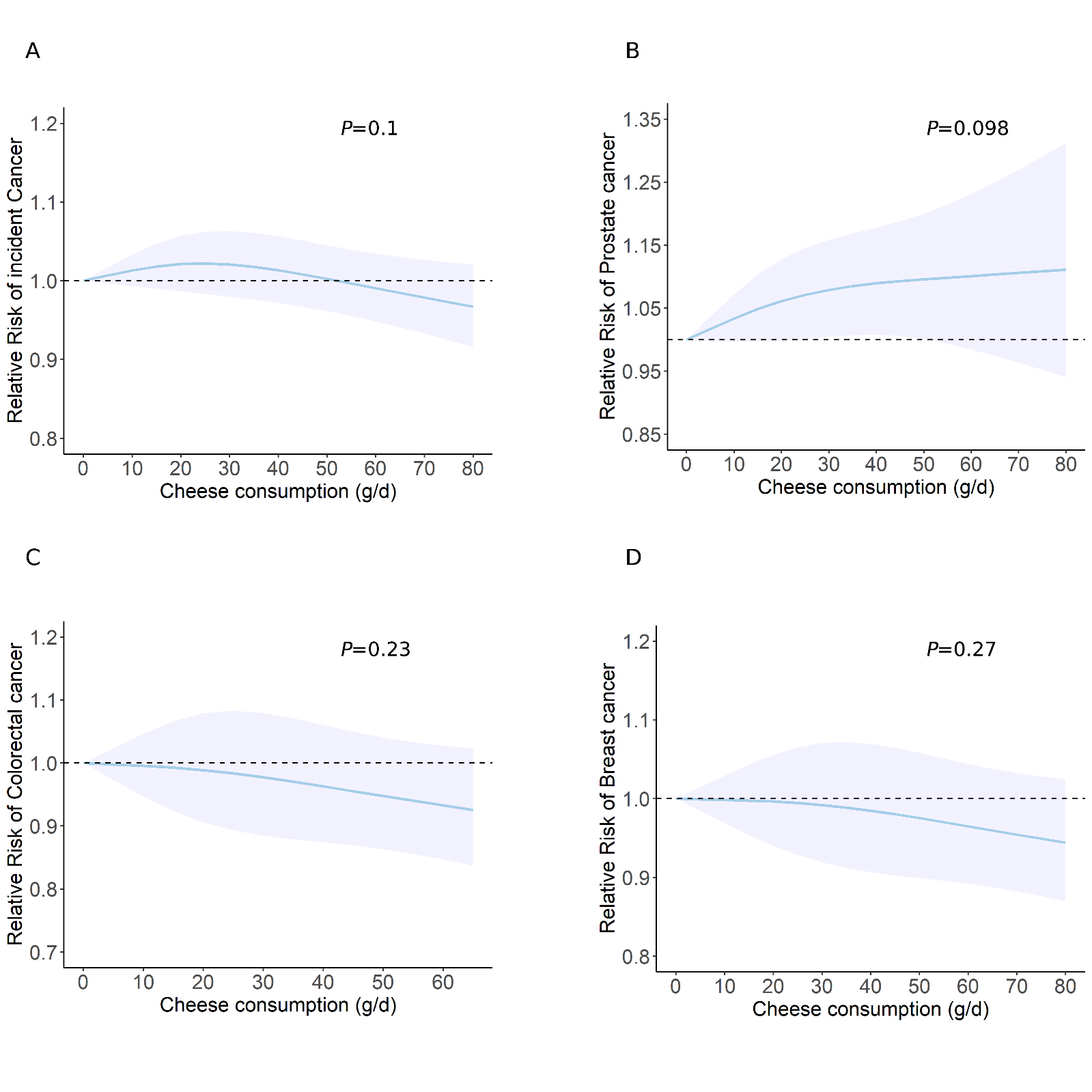


**Supplementary Figure 42. Dose-response association between cheese consumption and the risk of (A) overall cancer, (B) prostate cancer, (C) colorectal cancer and (D) breast cancer.**

**
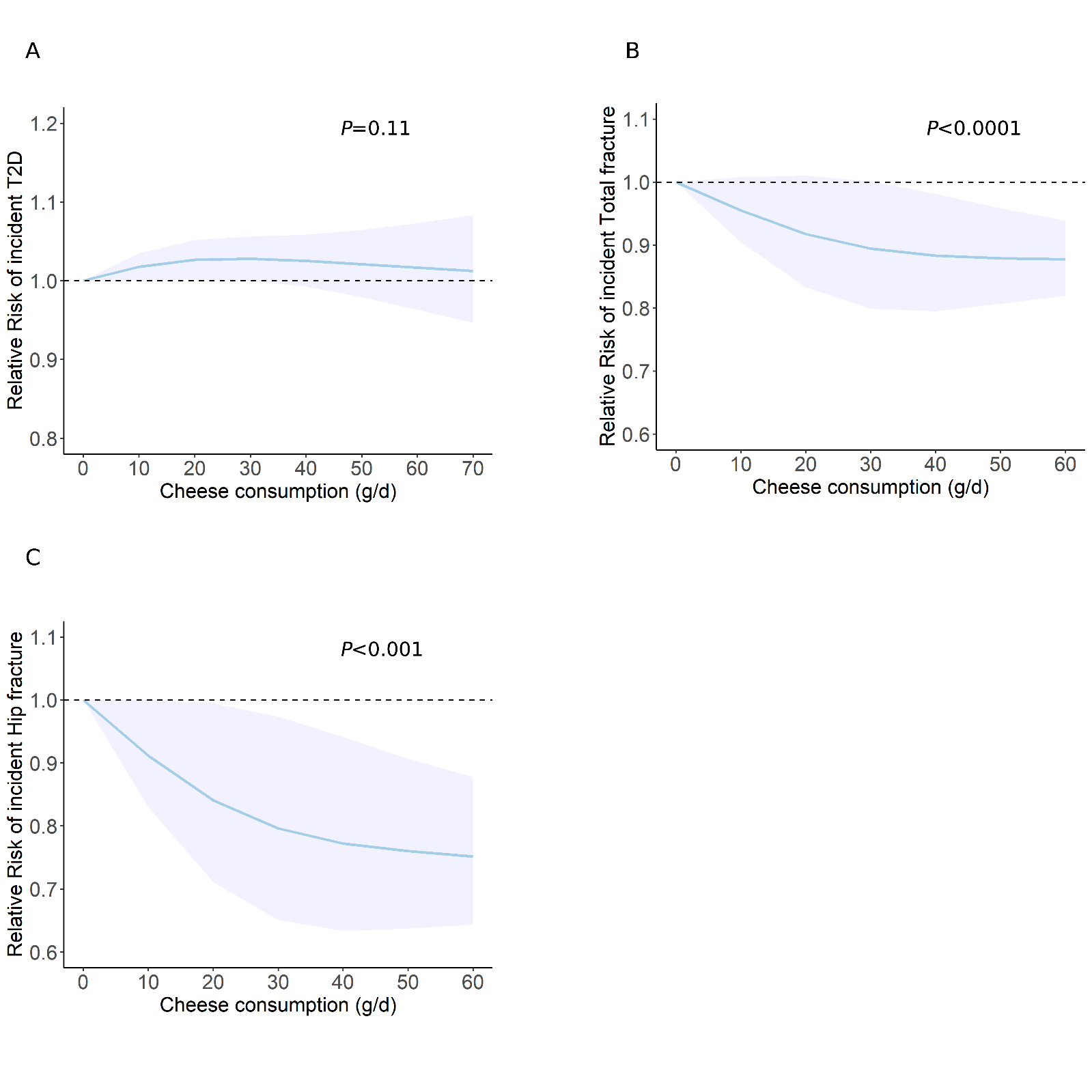
**

**Supplementary Figure 43. Dose-response association between cheese consumption and the risk of (A) type 2 diabetes, (B) total fracture and (C) hip fracture.**

T2D= type 2 diabetes.
