# Supplementary material for: Cheese consumption and multiple health outcomes: an umbrella review and updated meta-analysis of prospective studies
Source: Adv Nutr. 2023 Jun 15;14(5):1170–86. doi: 10.1016/j.advnut.2023.06.007 (PMC10509445; doi:10.1016/j.advnut.2023.06.007)
Supplement: Multimedia component4 [file mmc4.docx]

Cheese consumption and multiple health outcomes: an umbrella review and updated meta-analysis of prospective studies

Mingjie Zhang, Xiaocong Dong, Zihui Huang, Xue Li, Yue Zhao, Yingyao Wang, Huilian Zhu, Aiping Fang, Edward L. Giovannucci

**List of Supplementary Figures**

[Supplementary Figure 10. Association between cheese consumption (highest vs. lowest intake level) and overall cancer mortality. 1](#_Toc128060158)

[Supplementary Figure 11. Association between cheese consumption (per 30 g/d increment) and overall cancer mortality. 2](#_Toc128060159)

[Supplementary Figure 12. Association between cheese consumption (highest vs. lowest level of intake) and the mortality of (A) colorectal cancer, (B) colon cancer, (C) rectal cancer and (D) prostate cancer. 3](#_Toc128060160)


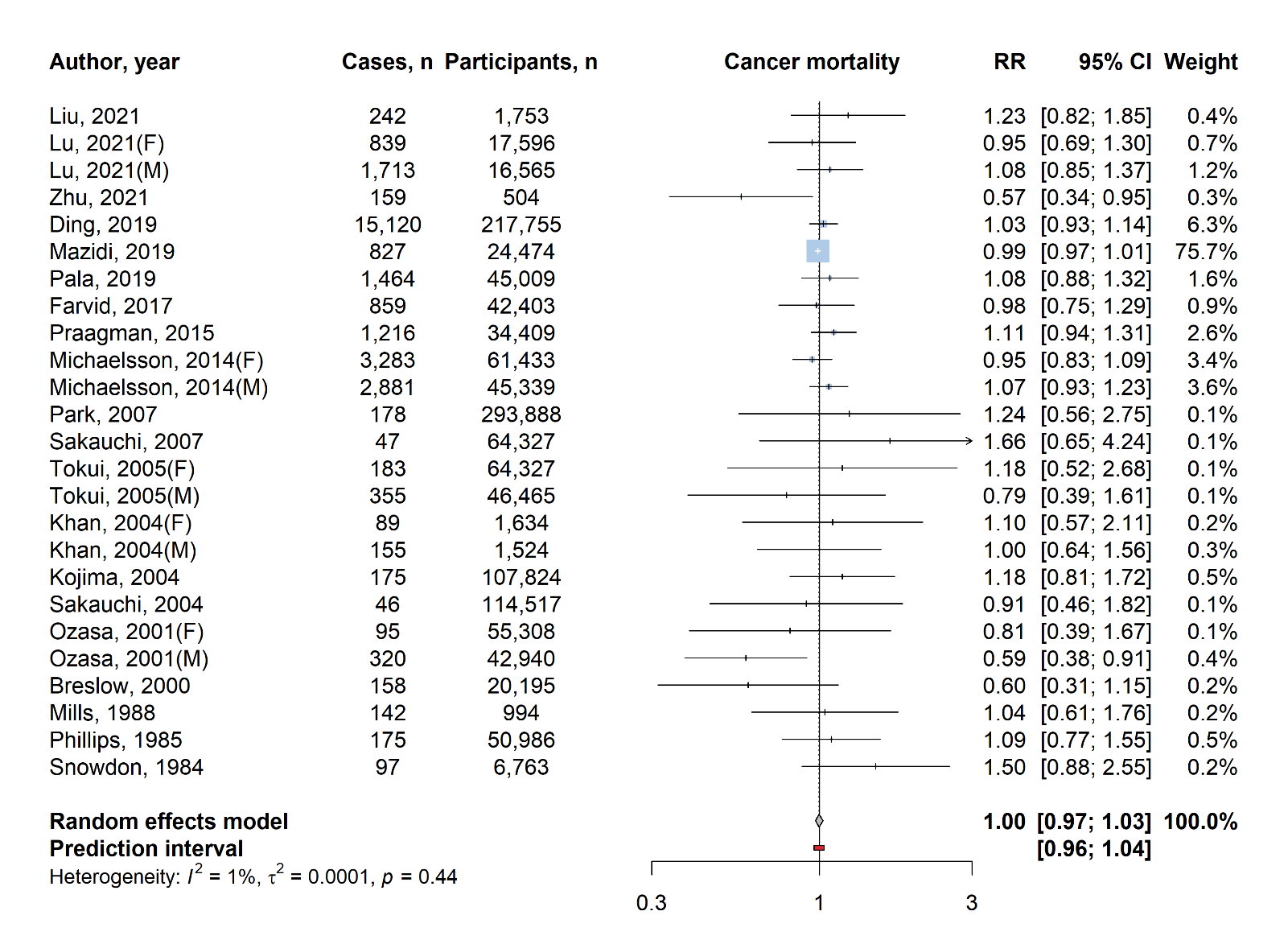


## Supplementary Figure 10. Association between cheese consumption (highest vs. lowest intake level) and overall cancer mortality.

Study-specific effect sizes are visualized in squares and the size of squares is proportional to the specific study weight to the overall meta-analysis. Horizontal lines represent 95% CIs. Diamonds demonstrate the pooled relative risk and 95% CIs. F=female; M=male.


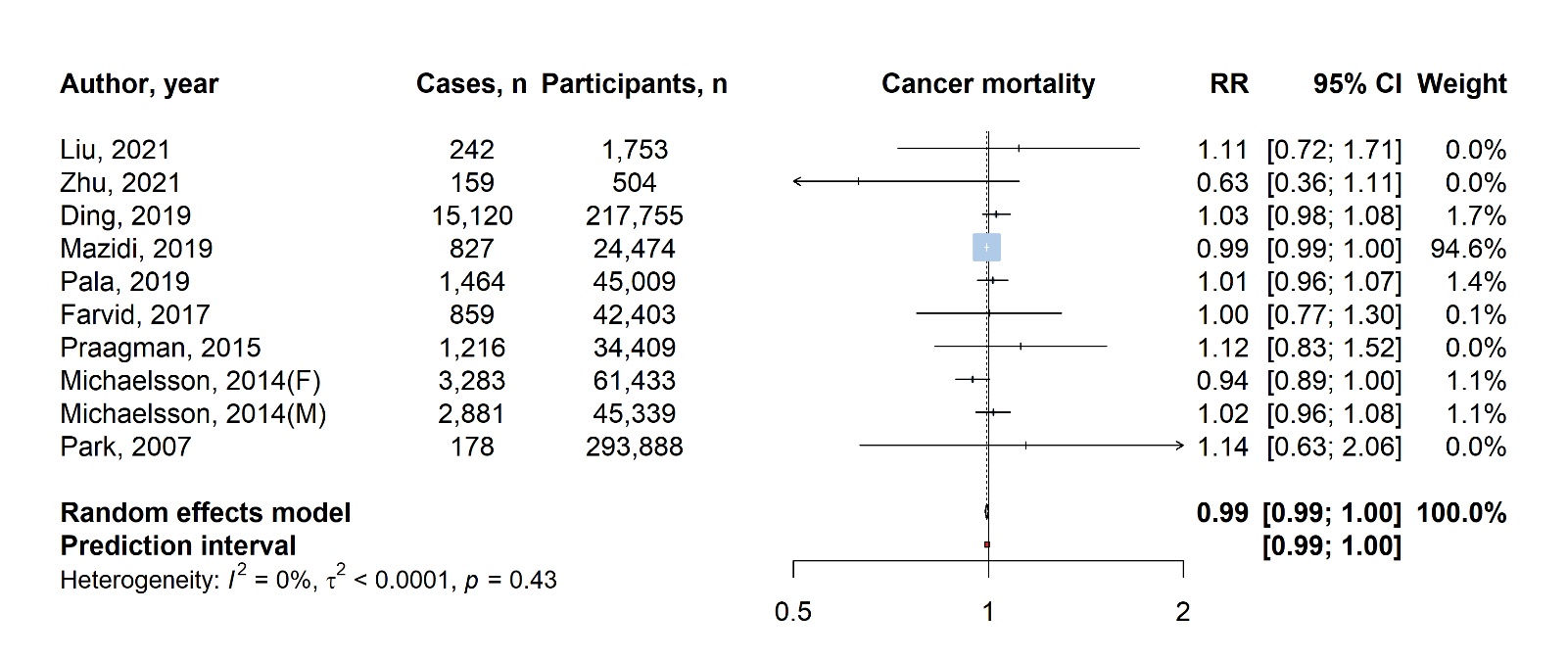


**Supplementary Figure 11. Association between cheese consumption (per 30 g/d increment) and overall cancer mortality.**

Study-specific effect sizes are visualized in squares and the size of squares is proportional to the specific study weight to the overall meta-analysis. Horizontal lines represent 95% CIs. Diamonds demonstrate the pooled relative risk and 95% CIs. F=female; M=male.


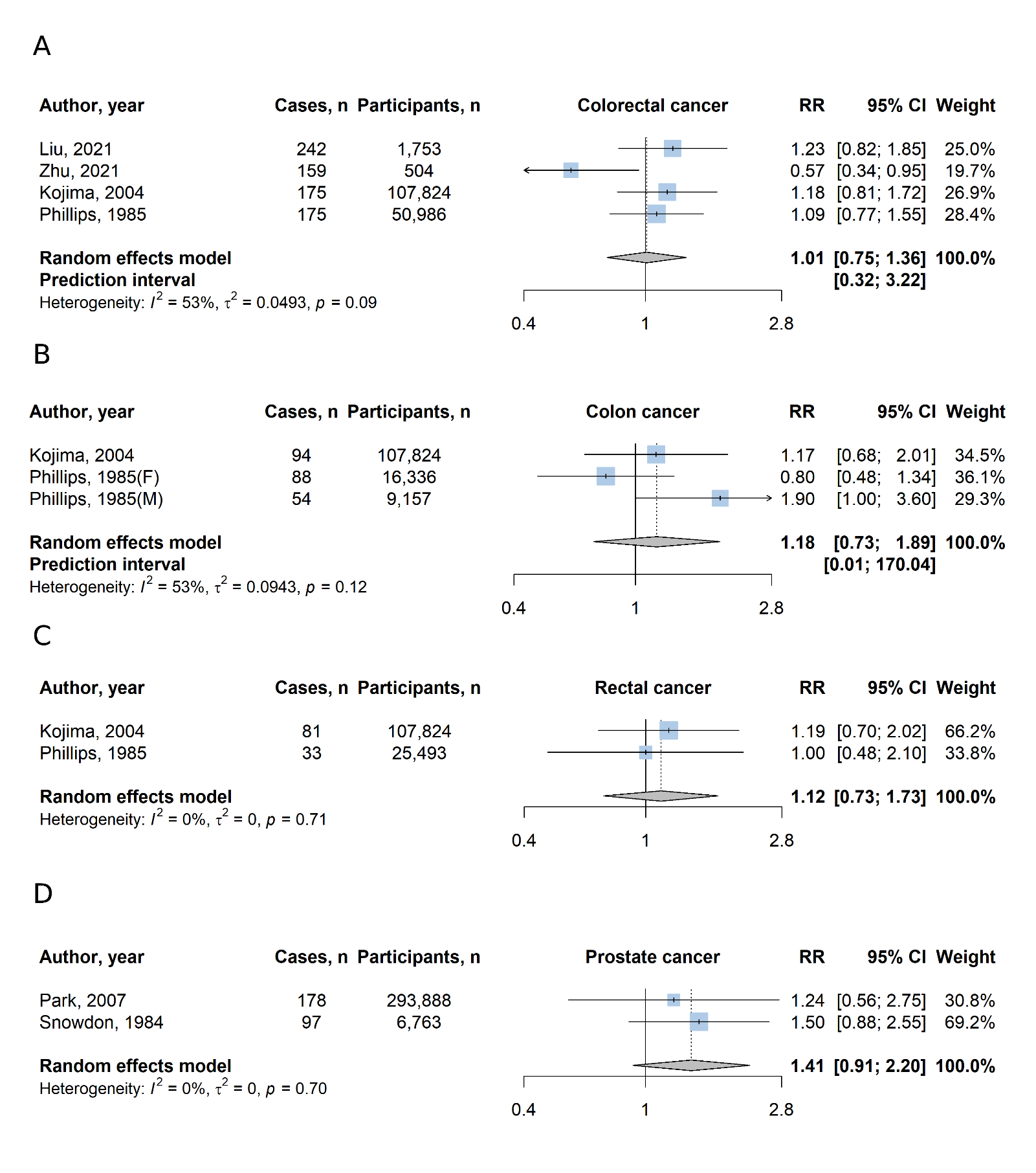


## Supplementary Figure 12. Association between cheese consumption (highest vs. lowest level of intake) and the mortality of (A) colorectal cancer, (B) colon cancer, (C) rectal cancer, and (D) prostate cancer.

Study-specific effect sizes are visualized in squares and the size of squares is proportional to the specific study weight to the overall meta-analysis. Horizontal lines represent 95% CIs. Diamonds demonstrate the pooled relative risk and 95% CIs. F=female; M=male.
