# Supplementary material for: Cheese consumption and multiple health outcomes: an umbrella review and updated meta-analysis of prospective studies
Source: Adv Nutr. 2023 Jun 15;14(5):1170–86. doi: 10.1016/j.advnut.2023.06.007 (PMC10509445; doi:10.1016/j.advnut.2023.06.007)
Supplement: Multimedia component5 [file mmc5.docx]

Cheese consumption and multiple health outcomes: an umbrella review and updated meta-analysis of prospective studies

Mingjie Zhang, Xiaocong Dong, Zihui Huang, Xue Li, Yue Zhao, Yingyao Wang, Huilian Zhu, Aiping Fang, Edward L. Giovannucci

**List of Supplementary Figures**

[Supplementary Figure 13. Association between cheese consumption (highest vs. lowest intake level) and CVD risk. 2](#_Toc128060743)

[Supplementary Figure 14. Association between cheese consumption (per 30 g/d increment) and CVD risk. 3](#_Toc128060744)

[Supplementary Figure 15. Association between cheese consumption (highest vs. lowest intake level) and CHD risk. 4](#_Toc128060745)

[Supplementary Figure 16. Association between cheese consumption (per 30 g/d increment) and CHD risk. 4](#_Toc128060746)


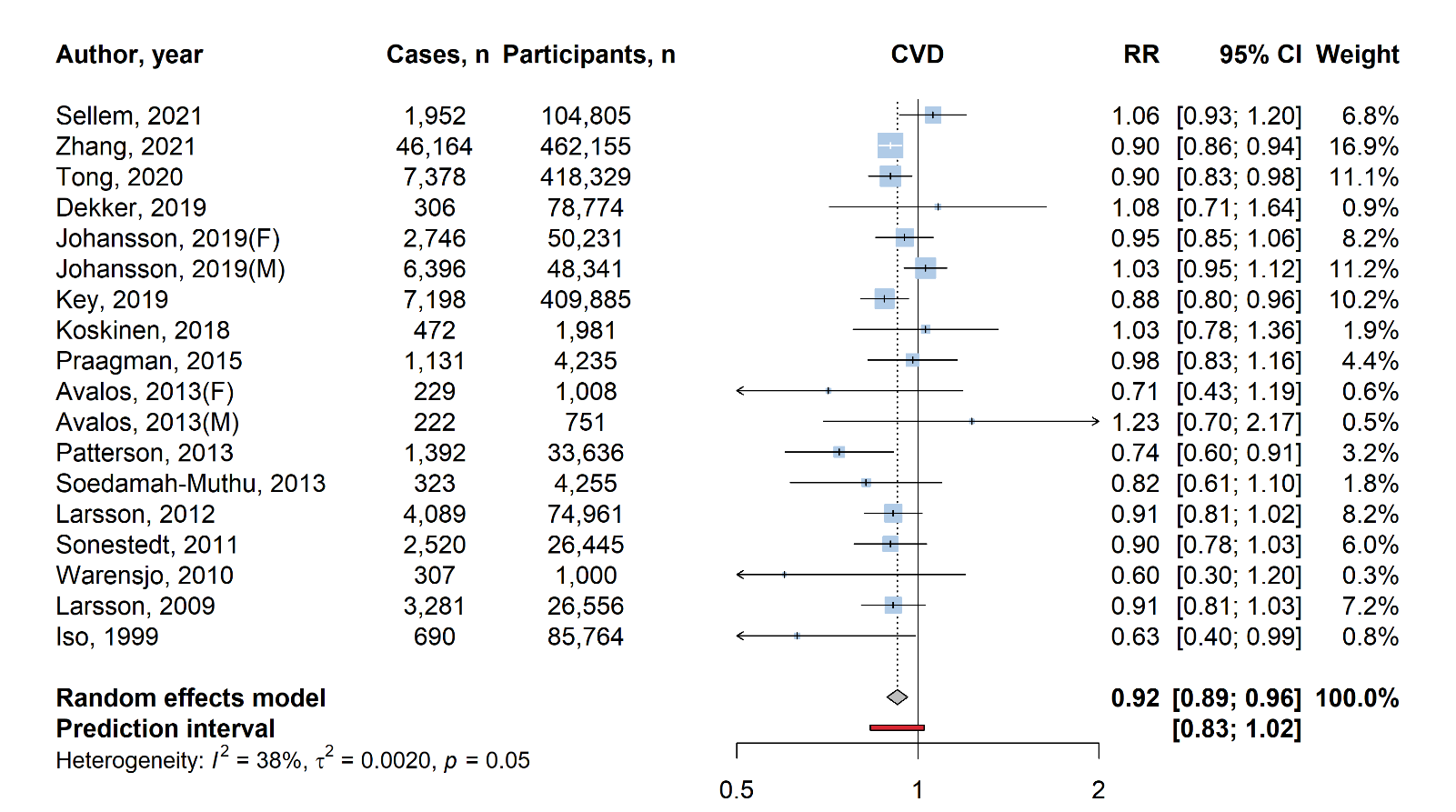


## Supplementary Figure 13. Association between cheese consumption (highest vs. lowest intake level) and CVD risk.

Study-specific effect sizes are visualized in squares and the size of squares is proportional to the specific study weight to the overall meta-analysis. Horizontal lines represent 95% CIs. Diamonds demonstrate the pooled relative risk and 95% CIs. CVD=cardiovascular disease; F=female; M=male.


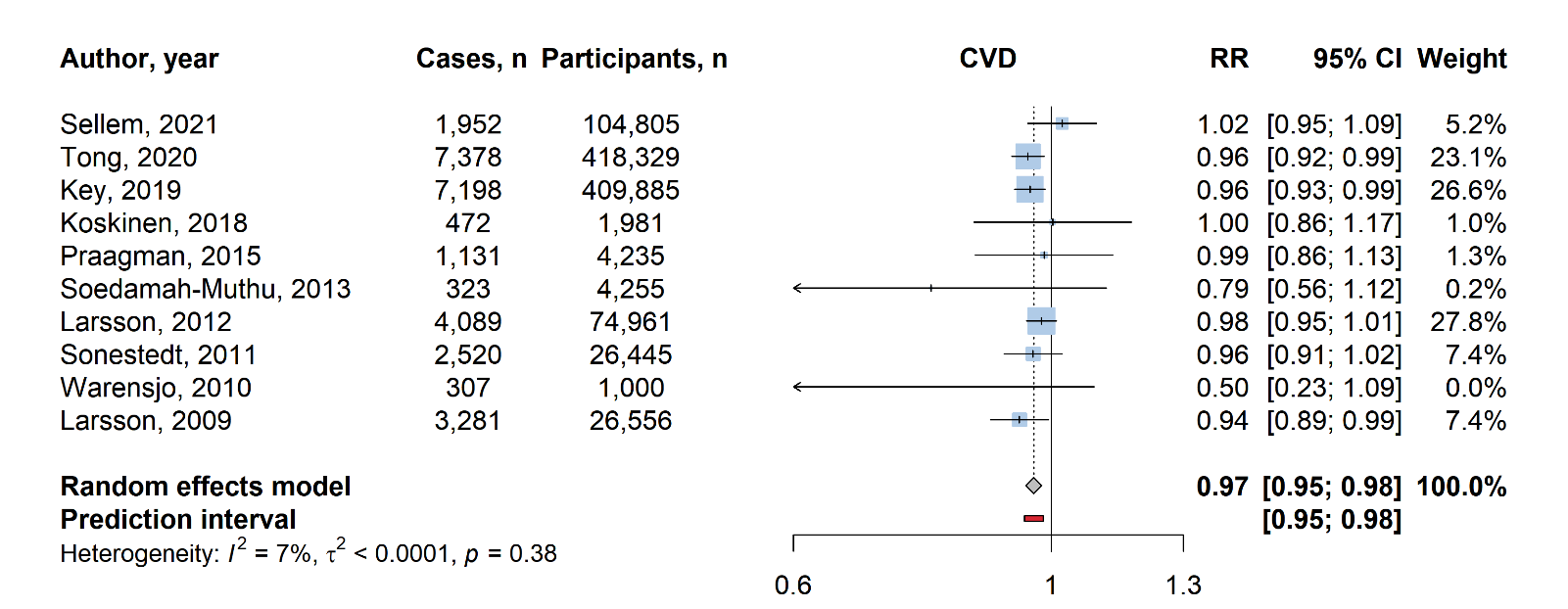


**Supplementary Figure 14. Association between cheese consumption (per 30 g/d increment) and CVD risk.**

Study-specific effect sizes are visualized in squares and the size of squares is proportional to the specific study weight to the overall meta-analysis. Horizontal lines represent 95% CIs. Diamonds demonstrate the pooled relative risk and 95% CIs. CVD=cardiovascular disease.


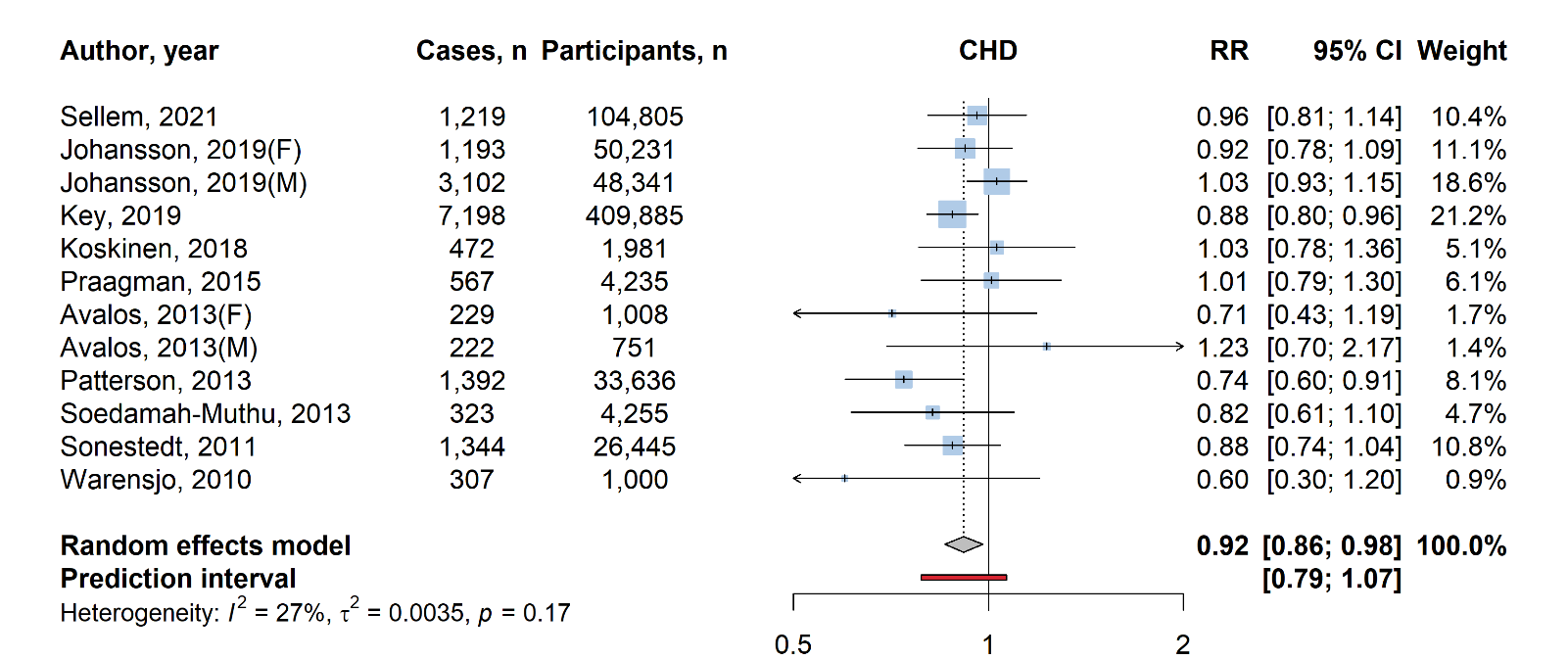


## Supplementary Figure 15. Association between cheese consumption (highest vs. lowest intake level) and CHD risk.

Study-specific effect sizes are visualized in squares and the size of squares is proportional to the specific study weight to the overall meta-analysis. Horizontal lines represent 95% CIs. Diamonds demonstrate the pooled relative risk and 95% CIs. CHD=coronary heart disease; F=female; M=male.


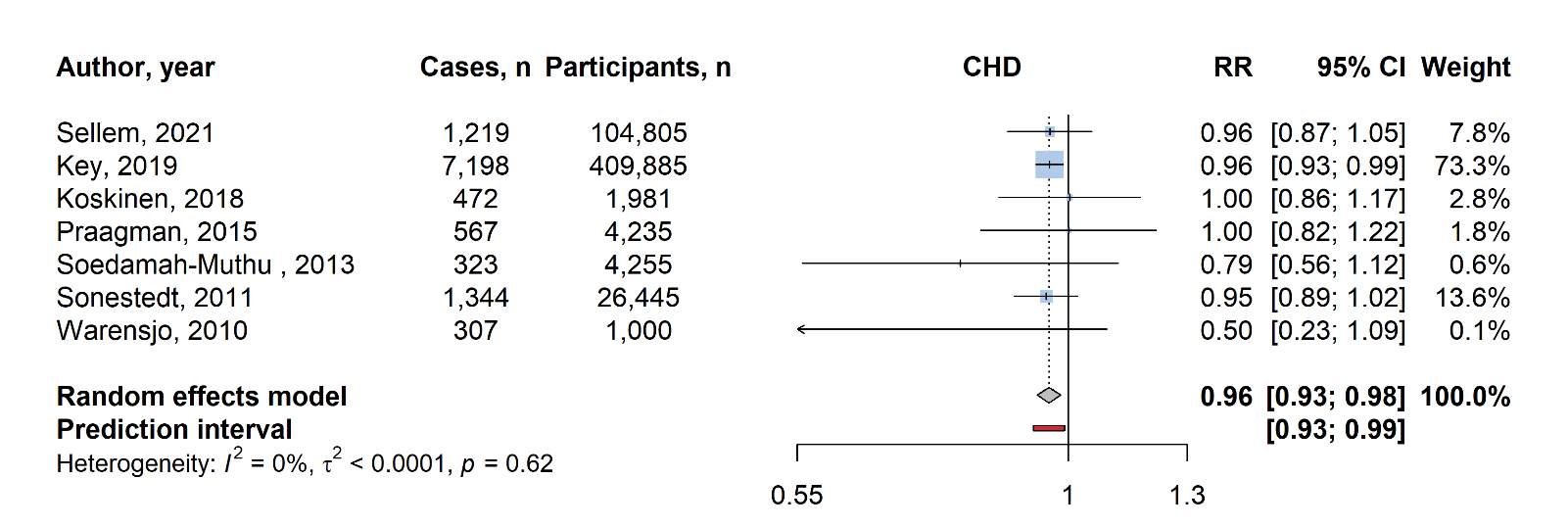


**Supplementary Figure 16. Association between cheese consumption (per 30 g/d increment) and CHD risk.**

Study-specific effect sizes are visualized in squares and the size of squares is proportional to the specific study weight to the overall meta-analysis. Horizontal lines represent 95% CIs. Diamonds demonstrate the pooled relative risk and 95% CIs. CHD=coronary heart disease.
