# Supplementary material for: Cheese consumption and multiple health outcomes: an umbrella review and updated meta-analysis of prospective studies
Source: Adv Nutr. 2023 Jun 15;14(5):1170–86. doi: 10.1016/j.advnut.2023.06.007 (PMC10509445; doi:10.1016/j.advnut.2023.06.007)
Supplement: Multimedia component6 [file mmc6.docx]

Cheese consumption and multiple health outcomes: an umbrella review and updated meta-analysis of prospective studies

Mingjie Zhang, Xiaocong Dong, Zihui Huang, Xue Li, Yue Zhao, Yingyao Wang, Huilian Zhu, Aiping Fang, Edward L. Giovannucci

**List of Supplementary Figures**

[Supplementary Figure 17. Association between cheese consumption (highest vs. lowest intake level) and stroke risk. 2](#_Toc128060983)

[Supplementary Figure 18. Association between cheese consumption (per 30 g/d increment) and stroke risk. 2](#_Toc128060984)

[Supplementary Figure 19. Association between cheese consumption (highest vs. lowest intake level) and hypertension risk. 3](#_Toc128060985)

[Supplementary Figure 20. Association between cheese consumption (per 30 g/d increment) and hypertension risk. 3](#_Toc128060986)


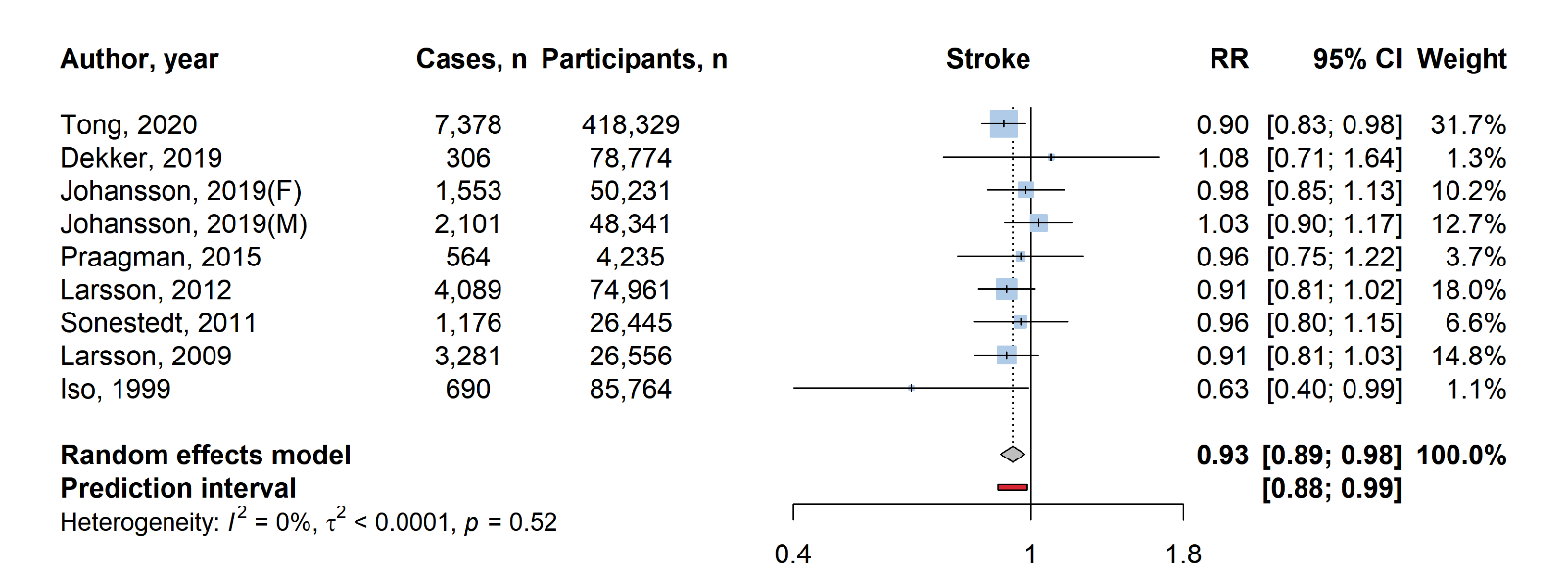


## Supplementary Figure 17. Association between cheese consumption (highest vs. lowest intake level) and stroke risk.

Study-specific effect sizes are visualized in squares and the size of squares is proportional to the specific study weight to the overall meta-analysis. Horizontal lines represent 95% CIs. Diamonds demonstrate the pooled relative risk and 95% CIs. F=female; M=male.


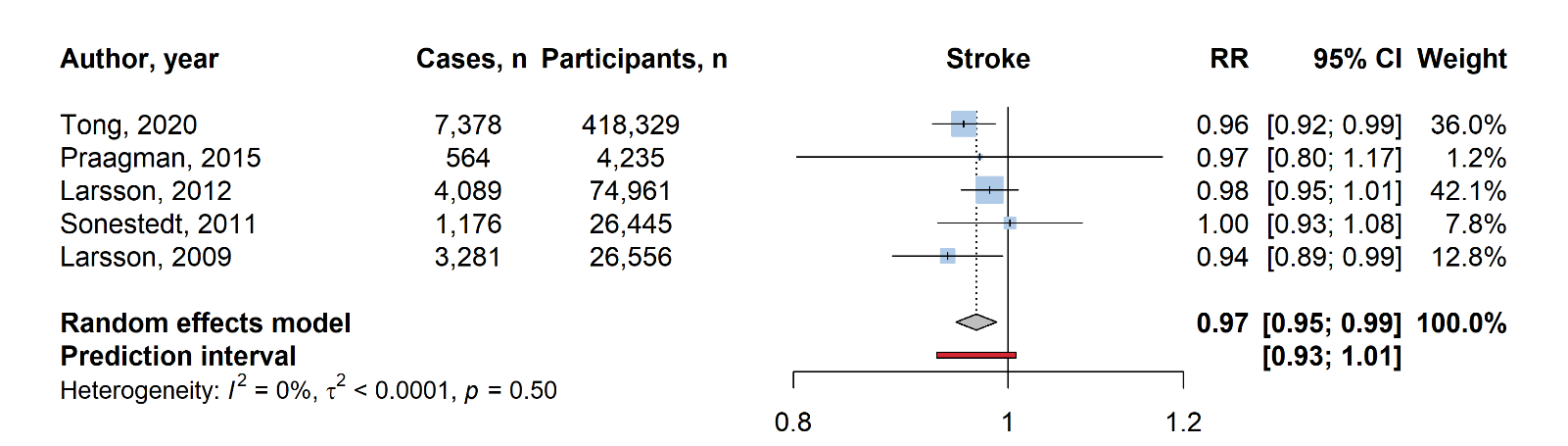


**Supplementary Figure 18. Association between cheese consumption (per 30 g/d increment) and stroke risk.**

Study-specific effect sizes are visualized in squares and the size of squares is proportional to the specific study weight to the overall meta-analysis. Horizontal lines represent 95% CIs. Diamonds demonstrate the pooled relative risk and 95% CIs. F=female; M=male.


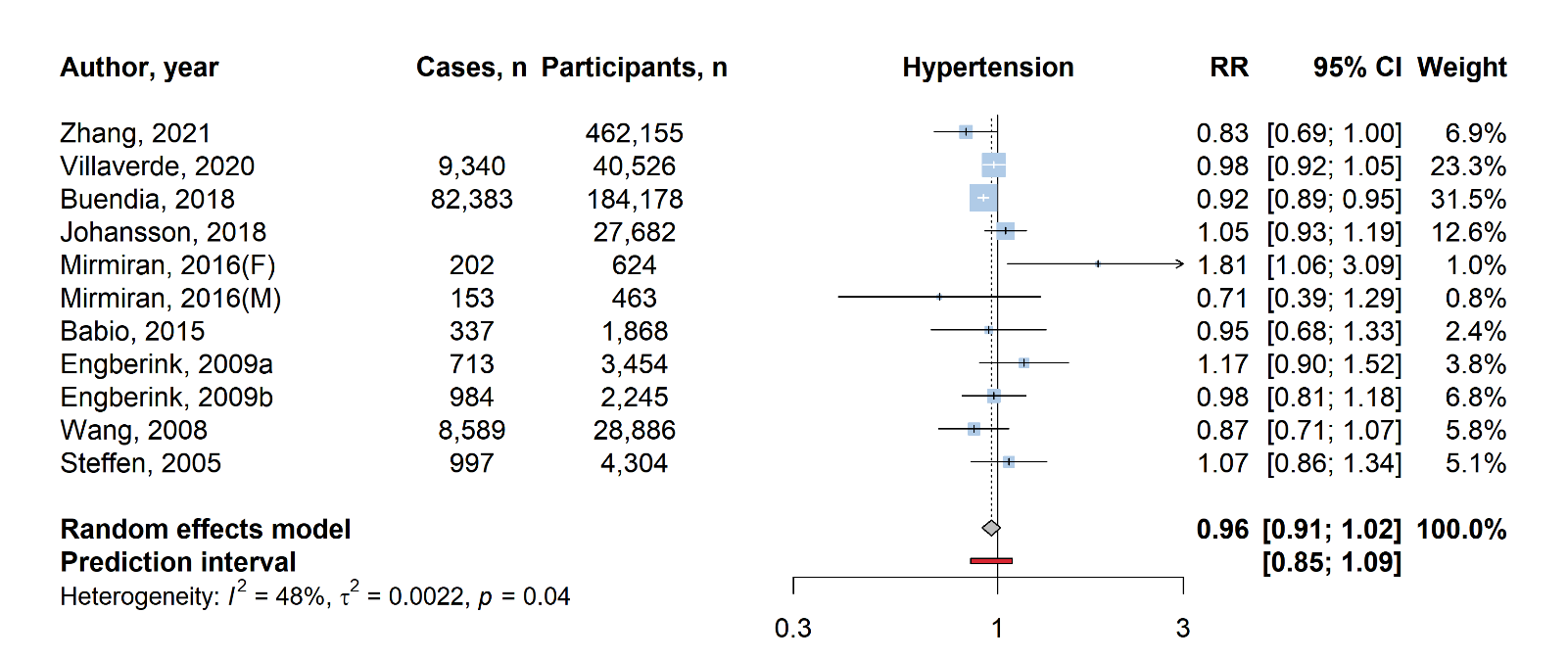


Supplementary Figure 19. Association between cheese consumption (highest vs. lowest intake level) and hypertension risk.

Study-specific effect sizes are visualized in squares and the size of squares is proportional to the specific study weight to the overall meta-analysis. Horizontal lines represent 95% CIs. Diamonds demonstrate the pooled relative risk and 95% CIs. F=female; M=male. Engberink, 2009a is the result for article “Dairy intake, blood pressure, and incident hypertension in a general Dutch population”; 2009b is the result for article “Inverse association between dairy intake and hypertension: the Rotterdam Study”.


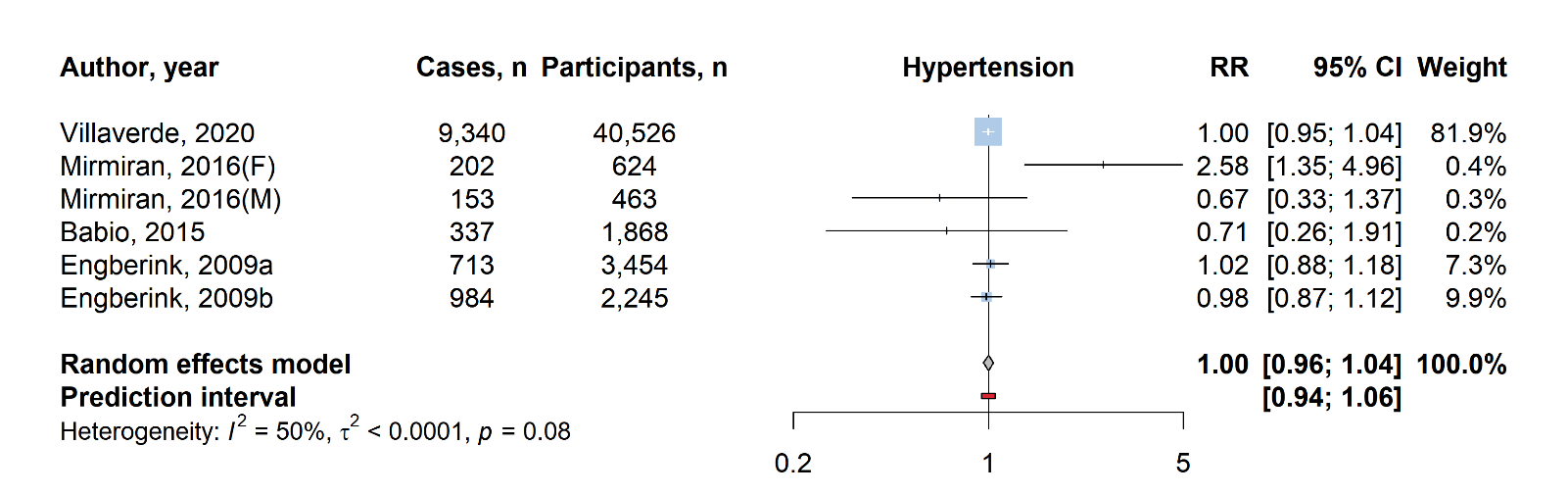


**Supplementary Figure 20. Association between cheese consumption (per 30 g/d increment) and hypertension risk.**

Study-specific effect sizes are visualized in squares and the size of squares is proportional to the specific study weight to the overall meta-analysis. Horizontal lines represent 95% CIs. Diamonds demonstrate the pooled relative risk and 95% CIs. F=female; M=male. Engberink, 2009a is the result for article “Dairy intake, blood pressure, and incident hypertension in a general Dutch population”; 2009b is the result for article “Inverse association between dairy intake and hypertension: the Rotterdam Study”.
