# Supplementary material for: Cheese consumption and multiple health outcomes: an umbrella review and updated meta-analysis of prospective studies
Source: Adv Nutr. 2023 Jun 15;14(5):1170–86. doi: 10.1016/j.advnut.2023.06.007 (PMC10509445; doi:10.1016/j.advnut.2023.06.007)
Supplement: Multimedia component7 [file mmc7.docx]

Cheese consumption and multiple health outcomes: an umbrella review and updated meta-analysis of prospective studies

Mingjie Zhang, Xiaocong Dong, Zihui Huang, Xue Li, Yue Zhao, Yingyao Wang, Huilian Zhu, Aiping Fang, Edward L. Giovannucci

**List of Supplementary Figures**

[Supplementary Figure 21. Association between cheese consumption (highest vs. lowest intake level) and overall cancer risk. 2](#_Toc128061339)

[Supplementary Figure 22. Association between cheese consumption (per 30 g/d increment) and overall cancer risk. 3](#_Toc128061340)


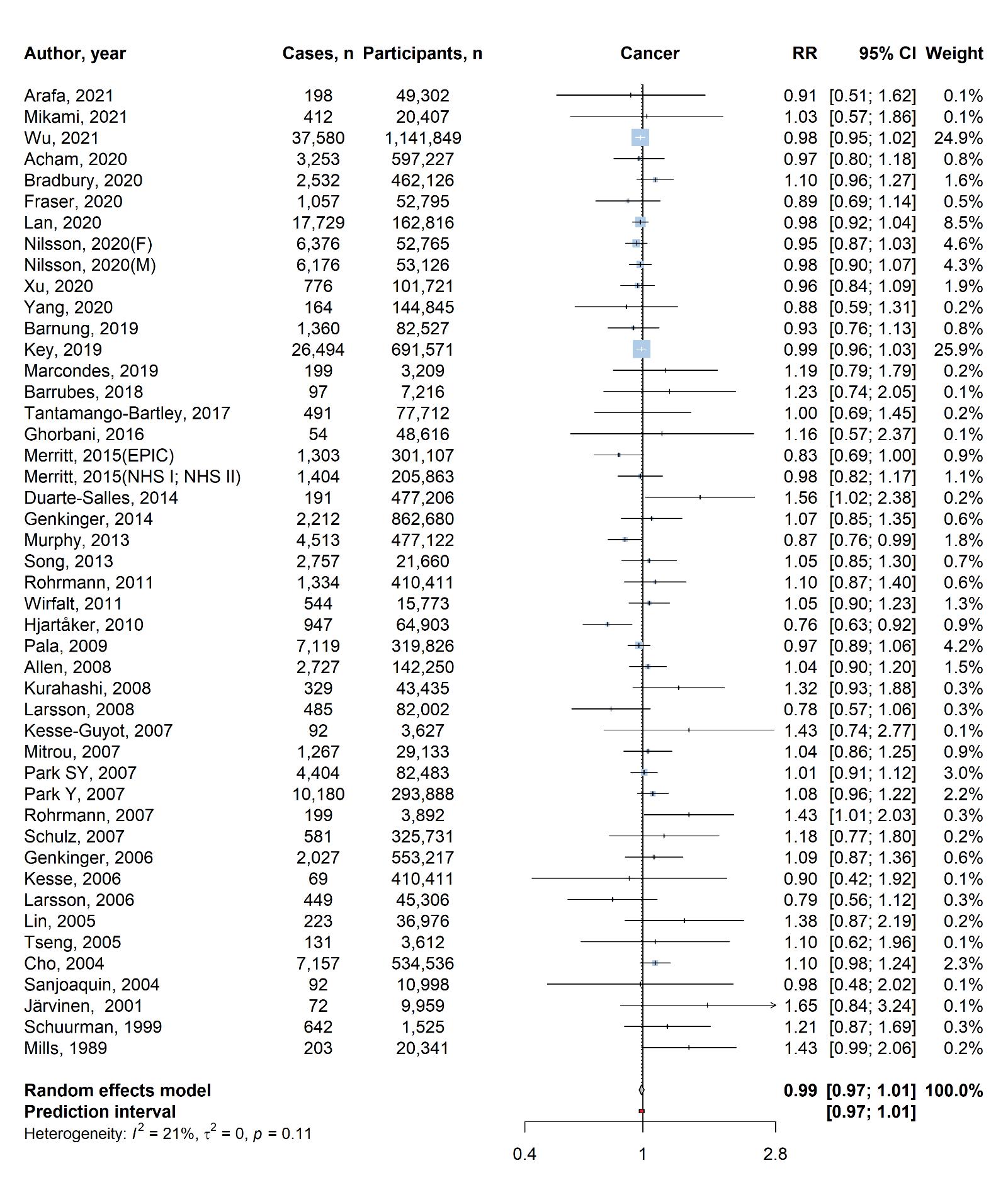


**Supplementary Figure 21. Association between cheese consumption (highest vs. lowest intake level) and overall cancer risk.**

Study-specific effect sizes are visualized in squares and the size of squares is proportional to the specific study weight to the overall meta-analysis. Horizontal lines represent 95% CIs. Diamonds demonstrate the pooled relative risk and 95% CIs. F=female; M=male; EPIC=European Prospective Investigation into Cancer and Nutrition; NHS I=Nurses’ Health Study (baseline: 1980); NHS Ⅱ= Nurses’ Health Study Ⅱ (baseline: 1991).


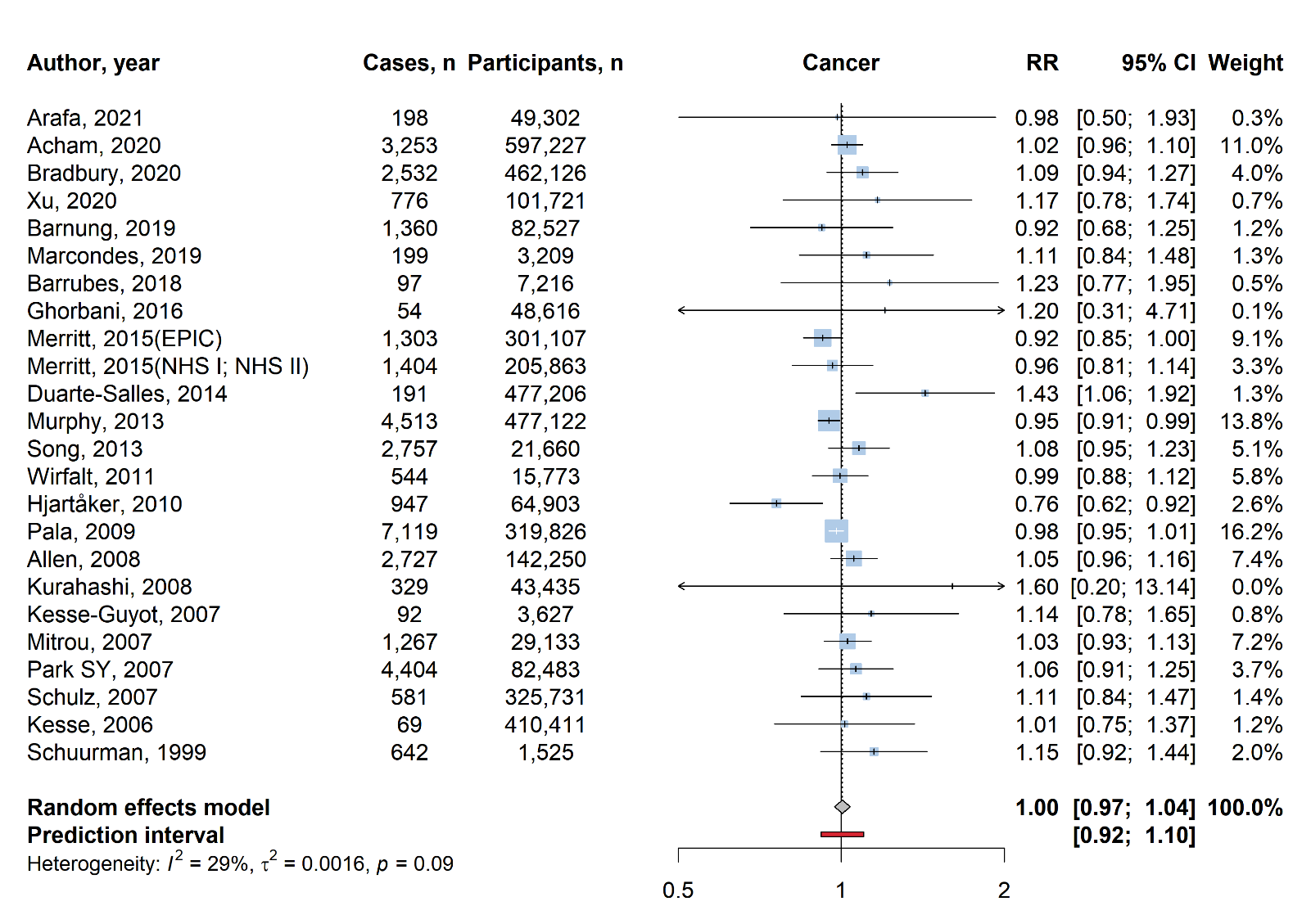


**Supplementary Figure 22. Association between cheese consumption (per 30 g/d increment) and overall cancer risk.**

Study-specific effect sizes are visualized in squares and the size of squares is proportional to the specific study weight to the overall meta-analysis. Horizontal lines represent 95% CIs. Diamonds demonstrate the pooled relative risk and 95% CIs. EPIC=European Prospective Investigation into Cancer and Nutrition; NHS I=Nurses’ Health Study (baseline: 1980); NHS Ⅱ= Nurses’ Health Study Ⅱ (baseline: 1991).
