# Supplementary material for: Cheese consumption and multiple health outcomes: an umbrella review and updated meta-analysis of prospective studies
Source: Adv Nutr. 2023 Jun 15;14(5):1170–86. doi: 10.1016/j.advnut.2023.06.007 (PMC10509445; doi:10.1016/j.advnut.2023.06.007)
Supplement: Multimedia component8 [file mmc8.docx]

Cheese consumption and multiple health outcomes: an umbrella review and updated meta-analysis of prospective studies

Mingjie Zhang, Xiaocong Dong, Zihui Huang, Xue Li, Yue Zhao, Yingyao Wang, Huilian Zhu, Aiping Fang, Edward L. Giovannucci

**List of Supplementary Figures**

[Supplementary Figure 23. Association between cheese consumption (highest vs. lowest intake level) and the risk of (A) total prostate cancer and (B) advanced prostate cancer. 2](#_Toc134885348)

[Supplementary Figure 24. Association between cheese consumption (per 30 g/d increment) and prostate cancer risk. 3](#_Toc134885349)

[Supplementary Figure 25. Association between cheese consumption (highest vs. lowest intake level) and colorectal cancer risk. 4](#_Toc134885350)

[Supplementary Figure 26. Association between cheese consumption (per 30 g/d increment) and colorectal cancer risk. 4](#_Toc134885351)


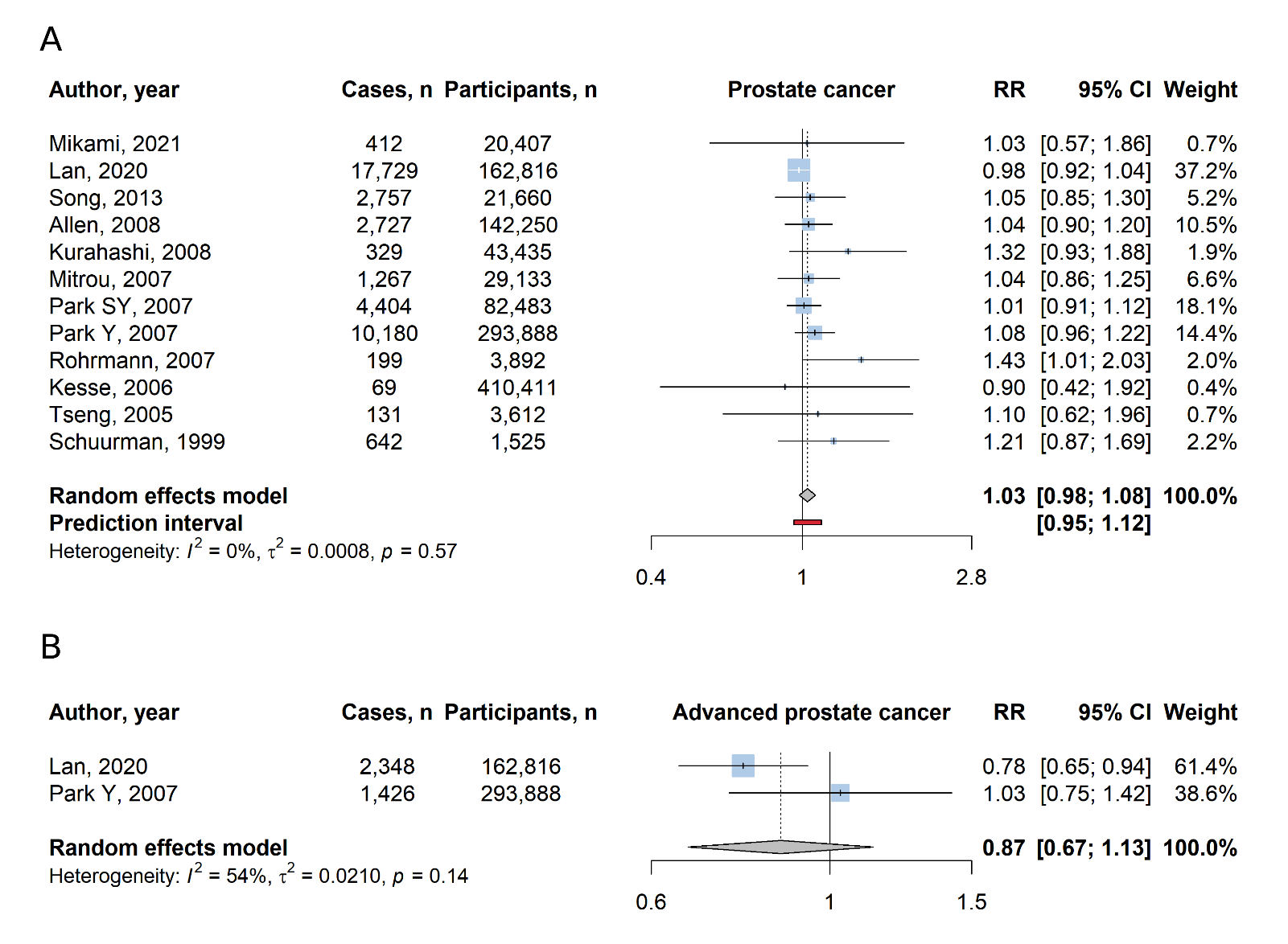


**Supplementary Figure 23. Association between cheese consumption (highest vs. lowest intake level) and the risk of (A) total prostate cancer and (B) advanced prostate cancer.**

Study-specific effect sizes are visualized in squares and the size of squares is proportional to the specific study weight to the overall meta-analysis. Horizontal lines represent 95% CIs. Diamonds demonstrate the pooled relative risk and 95% CIs.


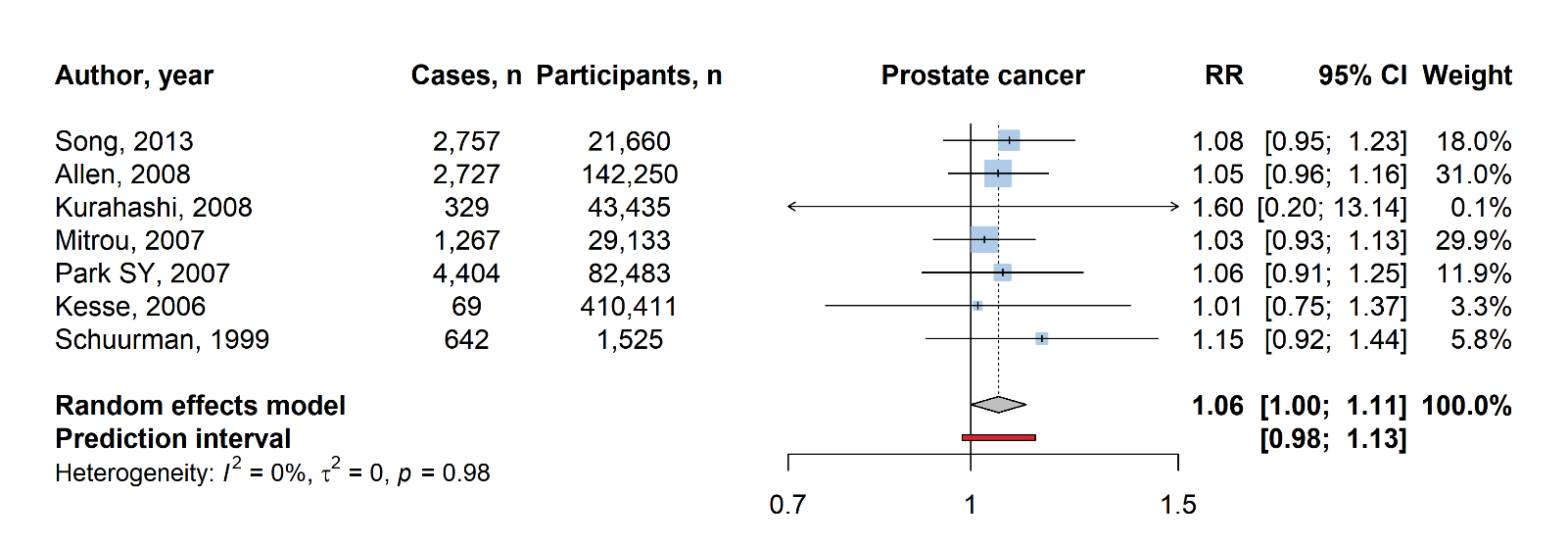


**Supplementary Figure 24. Association between cheese consumption (per 30 g/d increment) and prostate cancer risk.**

Study-specific effect sizes are visualized in squares and the size of squares is proportional to the specific study weight to the overall meta-analysis. Horizontal lines represent 95% CIs. Diamonds demonstrate the pooled relative risk and 95% CIs.


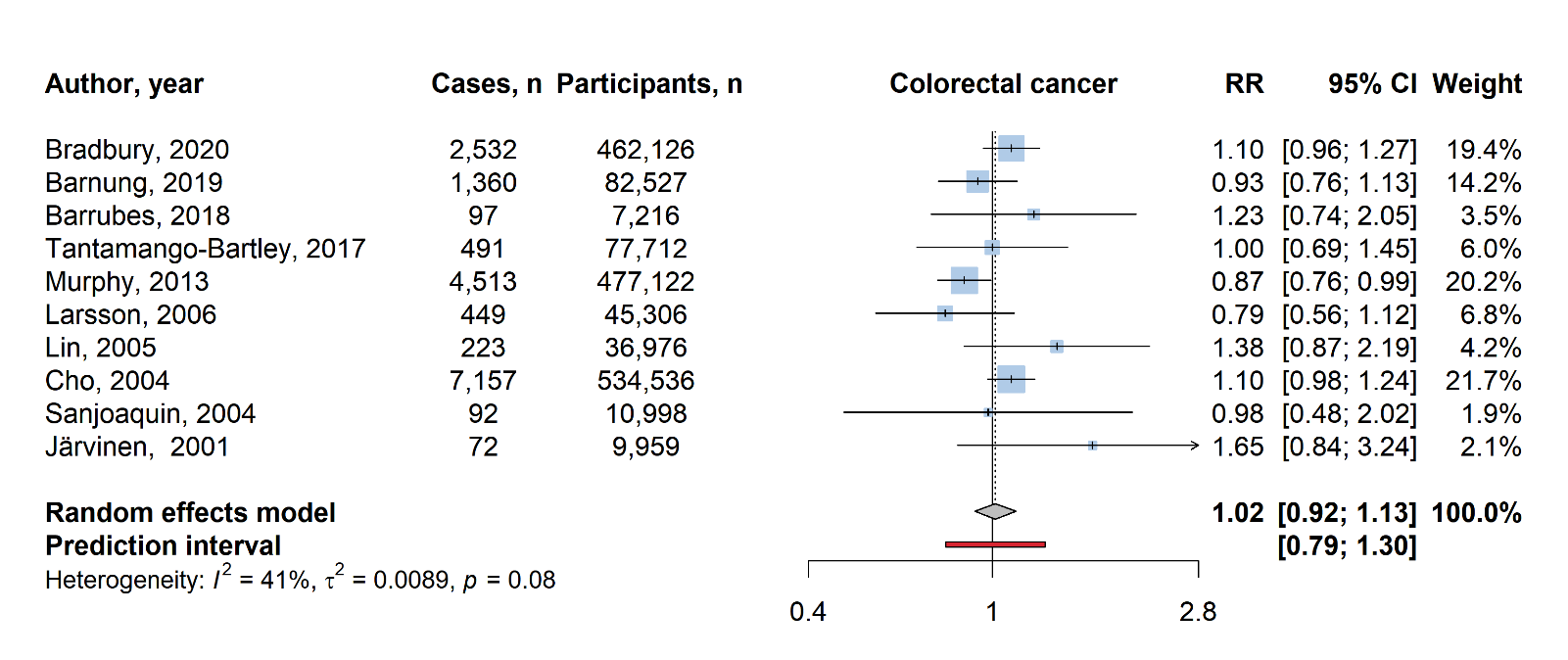


**Supplementary Figure 25. Association between cheese consumption (highest vs. lowest intake level) and colorectal cancer risk.**

Study-specific effect sizes are visualized in squares and the size of squares is proportional to the specific study weight to the overall meta-analysis. Horizontal lines represent 95% CIs. Diamonds demonstrate the pooled relative risk and 95% CIs.


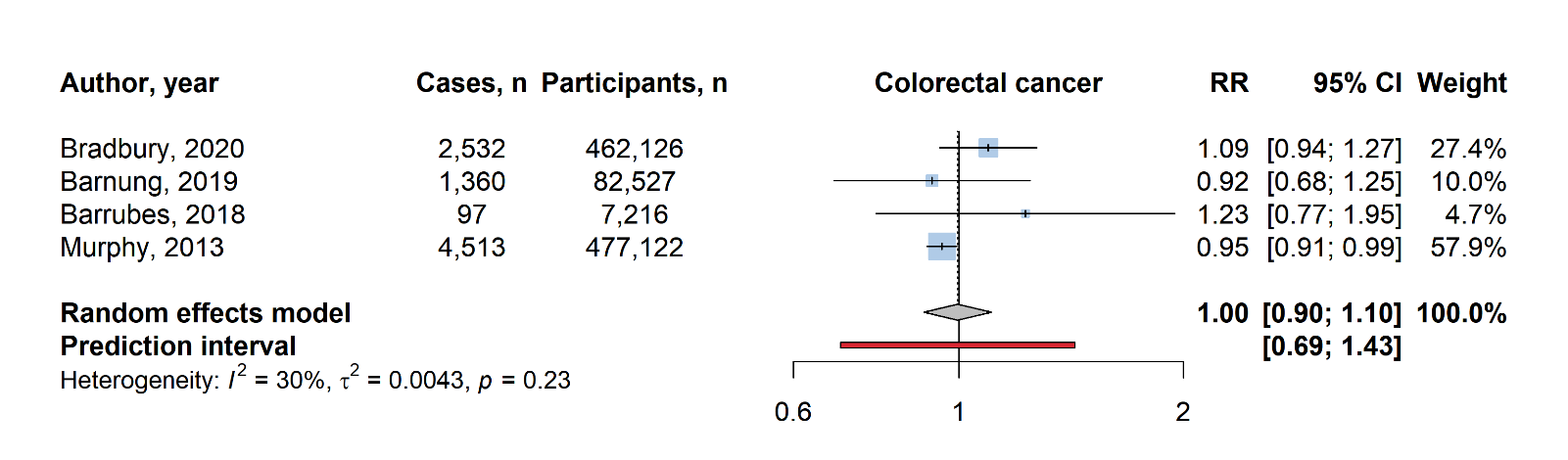


**Supplementary Figure 26. Association between cheese consumption (per 30 g/d increment) and colorectal cancer risk.**

Study-specific effect sizes are visualized in squares and the size of squares is proportional to the specific study weight to the overall meta-analysis. Horizontal lines represent 95% CIs. Diamonds demonstrate the pooled relative risk and 95% CIs.
