# Supplementary material for: Cheese consumption and multiple health outcomes: an umbrella review and updated meta-analysis of prospective studies
Source: Adv Nutr. 2023 Jun 15;14(5):1170–86. doi: 10.1016/j.advnut.2023.06.007 (PMC10509445; doi:10.1016/j.advnut.2023.06.007)
Supplement: Multimedia component9 [file mmc9.docx]

Cheese consumption and multiple health outcomes: an umbrella review and updated meta-analysis of prospective studies

Mingjie Zhang, Xiaocong Dong, Zihui Huang, Xue Li, Yue Zhao, Yingyao Wang, Huilian Zhu, Aiping Fang, Edward L. Giovannucci

**List of Supplementary Figures**

[Supplementary Figure 27. Association between cheese consumption (highest vs. lowest intake level) and the risk of (A) total colon cancer, (B) proximal colon cancer, (C) distal colon cancer, and (D) rectal cancer. 3](#_Toc134885234)


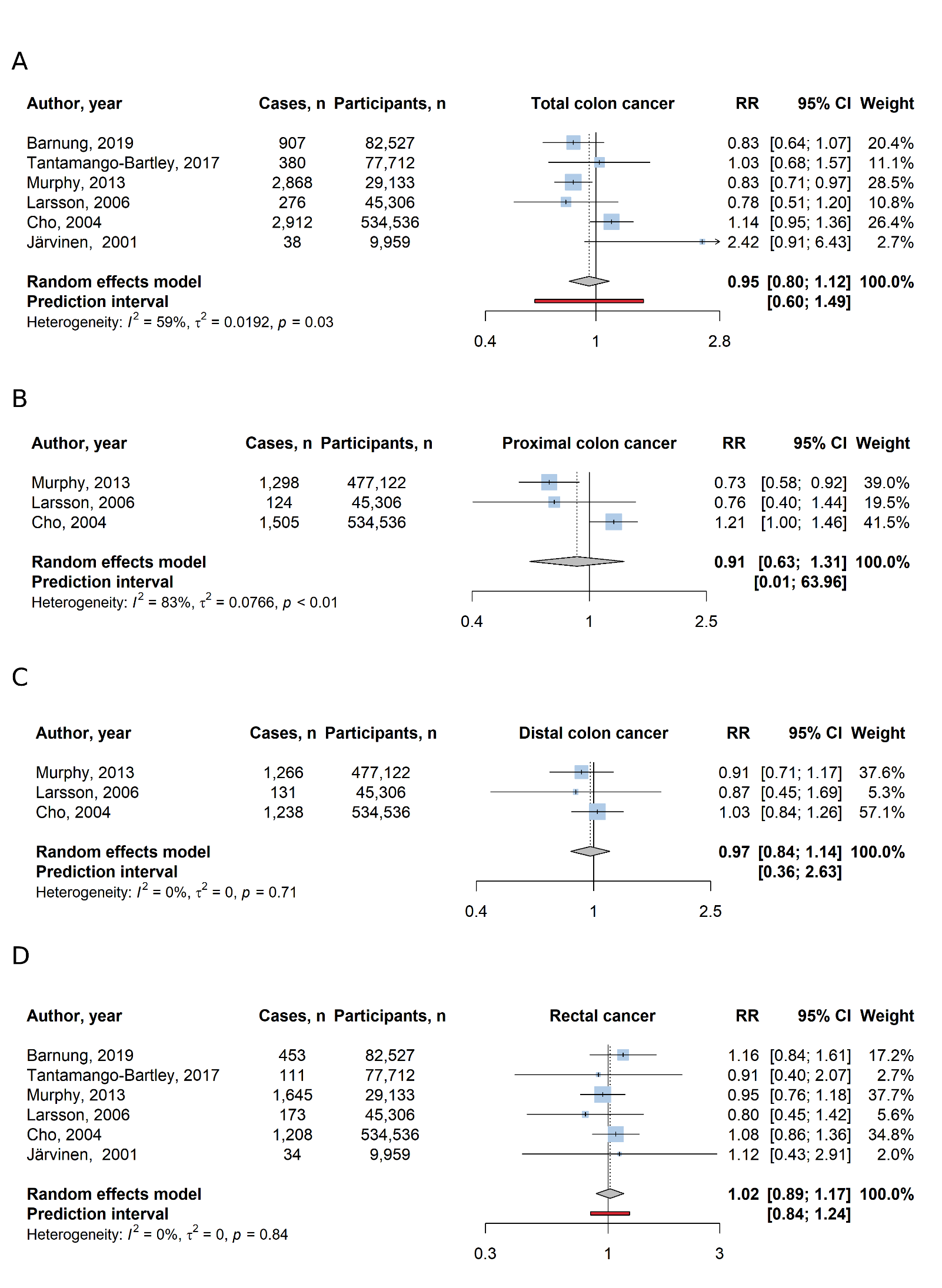


**Supplementary Figure 27. Association between cheese consumption (highest vs. lowest intake level) and the risk of (A) total colon cancer, (B) proximal colon cancer, (C) distal colon cancer, and (D) rectal cancer.**

Study-specific effect sizes are visualized in squares and the size of squares is proportional to the specific study weight to the overall meta-analysis. Horizontal lines represent 95% CIs. Diamonds demonstrate the pooled relative risk and 95% CIs.
